# RIP-Seq Suggests Translational Regulation by L7Ae in *Archaea*

**DOI:** 10.1128/mBio.00730-17

**Published:** 2017-08-01

**Authors:** Michael Daume, Michael Uhl, Rolf Backofen, Lennart Randau

**Affiliations:** a Max-Planck-Institute for Terrestrial Microbiology, Marburg, Germany; b Bioinformatics Group, Albert Ludwig University Freiburg, Freiburg, Germany; c BIOSS Centre for Biological Signaling Studies, Albert Ludwig University Freiburg, Freiburg, Germany; Medical University Innsbruck; University of Würzburg

**Keywords:** *Archaea*, RNA binding proteins, RNA structure, gene regulation

## Abstract

L7Ae is a universal archaeal protein that recognizes and stabilizes kink-turn (k-turn) motifs in RNA substrates. These structural motifs are widespread in nature and are found in many functional RNA species, including ribosomal RNAs. Synthetic biology approaches utilize L7Ae/k-turn interactions to control gene expression in eukaryotes. Here, we present results of comprehensive RNA immunoprecipitation sequencing (RIP-Seq) analysis of genomically tagged L7Ae from the hyperthermophilic archaeon *Sulfolobus acidocaldarius*. A large set of interacting noncoding RNAs was identified. In addition, several mRNAs, including the *l7ae* transcript, were found to contain k-turn motifs that facilitate L7Ae binding. *In vivo* studies showed that L7Ae autoregulates the translation of its mRNA by binding to a k-turn motif present in the 5′ untranslated region (UTR). A green fluorescent protein (GFP) reporter system was established in *Escherichia coli* and verified conservation of L7Ae-mediated feedback regulation in *Archaea*. Mobility shift assays confirmed binding to a k-turn in the transcript of *nop5-fibrillarin*, suggesting that the expression of all C/D box sRNP core proteins is regulated by L7Ae. These studies revealed that L7Ae-mediated gene regulation evolved in archaeal organisms, generating new tools for the modulation of synthetic gene circuits in bacteria.

## IMPORTANCE

L7Ae is an essential archaeal protein that is known to structure ribosomal RNAs and small RNAs (sRNAs) by binding to their kink-turn motifs. Here, we utilized RIP-Seq methodology to achieve a first global analysis of RNA substrates for L7Ae. Several novel interactions with noncoding RNA molecules (e.g., with the universal signal recognition particle RNA) were discovered. In addition, L7Ae was found to bind to mRNAs, including its own transcript’s 5′ untranslated region. This feedback-loop control is conserved in most archaea and was incorporated into a reporter system that was utilized to control gene expression in bacteria. These results demonstrate that L7Ae-mediated gene regulation evolved originally in archaeal organisms. The feedback-controlled reporter gene system can easily be adapted for synthetic biology approaches that require strict gene expression control.

## INTRODUCTION

The archaeal protein L7Ae and its eukaryotic homologs, e.g., L30e and 15.5 kD, are members of the L7Ae/L30 protein family (1–3). These proteins recognize a distinctive RNA motif, termed kink-turn (k-turn or Kt), and mediate RNA structure formation (4, 5). The standard k-turn motif is characterized by a short stem followed by an asymmetric three-nucleotide (3-nt) bulge and two conserved *trans* sugar-Hoogsteen G · A pairs, generating a tight kink in the folded RNA (4, 6). These k-turn motifs are found in a large variety of RNAs, including rRNA and s(no)RNAs (4, 7). Accordingly, L7Ae/L30 proteins are components of the large ribosome subunit and are associated with C/D box and H/ACA box s(no)RNPs (7–10). C/D box sRNPs are ribonucleoprotein complexes that are composed of C/D box small RNAs (sRNAs) and the three archaeal proteins L7Ae, Nop5, and fibrillarin (11, 12). They guide the site-specific 2′-*O*-methylation of rRNA, tRNA, and other RNA species or act as chaperones to ensure proper rRNA folding (13, 14). The conserved C boxes (consensus sequence, 5′-RUGAUGA-3′) and D boxes (consensus sequence, 5′-CUGA-3′) of C/D box sRNAs form k-turn structures, and the two flanking guide regions show complementarity to rRNA or tRNA, which directs the methylase fibrillarin to a specific ribose of the RNA target (7, 13). Methylation increases rRNA stability as evidenced by high numbers of C/D box sRNA genes in hyperthermophilic archaea (14, 15). Additionally, archaeal L7Ae was found to be an integral part of the tRNA maturation complex RNase P and contacts with the universal signal recognition particle (SRP) RNA are suggested (16–18).

Due to the high specificity and affinity of L7Ae with respect to modular k-turn structures, this interaction was utilized in synthetic biology approaches. For example, Saito and coworkers designed a synthetic L7Ae/k-turn ON/OFF switch to control the translation of an output protein in human cells (19). Subsequently, this tool was developed to function in complex synthetic circuits for human cell fate control or feedback regulation of proteins in mammalian cells (20–22).

In this report, we describe a global RNA immunoprecipitation sequencing (RIP-Seq) approach for identification of unknown interaction partners of the L7Ae protein from *Sulfolobus acidocaldarius*. L7Ae was found to mediate (i) noncoding RNA structuring and (ii) translational regulation. A reporter system was established which allows the screening of L7Ae/k-turn interactions in synthetic gene circuits.

## RESULTS

### The L7Ae-RNA interactome of *Sulfolobus acidocaldarius.*

**FIG 1:**
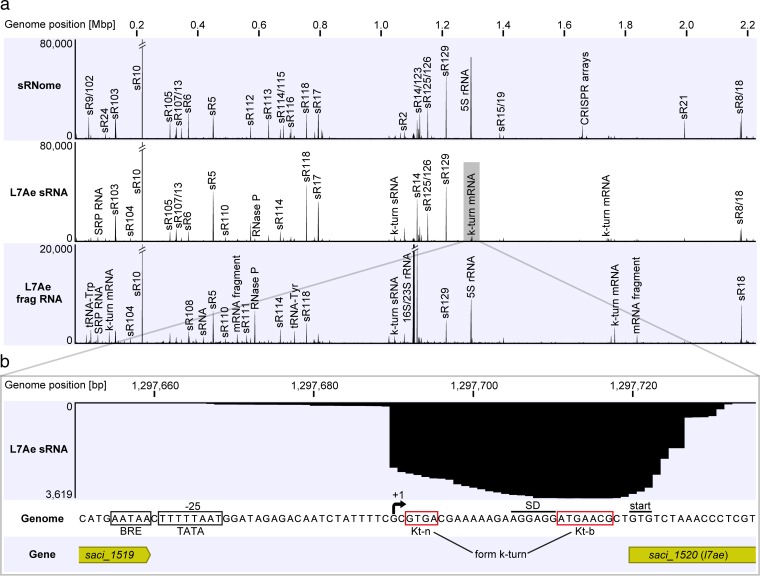
The small RNome and L7Ae-RNA interactome of *S. acidocaldarius*. (a) Small RNAs (sRNome track) and L7Ae-interacting RNAs (L7Ae sRNA and L7Ae frag RNA tracks) of *S. acidocaldarius* were subjected to Illumina RNA-Seq. Coverage plots highlight the most abundant RNA reads as distinct peaks. Peaks representing C/D box sRNAs are labeled by (Sac-)sR number in accordance with previous studies (12, 14). Each RNA profile contained one million mapped reads. (b) The coverage plot of the *l7ae* promoter region of the L7Ae sRNA track is shown. A high number of reads was found downstream of position 1297690, which marks the transcriptional start site (+1) of the 5′ UTR of *l7ae*. Motifs for the BRE (B recognition element) and TATA sites are boxed upstream of the transcriptional start site. A Shine-Dalgarno (SD) motif is present 9 nt upstream of the GTG start codon, and k-turn-forming Kt-n and Kt-b strands are marked.

RNA sequencing (RNA-Seq) was performed to identify the sRNome (the complete set of cellular sRNAs) of *S. acidocaldarius* during logarithmic growth. Total and small RNAs were isolated from *S. acidocaldarius* MW001 cells, and the sRNA fraction was subjected to Illumina sequencing (see Fig. S1 in the supplemental material). The single *l7ae* gene (*saci_1520*) in the genome of *S. acidocaldarius* was mutated to allow the production of L7Ae with a C-terminal Flag-hemagglutinin (Flag-HA) tag. The growth of the recombinant strain was not influenced by the presence of this tag (Fig. S2a). L7Ae was purified via two consecutive immunoprecipitation (IP) steps, and known protein interaction partners, including Nop5 and fibrillarin, were coisolated (Fig. S2b). The isolate also contained large amounts of coimmunoprecipitated RNA species that ranged in size from less than 50 nt to more than 1,000 nt (Fig. S2c). Northern blot analysis verified the presence of C/D box sRNAs within the isolate (Fig. S2d). The coimmunoprecipitated RNAs were subjected to small-RNA sequencing (RIP-Seq) to obtain the L7Ae-RNA interactome of *S. acidocaldarius*. One RNA sample was fragmented using ZnCl_2_ in order to sequence longer RNAs. A total of 70 million obtained sequencing reads were mapped onto the *S. acidocaldarius* genome. The sRNome plot revealed that C/D box sRNAs were highly abundant in the cell (Fig. 1a). A single C/D box sRNA, Sac-sR10, was found to be most abundant within the C/D box sRNA population. One guide region of this RNA is proposed to target tRNAs (Gly-CCC, Pro-CGG, Pro-GGG) (12). Detailed analysis of the sRNome revealed 405 sRNAs that were present during logarithmic growth: 48 tRNAs, 1 SRP RNA, 1 RNase P RNA, 1 5S rRNA, 62 C/D box sRNAs, 2 H/ACA box sRNAs, 223 CRISPR RNAs, 46 antisense RNAs, and 21 sRNAs of unknown function (see Table S1 in the supplemental material). In agreement with the sRNome analysis, the two RIP-Seq plots of L7Ae displayed high read numbers for the C/D box sRNAs (Fig. 1a). Ribosomal RNAs and RNase P RNA are other known L7Ae interaction partners that were overrepresented in the plots. In addition, abundant reads were found for the SRP RNA and for several mRNA fragments. A peak calling analysis of the RIP-Seq data sets was performed for the comprehensive identification of L7Ae interaction partners. Using a control purification of untagged L7Ae, we found 107 enriched RNAs for the L7Ae RIP-Seq data sets (Table S1): 59 C/D box sRNAs, 1 5S rRNA, 1 16S rRNA, 1 23S rRNA, 1 RNase P RNA, 1 SRP RNA, 1 tRNA, 1 H/ACA box sRNA, 3 antisense RNAs, 6 unknown sRNAs, and 32 mRNA fragments. Interestingly, many of the mRNA fragments comprised sequence motifs that matched or resembled the consensus sequence of C and D boxes. In order to delineate the terminology of the C/D box sRNAs, the identified C and D box-like motifs were termed the Kt-b (bulged) strand and the Kt-n (nonbulged) strand, respectively. The presence of these mRNAs might therefore have been the result of the presence of k-turn motifs that were identified and bound by L7Ae. Postulated k-turn motifs were found both in the coding sequences of mRNA and in untranslated regions (UTRs), e.g., in the 5′ UTR of *saci_1520* and *saci_1468* or in the coding sequence of *saci_1347* and *saci_2027*.

10.1128/mBio.00730-17.3 FIG S1  Isolation of *S. acidocaldarius* total and small RNA. The left panel shows a native agarose gel of a total RNA (t) sample depleted of small RNAs. Two definite bands that represent 16S and 23S rRNAs are visible. Their migration behavior reflects the compact folding of rRNA from hyperthermophilic organisms. The right panel shows a small-RNA (s) isolate that was separated via denaturing PAGE. Bands of 60 to 120 nt that represent different species of small RNAs from *S. acidocaldarius* are visible. Download FIG S1, PDF file, 0.9 MB. Copyright © 2017 Daume et al. 2017 Daume et al. This content is distributed under the terms of the Creative Commons Attribution 4.0 International license.

10.1128/mBio.00730-17.4 FIG S2  Growth curves of *S. acidocaldarius* strains with Flag-HA-tagged L7Ae and coimmunoprecipitation of L7Ae-bound proteins and RNA. (a) The growth behavior of the *S. acidocaldarius* MW001 reference strain and the genomically Flag-HA-tagged L7Ae strain is shown. Three biological replicates were tested. Error bars (standard deviations) are depicted as color-filled areas. (b) SDS-PAGE analysis shows the final purification fraction of L7Ae from *S. acidocaldarius* after two consecutive immunoprecipitation (Flag-IP and HA-IP) steps. Lanes 1 to 3 represent three biological replicates and show the coimmunoprecipitation of the L7Ae-interacting proteins Nop5 and fibrillarin and other proteins (identified via mass spectrometry). The mock purification of a culture without Flag-HA-tagged L7Ae (WT) shows no L7Ae or interacting proteins. (c) The gel image shows the denaturing PAGE of the final L7Ae purification step described for panel b, which displays L7Ae-interacting RNA species in all sizes. Distinct bands of sizes between 50 and 80 nt are visible. No visible amounts of nucleic acid are present in the mock-purification (WT) fraction. (d) The left panel shows the results of denaturing PAGE of L7Ae coimmunoprecipitated RNA (L7Ae). The mock purification (WT) and small-RNA isolation (sRNA) results from *S. acidocaldarius* were applied as negative (NegC) and positive (PosC) controls, respectively. As shown in the right panel, the gel was used for Northern blot analysis. A radioactively labeled probe against C/D box sRNA Sac-sR10 was utilized for C/D box sRNA detection. Signals are detected in the L7Ae sample and the positive control. No signal was present in the negative control. Download FIG S2, PDF file, 2.5 MB. Copyright © 2017 Daume et al. 2017 Daume et al. This content is distributed under the terms of the Creative Commons Attribution 4.0 International license.

10.1128/mBio.00730-17.9 TABLE S1  List of sRNAs and C/D box sRNAs identified in the *S. acidocaldarius* sRNome and the L7Ae RIP-Seq analysis. The Excel file summarizes the sRNAs that were identified in the *S. acidocaldarius* sRNome libraries with more than 100 reads (sheet 1). Putative C/D box sRNAs are listed separately (sheet 2). Finally, 107 L7Ae-interacting RNAs are listed for the RIP-Seq analysis (sheet 3) that were identified by the use of a custom peak calling pipeline (*q* value = ≤0.1) and by filtering for the mean normalized read count of the L7Ae sRNA data set (at least 3,000 reads). Download TABLE S1, XLSX file, 0.6 MB. Copyright © 2017 Daume et al. 2017 Daume et al. This content is distributed under the terms of the Creative Commons Attribution 4.0 International license.

### Translational autoregulation of L7Ae.

A k-turn motif was also identified in the enriched mRNA of *saci_1520*, which encodes the L7Ae protein. The L7Ae sRNA data set displayed a high read abundance for the gene’s 5′ UTR (Fig. 1b). The k-turn is formed by the identified Kt-n and Kt-b sequences within this 5′ UTR. The Kt-b sequence is preceded by a Shine-Dalgarno (SD) sequence. Thus, we hypothesized that L7Ae regulates its own translation by binding to the k-turn formed within its 5′ UTR, which masks the SD sequence and results in reduced translation of the *l7ae* mRNA.

**FIG 2:**
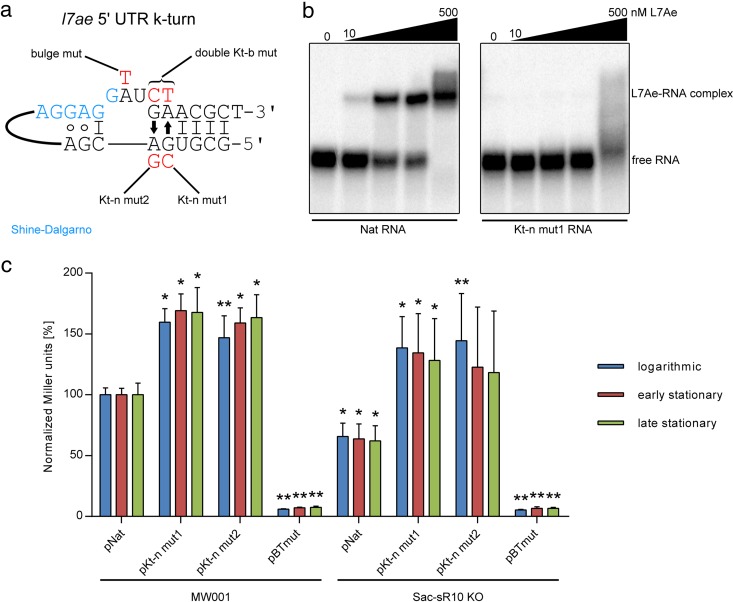
L7Ae binds its own 5′ UTR. (a) The schematic structure of the *l7ae* 5′ UTR k-turn is illustrated. A 3-nt bulge (GAU) is flanked at the 3′ side by two *trans* sugar-Hoogsteen (arrows) G · A pairs and a 4-bp stem. A single G-C base pair is present at its 5′ side. Mutants produced in this work are marked in red. mut, mutant. (b) EMSA data represent the binding of the *l7ae* 5′ UTR by recombinant L7Ae. Binding of the native 5′ UTR (Nat RNA) was observed with 10 nM L7Ae and with increasing concentrations (50, 100, and 500 nM L7Ae). The Kt-n strand-mutated 5′ UTR (Kt-n mut1) shows only unspecific binding at a high concentration of 500 nM. (c) Relative levels of β-galactosidase activity (normalized Miller units) are shown for *S. acidocaldarius* MW001 and Sac-sR10 KO cells that were transformed by the following plasmids: pNat (*l7ae* promoter plus native 5′ UTR), pKt-n mut1 (*l7ae* promoter plus Kt-n mutant 1), pKt-n mut2 (*l7ae* promoter plus Kt-n mutant 2), and pBTmut (BRE/TATA site mutated *l7ae* promoter plus native 5′ UTR). The assay was performed with strains during logarithmic growth (blue), early stationary growth (red), and late stationary growth (green). The values are normalized to those determined for MW001 plus pNat. Error bars indicate standard deviations of results from five biological replicates. Asterisks (*, Student’s *t* test; **, Welch’s *t* test) indicate the significance (*P* value= <0.05) of the data with respect to the MW001 strain or the Sac-sR10 KO plus pNat strain.

The schematic structure of the *l7ae* 5′ UTR reveals a simple, standard k-turn: a Watson-Crick base pair is followed by a three-nucleotide bulge and the two characteristic G · A pairs (Fig. 2a). The SD sequence flanks the bulge at the 5′ side. The formation of the k-turn was analyzed by mutating the nucleotides proposed to be involved in G · A pairing. Disruption of these non-Watson-Crick pairs was shown to be highly detrimental for proper k-turn folding and to prevent L7Ae binding (10, 23). Electrophoretic mobility shift assays (EMSAs) showed that recombinant L7Ae (Fig. S3) can efficiently bind the native 5′ UTR, while the mutation of a G · A pair resulted in binding deficiency (Fig. 2b).

10.1128/mBio.00730-17.5 FIG S3  Purification of recombinant His-Sumo-tagged L7Ae. SDS-PAGE analysis data show the purification steps of recombinant His-Sumo-tagged L7Ae. Lane 1 contains the pooled (p) fractions of the first nickel-NTA run. Lane 2 shows the results of overnight dialysis (d) of the protein after Sumo protease cleavage. Lane 3 displays the flowthrough (FT) of the second nickel-NTA run which contained the pure cleaved L7Ae protein. Download FIG S3, PDF file, 2.1 MB. Copyright © 2017 Daume et al. 2017 Daume et al. This content is distributed under the terms of the Creative Commons Attribution 4.0 International license.

Next, we performed reporter assays with *l7ae* promoter and 5′ UTR variants that were fused to the β-galactosidase gene to monitor the negative-feedback regulation of L7Ae. *S. acidocaldarius* MW001 strains that were transformed with mutants of the Kt-n sequence displayed a 1.6-fold increase in β-galactosidase activity in comparison to the native 5′ UTR (Fig. 2c). We postulate that these mutations impair k-turn formation and thereby abolish translational downregulation by L7Ae. The effects were observed during three different growth phases. Mutation of the BRE (B recognition element)/TATA sites led to a loss of β-galactosidase activity, suggesting that the *l7ae* promoter was disrupted. Next, we investigated augmentation of this negative-feedback loop by using an *S. acidocaldarius* strain that lacked the most abundant L7Ae interactor (Sac-sR10). Consequently, the amount of free L7Ae should have been increased within the cell. In agreement, a ΔSac-sR10 strain that was transformed with the native *l7ae* 5′ UTR displayed 1.6-fold-reduced β-galactosidase activity in comparison to MW001 wild-type (WT) strain transformants. The Kt-n sequence mutants showed 2.1-fold-higher enzyme activity in this background.

### Design of a bacterial GFP reporter system for the detection of L7Ae/k-turn interactions.

**FIG 3:**
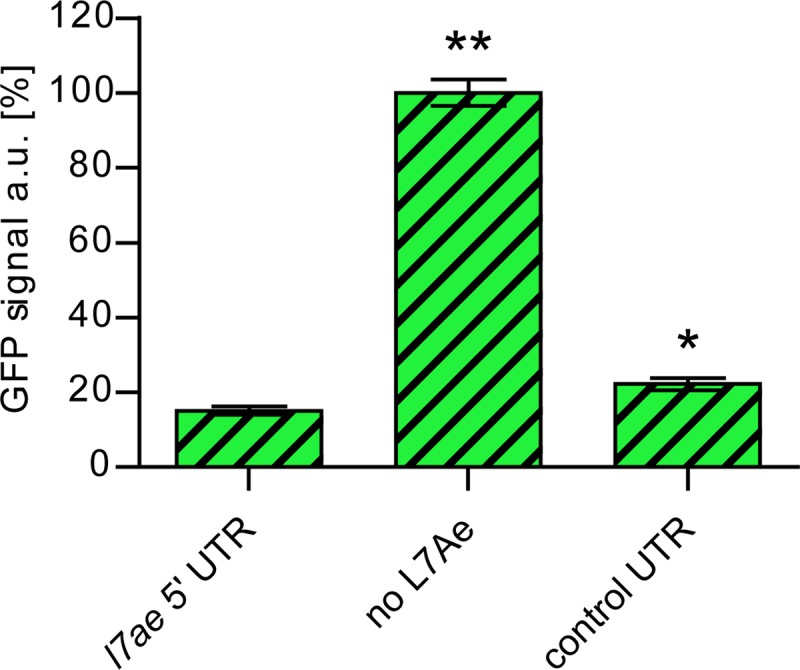
Toxicity effects of L7Ae overproduction in *E. coli*. The relative levels of GFP signals are shown for *E. coli* cells that were transformed by different variants of a plasmid which contained a constitutively expressed *sfgfp* gene and the following IPTG-inducible *l7ae* or region: the *l7ae* 5′ UTR upstream of the *sfgfp* gene, the frameshifted *l7ae* (no L7Ae), or the control UTR upstream of the *sfgfp* gene. Error bars indicate standard deviations of results from three biological replicates. Asterisks (*, Student’s *t* test; **, Welch’s *t* test) indicate the significance (*P* value = <0.05) of the data with respect to the strain containing the *l7ae* 5′ UTR. The GFP fluorescence was recorded from the GFP-positive population only.

Due to the high levels of L7Ae substrates (most notably C/D box sRNAs), the β-galactosidase reporter setup in *S. acidocaldarius* did not constitute an optimal system for testing further L7Ae/k-turn interactions. C/D box sRNAs are absent in *Bacteria*, and only weak interactions of endogenous L7Ae homologs YbxF and YlxQ with k-turns have been reported (24). Therefore, we designed a green fluorescent protein (GFP) reporter system for *Escherichia coli* to screen for motifs that are bound by L7Ae. *E. coli* Rosetta cells were transformed with a plasmid that contained an IPTG (isopropyl-β-d-thiogalactopyranoside)-inducible *l7ae* from *S. acidocaldarius* and a constitutively expressed superfolding GFP gene (*sfgfp*). The *l7ae* 5′ UTR was integrated upstream of the *sfgfp* gene to test for GFP downregulation by L7Ae, and fluorescence was quantified by flow cytometry. These cells showed a reduction in GFP signal of 85% compared to a strain without L7Ae (*l7ae* of the utilized plasmid was destroyed by introducing a frameshift) (Fig. 3). Exchanging the *l7ae* 5′ UTR by the use of a control UTR lacking a k-turn (LII-12 variant from the *E. coli* Pm promoter [25]) resulted in a slightly higher fluorescence signal; however, we still observed an unexpected strong (over 75%) reduction of the fluorescence strength. Therefore, we hypothesized that the overproduction of L7Ae could lead to toxicity effects in *E. coli*, possibly due to the unspecific binding of essential RNA molecules. To monitor this possibility, we followed the growth of the investigated bacterial strains.

**FIG 4:**
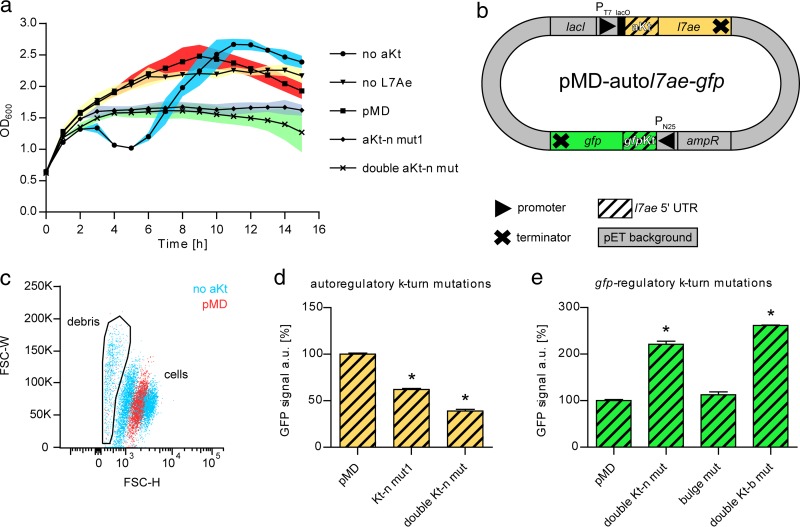
L7Ae toxicity can be cured by autoregulation. (a) The growth curves of IPTG-induced *E. coli* strains containing different variations of the pMD-auto*l7ae*-*gfp* plasmid are shown as no aKt (5′ UTR of pET plasmid), no L7Ae (frameshifted *l7ae*), pMD (pMD-auto*l7ae*-*gfp*), aKt-n mut1, and double aKt-n mut (see Fig. 2a). Three biological replicates were tested. Error bars (standard deviations) are depicted as color-filled areas. (b) The schematic structure of the pMD-auto*l7ae*-*gfp* plasmid is illustrated. The plasmid contains *l7ae* under the control of an IPTG-inducible T7 promoter (P_T7_), which is followed by the *l7ae* 5′ UTR (yellow streaked box). The presence of the k-turn formed by the 5′ UTR leads to the negative autoregulation of L7Ae translation (autoregulatory k-turn or aKt). The superfolding GFP gene (*gfp*) is expressed by the constitutive N25 promoter (P_N25_) from phage T5 (41). The *l7ae* 5′ UTR (green streaked box) is cloned upstream of the *gfp* gene and forms a GFP-regulatory k-turn (*gfp*Kt). (c) Data from flow cytometry analysis of *E. coli* cell populations that carry the pMD-auto*l7ae*-*gfp* plasmid (pMD) or the aKt-absent variant (no aKt) are illustrated in a dot plot. The pMD strain shows a single population, while the aKt-absent strain displays two cell populations and a larger amount of cell debris. FSC-W, forward-scatter width; FSC-H, forward-scatter height. (d) The relative levels of GFP signals of transformants that comprise mutations in the autoregulatory k-turn are shown. The utilized pMD-auto*l7ae*-*gfp* plasmids contain a control UTR upstream of the *gfp* gene to show toxicity-caused GFP downregulation in the aKt mutants. (e) The chart depicts the relative levels of GFP signals of transformants that comprise mutations in the *gfp*-regulatory k-turn. The values shown in panels d and e were normalized to the pMD strain values. Error bars indicate standard deviations of results from three biological replicates. Asterisks (*, Student’s *t* test) indicate the significance (*P* value = <0.05) of the data with respect to the pMD strain.

The strain expressing *l7ae* (no autoregulatory K-turn [no aKt]) showed a decrease of the cell density 3 h after induction but later displayed recovery of the cell growth, while the strain without L7Ae (frameshifted *l7ae*) grew normally (Fig. 4a). Growth recovery of the strain expressing *l7ae* was found to be mediated by escape mutants. The strains displayed normal growth when *l7ae* expression was not induced (Fig. S4). These growth patterns confirm that overproduction of L7Ae is detrimental to *E. coli* cells. Thus, we aimed to overcome this toxic effect by mimicking the negative-feedback-loop strategy that *S. acidocaldarius* employs to reduce L7Ae overproduction. To achieve this goal, an additional *l7ae* 5′ UTR was integrated upstream of *l7ae* to enable autoregulation of its expression. The resulting pMD-auto*l7ae*-*gfp* plasmid comprised an autoregulatory k-turn (aKt) and a *gfp*-regulatory k-turn (*gfp*Kt) sequence (Fig. 4b). Cells that were transformed with the plasmid displayed normal growth behavior (Fig. 4a). Flow cytometry confirmed the toxicity of the nonautoregulated L7Ae. Two cell populations and a large amount of debris could be observed for the strain without aKt (Fig. 4c). A significant portion of the cells was GFP negative, likely representing dead cells. The strain with an autoregulated *l7ae* (pMD) displayed only a single population. Mutations of the aKt, introduced to abolish autoregulation, led to growth defects, which were less drastic than for the strain without aKt (Fig. 4a). This restored toxicity also manifested in the GFP signal. The fluorescence of the aKt mutants was reduced in comparison to a strain with functional aKt (Fig. 4d). An aKt double mutant showed a more drastic effect. Next, mutational analysis of *gfp*Kt was performed. A 2.2-fold-higher GFP signal was measured for the double Kt-n mutant (Fig. 4e), whereas the bulge mutant, which should not have been affected in k-turn formation, showed a reduced GFP signal similar to that seen with the functional *gfp*Kt (pMD). A mutant with double mutations of the k-turn critical GA nucleotides within the Kt-b sequence again showed 2.5-fold-higher GFP fluorescence. These results demonstrate that the developed plasmid-borne autoregulatory GFP reporter system constitutes a reliable method to test L7Ae/k-turn interaction.

10.1128/mBio.00730-17.6 FIG S4  Growth curves of *E. coli* strains without the induction of IPTG. The growth curves of noninduced *E. coli* strains containing different variations of the pMD-auto*l7ae*-*gfp* plasmid are shown as follows: no aKt (5′ UTR of pET plasmid), no L7Ae (frameshifted *l7ae*), pMD (pMD-auto*l7ae*-*gfp*), aKt-n mut1, and double aKt-n mut (see Fig. 2a). Three biological replicates were tested. Error bars (standard deviations) are depicted as color-filled areas. Download FIG S4, PDF file, 0.3 MB. Copyright © 2017 Daume et al. 2017 Daume et al. This content is distributed under the terms of the Creative Commons Attribution 4.0 International license.

### The L7Ae negative-feedback loop is a conserved feature in *Archaea*.

**FIG 5:**
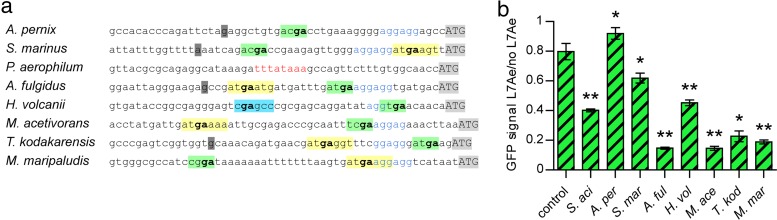
L7Ae binds the *l7ae* 5′ UTRs of various archaea. (a) The ~50-bp *l7ae* upstream sequences of eight taxonomically diverse archaea were extracted from the Clustal Omega alignment (Text S1). The proposed transcriptional start sites (dark gray), Shine-Dalgarno sequences (blue), and start codons (light gray) are marked. Potential Kt-b and Kt-n strands are highlighted in yellow and green, respectively. The GA nucleotides critical for k-turn formation are shown in bold. The sequence of *H. volcanii* comprises a potential Kt-b strand (highlighted in light blue) in a location apart from a Kt-b strand that was identified further upstream in the alignment (Text S1). The upstream sequence of the *P. aerophilum l7ae* does not show any of the marked features but comprises a TATA box (red) −25 bp upstream of the start codon. (b) The relative levels of GFP signals of *E. coli* transformants that comprise a control UTR or the *l7ae* upstream sequences described for panel a in place of the *gfp*-regulatory k-turn (except *S. acidocaldarius*) of the pMD-auto*l7ae*-*gfp* plasmid are shown. The GFP signals represent the ratio of L7Ae-producing cells to the cells producing no L7Ae (frameshifted *l7ae*). Error bars indicate standard deviations of results from three biological replicates. Asterisks (*, Student’s *t* test; **, Welch’s *t* test) indicate the significance (*P* value = <0.05) of the data with respect to the control strain. *S. aci*, *Sulfolobus acidocaldarius*; *A. per*, *Aeropyrum pernix*; *S. mar*, *Staphylothermus marinus*; *A. ful*, *Archaeoglobus fulgidus*; *H. vol*, *Haloferax volcanii*; *M. ace*, *Methanosarcina acetivorans*; *T. kod*, *Thermococcus kodakaraensis*; *M. mar*, *Methanococcus maripaludis*.

The conservation of the *l7ae* 5′ UTR among 121 archaeal species was investigated by multiple-sequence alignment (see Text S1 in the supplemental material). All investigated archaea, with the exception of *Thermoproteales* and *Nanoarchaeum equitans*, contained an SD sequence ~7 to 10 bp upstream of the *l7ae* coding sequence, indicating that almost all archaeal *l7ae* mRNAs comprise a 5′ UTR. This observation is striking in light of the general overabundance of leaderless transcripts in *Archaea* (26, 27). Potential transcriptional start sites could be assigned for most of the sequences due to the presence of conserved TATA boxes which revealed 5′ UTR sizes from 10 nt to 200 nt. High sequence conservation of the leader sequences was identified only for *Sulfolobales*. Bioinformatic prediction of k-turn formation within the 5′ UTRs is problematic due to the high sequence variability of k-turn/k-loop structures. However, we were able to identify possible conserved Kt-b and Kt-n strands within the 5′ UTRs by careful manual inspection. In order to investigate binding of the *S. acidocaldarius* L7Ae to other archaeal 5′ UTRs that were identified by this approach, the *gfp*Kt of pMD-auto*l7ae*-*gfp* was exchanged with 50 bp of *l7ae* upstream regions of eight archaeal model organisms spread across the archaeal domain (Fig. 5a). The *l7ae* leader sequences of *Staphylothermus marinus*, *Archaeoglobus fulgidus*, *Haloferax volcanii*, *Methanosarcina acetivorans*, *Thermococcus kodakaraensis*, and *Methanococcus maripaludis* comprised Kt-b and Kt-n strands, while only a Kt-n sequence could be identified for *Aeropyrum pernix*. *Pyrobaculum aerophilum* belongs to the order *Thermoproteales*, which did not comprise an *l7ae* 5′ UTR. Control UTR strains without *gfp*Kt showed around 80% GFP fluorescence compared to strains with nonfunctional L7Ae (Fig. 5b), which might account for the residual toxicity of L7Ae. For comparison, strains with the *gfp*Kt (*S. acidocaldarius l7ae* 5′ UTR) showed around 40% GFP fluorescence. The *A. pernix* UTR showed no L7Ae downregulation, probably due to the absence of the Kt-b strand. However, downregulation was observed for all other tested archaeal 5′ UTRs, particularly for the *A. fulgidus*, *M. acetivorans*, and *M. maripaludis* UTRs. Strains with the *P. aerophilum* 5′ UTR of *l7ae* displayed GFP signals with levels close to that measured for the background fluorescence and were therefore excluded. The results highlight that *S. acidocaldarius* L7Ae can bind to the *l7ae* 5′ UTR regions of various archaea, suggesting that the negative-feedback loop of L7Ae is a conserved feature and operative in most archaeal organisms.

10.1128/mBio.00730-17.1 TEXT S1  Multiple-sequence alignment of 121 archaeal *l7ae* promoter sequences. Putative TATA box elements, transcription start sites (TSS), Shine-Dalgarno (SD) sequences, and the Kt-n and Kt-b sequences are indicated. Download TEXT S1, DOCX file, 0.1 MB. Copyright © 2017 Daume et al. 2017 Daume et al. This content is distributed under the terms of the Creative Commons Attribution 4.0 International license.

### L7Ae binds to k-turn motifs in different mRNAs and SRP RNA.

**FIG 6:**
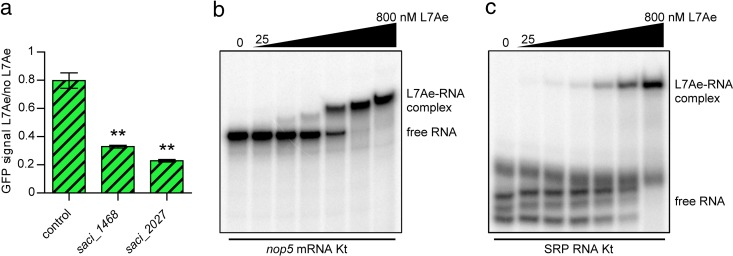
K-turn motifs identified in mRNA and SRP RNA are bound by L7Ae. (a) The relative levels of GFP signals of *E. coli* transformants which contain a control UTR in place of the *gfp*-regulatory k-turn are depicted. The *saci_1468* and *saci_2027* strains further contained the respective k-turn mRNA regions identified in the L7Ae RIP-Seq analysis directly downstream of the start codon (GFP fusion) for investigation of the translational regulation of mRNAs by L7Ae (Table S1). The GFP signals represent the ratio of the L7Ae-producing cells to the cells producing no L7Ae (frameshifted *l7ae*). Error bars indicate standard deviations of results from three biological replicates. Asterisks (**, Welch’s *t* test) indicate the significance (*P* value = <0.05) of the data with respect to the control strain. (b) EMSA data demonstrate the binding of L7Ae to the *nop5* mRNA Kt. The substrate comprises the first 125 nt of the mRNA, which constitutes the sequence that was found enriched in the L7Ae RIP-Seq analysis (Table S1). Full binding was observed at a concentration of 400 nM L7Ae (L7Ae gradient, 25, 50, 100, 200, 400, and 800 nM). (c) L7Ae shows binding to the k-turn within the SRP RNA of *S. acidocaldarius*. The substrate consists of the k-turn forming nucleotides 103 to 117 and nucleotides 245 to 259 that were linked by a 4-nt GNAR loop (Text S2). Three secondary structures were formed by the free substrate that showed full binding at an L7Ae concentration of 800 nM (L7Ae gradient, 25, 50, 100, 200, 400, and 800 nM).

RIP-Seq analyses revealed additional putative k-turn motifs in *S. acidocaldarius* mRNAs, which suggested that L7Ae might also be involved in regulating the translation of other transcripts. The proposed k-turn sequences of the *saci_1468* and *saci_2027* mRNAs were fused to the coding sequence of the *sfgfp* gene and tested in the established reporter system (Fig. 6a). The *saci_1468* and *saci_2027* mRNAs encode a DNA binding protein (hypothetical) and a glycosyltransferase (hypothetical), respectively. Both strains displayed downregulation of the GFP signal upon L7Ae induction. The proposed k-turn sequence of the *saci_1347* mRNA was tested via EMSA, which revealed L7Ae binding with affinity similar to that seen with the *l7ae* 5′ UTR (Fig. 6b). Interestingly, the *saci_1347* gene encodes the C/D box sRNP protein Nop5. In *S. acidocaldarius*, *saci_1347* is the first gene of an operon and the second, cotranscribed *saci_1346* gene encodes the C/D box sRNP protein fibrillarin. Thus, all three protein components of the C/D box sRNP could be regulated by L7Ae. A different potential L7Ae substrate is SRP RNA, as high read coverage was observed in the RIP-Seq data (Fig. S5a). In similarity to the *Sulfolobus solfataricus* results, we identified a putative k-turn in close proximity to the 5e motif of helix 5 of the *S. acidocaldarius* SRP RNA (Fig. S5b) (18). EMSAs verified that this k-turn was also bound by L7Ae (Fig. 6c).

10.1128/mBio.00730-17.7 FIG S5  K-turn formation within the *S. acidocaldarius* SRP RNA. (a) The coverage plots for the SRP RNA locus (*saci_0095*) of the sRNome, L7Ae sRNA, and frag RNA data sets are illustrated. High read numbers were present for the two L7Ae RIP-Seq data sets. The two regions that formed a potential k-turn in the SRP RNA are highlighted in gray. Each RNA profile contains one million mapped reads. (b) The two RNA strands from nucleotides 103 to 117 and nucleotides 245 to 259 (indicated with gray highlighting in panel a) of the SRP RNA form a potential k-turn (boxed). The sequence shown displays the substrate that was used for the EMSA (Fig. 6c). The two RNA strands were linked by a 4-nt loop. Download FIG S5, PDF file, 0.4 MB. Copyright © 2017 Daume et al. 2017 Daume et al. This content is distributed under the terms of the Creative Commons Attribution 4.0 International license.

10.1128/mBio.00730-17.2 TEXT S2  Oligonucleotide sequences used in this study. Oligonucleotides for the cloning of L7Ae constructs and substrates utilized in *in vivo* and *in vitro* assays are indicated. Download TEXT S2, DOCX file, 0.03 MB. Copyright © 2017 Daume et al. 2017 Daume et al. This content is distributed under the terms of the Creative Commons Attribution 4.0 International license.

### L7Ae autoregulation in *S. acidocaldarius.*

**FIG 7:**
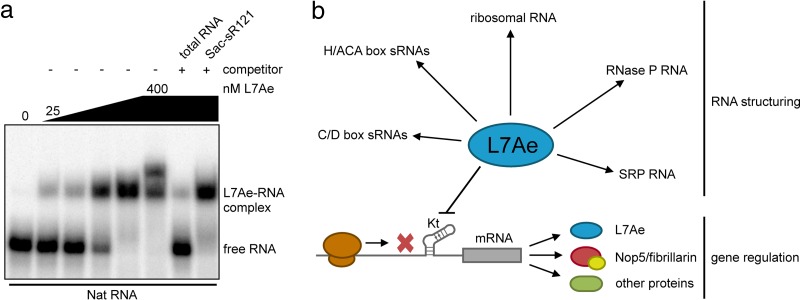
Significance of *l7ae* 5′ UTR binding. (a) The EMSA displays competition analysis of *l7ae* 5′ UTR (Nat RNA) binding. The Nat RNA is fully bound at a concentration of 400 nM L7Ae (L7Ae gradient, 25, 50, 100, 200, and 400 nM). A 10-fold concentration of unlabeled total RNA of *S. acidocaldarius* showed effective competition as free Nat RNA was obtained. The total RNA sample was depleted of small RNAs and comprised the 16S and 23S rRNAs as the main molecules (Fig. S1). Almost no competition of the Nat RNA binding was observed for a 100-fold excess of unlabeled C/D box sRNA Sac-sR121. (b) A model highlights the two roles of archaeal L7Ae. L7Ae binds to k-turns in various noncoding RNAs and induces a conformational change which recruits additional proteins. In addition, L7Ae represses the translation of its own mRNA and of other mRNAs by binding to a k-turn structure in the leader or the coding sequence.

We asked why the production of L7Ae is autoregulated in *S. acidocaldarius* and other archaea. The endogenous *l7ae* 5′ UTR of *S. acidocaldarius* was mutated, and the growth of the produced strains was monitored to screen for toxicity of L7Ae overproduction (Fig. S6). Strains with mutations of the Kt-n strand, which should abolish L7Ae autoregulation, showed a slight growth delay under optimal laboratory growth conditions. Our RIP-Seq data identified a plethora of L7Ae substrates that encompassed not only abundant C/D box sRNAs but also rRNAs, mRNAs, SRP RNAs, and other k-turn-containing RNA species. We investigated binding of the *l7ae* 5′ UTR in competition with an excess of total RNA and with C/D box sRNA Sac-sR121 (Fig. 7a). Total RNA efficiently outcompeted the *l7ae* 5′ UTR binding, while Sac-sR121 did not affect *l7ae* 5′ UTR binding significantly. Ribosomal RNAs, which represent L7Ae substrates, made up most of our total RNA purification (Fig. S1). We envision that the amount of L7Ae in the cell needs to be dynamically regulated to ensure proper coverage of all these different RNA substrates. This is especially true for volatile RNA molecules in hyperthermophilic archaeal species.

10.1128/mBio.00730-17.8 FIG S6  Growth curves of *l7ae* 5′ UTR variants in the *S. acidocaldarius* genome. The growth behavior of *S. acidocaldarius* strains that comprised genomic mutations in the *l7ae* 5′ UTR is depicted as follows: Nat (no mutation), aKt-n mut1, and double aKt-n mut (see Fig. 2a). The *l7ae* of all strains was genomically Flag-HA tagged, which simplified screening for the correct mutation. Three biological replicates were tested. Error bars (standard deviations) are depicted as color-filled areas. Download FIG S6, PDF file, 0.3 MB. Copyright © 2017 Daume et al. 2017 Daume et al. This content is distributed under the terms of the Creative Commons Attribution 4.0 International license.

## DISCUSSION

In this study, L7Ae from *S. acidocaldarius* was shown to autoregulate its production by binding to a k-turn structure formed within its mRNA leader. Additional k-turn substrates were identified in mRNAs. Thus, we propose a model which highlights two major roles of the archaeal L7Ae protein: (i) structuring of noncoding RNA and (ii) translational regulation via k-turn elements in mRNA sequences (Fig. 7b).

The L7Ae protein fulfills many important functions in the cell and binds k-turn elements in several essential noncoding RNAs, including ribosomal RNAs and RNase P RNA. It is plausible that these RNAs constitute preferred L7Ae substrates to allow proper ribosome and RNP complex assembly. We showed that the essential SRP RNA is a further binding partner of L7Ae, which is potentially recognized by a k-turn structure within helix 5 of the RNA. L7Ae binding of this k-turn has also been shown in *S. solfataricus* and could indicate that the SRP RNP complex in *Sulfolobales* is formed by the SRP RNA and the proteins SRP19, SRP54, and L7Ae (18).

It should be stressed that L7Ae is required to deal with changing substrate pools, namely, the C/D box sRNAs. Members of this RNA family constitute a large faction of L7Ae interactors, and the presence of highly variable C/D box sRNA genes in closely related archaeal organisms suggests their fast evolution (14). Recently, we showed that C/D box sRNAs are often cotranscribed with adjacent genes whose promoters are “hijacked” for C/D box sRNA production (28). This scenario can explain differential C/D box sRNA expression patterns in the *S. acidocaldarius* sRNome. In addition, C/D box sRNA abundance is coupled to the regulation of the “hijacked” promoters and environmental changes can have drastic effects on their production. The observed translational regulation of L7Ae and Nop5/fibrillarin allows the adjustments of the cellular levels of these C/D box sRNP core protein components in response to the availability of C/D box sRNA substrates. This gene regulation by translational control also constitutes a direct way to rapidly respond to environmental changes (29).

The finding of a k-turn motif in the bicistronic transcript of *nop5-fibrillarin* from *T. kodakarensis* provides further support for the idea that L7Ae regulation of Nop5 and fibrillarin is conserved among archaea (30). Jäger and coworkers also identified a k-turn in the transcript of *cbf5*, which encodes the catalytic subunit of H/ACA box sRNPs (30). Thus, L7Ae might also regulate the abundance of these sRNP complexes in other archaea.

Translational autoregulation of ribosomal proteins is often found in nature (31). We showed that L7Ae autoregulation is a conserved feature in *Archaea*. This autoregulation is also found in the eukaryotic domain, where the yeast homolog L30e regulates splicing and translation of its mRNA (32–35). The observation that the L7Ae homolog L30e of *S. acidocaldarius* binds to the endogenous L30e mRNA of *Saccharomyces cerevisiae* and regulates its translation *in vivo* underlines the preservation of this interaction (36). In contrast to archaea and eukaryotes, the bacterial L7Ae homologs YbxF/YlxQ were shown be dispensable and their k-turn interactions were reported to be weak (24, 37, 38). We hypothesize that autoregulation of the L7Ae/L30e family originated in *Archaea* and coincided with the advent of C/D box sRNAs. The members of *Thermoproteales*, which contain high numbers of C/D box sRNA genes, lack the *l7ae* 5′ UTR required for autoregulation, which suggests that other control mechanisms can exist in few archaeal species.

In this report, we present a bacterial reporter system in which we utilized L7Ae/k-turn interactions for the translational repression of GFP. This bacterial system resembles a synthetic switch designed for human cells (19). Here, we show that natural k-turn substrates are already present in archaeal mRNAs and can be utilized in bacteria in similar fashion. Our reporter system is provided on a single plasmid, is easily modifiable, and can be used to control translation of any gene of interest in *E. coli*. Tuning can be achieved by adjustment of the inducer concentration or by utilizing different archaeal k-turns, which showed diverse levels of repression efficiency. We envision that the design of archaeal k-turn feedback loops in synthetic circuits will allow studying the spatiotemporal regulation of gene expression.

### Conclusions.

RIP-Seq analysis of L7Ae identified known functional RNA interaction partners, novel noncoding RNA substrates, and also protein-coding mRNA molecules. The discovery of these substrates suggests that archaea can utilize k-turn motifs to facilitate translational regulation. A conserved k-turn element was found in the untranslated region of the *l7ae* mRNA, and the autoregulation of L7Ae was shown. L7Ae/k-turn interactions can be utilized to modulate gene expression in *E. coli*.

## MATERIALS AND METHODS

### Strains, media, and growth conditions.

*Sulfolobus acidocaldarius* DSM 639 uracil-auxotrophic strain MW001 (Δ*pyrE*) was a kind gift of Sonja-Verena Albers (University of Freiburg, Freiburg, Germany). MW001 and all generated mutants and plasmid-containing strains were aerobically grown at 180 rpm, 75°C, and pH 3.5 in Brock medium (39), which was supplemented with 0.1% (wt/vol) tryptone or N-Z-Amine, 0.2% (wt/vol) sucrose, and 20 µg/ml uracil (if required). *Sulfolobus* plates and competent cells were prepared as described previously (40). Brock medium (50 ml) was adjusted to an optical density at 600 nm (OD_600_) of 0.05 with a preculture grown for 2 days, and the cell density was measured every 12 h for the analysis of the growth behavior of the strains produced. The incubation was performed in long-neck Erlenmeyer flasks to reduce evaporation.

*E. coli* Top10 (Thermo Fisher Scientific), Rosetta 2 (DE3) pLysS (Novagen), and ER1821 (New England Biolabs) strains were grown in LB media at 37°C and 200 rpm in the presence of appropriate antibiotics. IPTG was supplemented at a concentration of 1 mM for the expression of L7Ae. *E. coli* ER1821, which contains plasmid pM.EsaBC4I, was used for methylation of *S. acidocaldarius* shuttle vectors. The growth of *E. coli* Rosetta transformants was investigated by growing precultures to the logarithmic phase, adjusting the OD_600_ to 0.6, inducing one-half of the main culture by the use of 1 mM IPTG, and monitoring the optical density every hour.

### Isolation of *S. acidocaldarius* total and small RNAs.

MW001 cells were harvested during the logarithmic phase (OD_600_ = 0.4). A 15-ml cell pellet was lysed in a homogenizer, and small (<200-nt) RNAs were isolated using a mirVana miRNA isolation kit (Ambion). Furthermore, the total RNA fraction that was depleted of small RNAs was recovered by following the manufacturer’s instructions.

### Cloning of plasmids and *in vitro* transcription templates.

For the C-terminal Flag-HA tagging of *l7ae* (*saci_1520*) and the deletion of the *sac-sR10* C/D box sRNA gene (*saci_0259*) in *S. acidocaldarius*, the flanking regions of the intended mutations were amplified via PCR, joined by overlap PCR, and cloned into the pSVA406 shuttle vector (40). The pSVA406-*saci_1520*CFHA plasmid was furthermore mutated by site-directed mutagenesis for the generation of the genomic 5′ UTR variants.

The *lacS* gene from *S. solfataricus* was amplified by primers that contained the native or mutated *l7ae* promoter region and 5′ UTR and was cloned into the pSVA1431 shuttle vector without the maltose-inducible promoter for the β-galactosidase reporter assays.

The pEC-A-Hi-Sumo plasmid was used for the production of recombinant L7Ae in *E. coli* and was a kind gift from Elena Conti (MPI Martinsried, Germany). The *l7ae* was amplified from *S. acidocaldarius* genomic DNA and cloned via ligation-independent cloning (LIC) into this pET plasmid-based vector, resulting in an N-terminal 6×His-Sumo-tagged gene.

The pEC-A-Hi-Sumo-*l7ae* plasmid was modified in several steps to obtain the pMD-auto*l7ae*-*gfp* plasmid. First, the His-Sumo tag was removed by inverse PCR. Next, the *sfgfp* gene with an *l7ae* 5′ UTR (*gfp*Kt) was amplified by PCR from the pASK-IBA3plus-*sfgfp* plasmid, which was a kind gift of Regine Kahmann (MPI Marburg, Marburg, Germany), and cloned into the vector via Gibson assembly. Then, the pN25 promoter from phage T5 was inserted upstream of the *sfgfp* gene by inverse PCR (41). Eventually, the final pMD-auto*l7ae*-*gfp* plasmid was obtained by introducing the *l7ae* 5′ UTR (aKt) upstream of *l7ae* by inverse PCR. Variations of the plasmid (frameshifted *l7ae* and aKt and *gfp*Kt mutants) were generated by site-directed mutagenesis. The control UTR (LII-12 UTR sequence of the *E. coli* Pm promoter [25]) and the 50-bp archaeal *l7ae* upstream regions shown in Fig. 5a were cloned upstream of the *sfgfp* gene by inverse PCR. The k-turn comprising sequences of *saci_1468* and *saci_2027* that were identified in the L7Ae RIP-Seq data were likewise inserted into the control UTR-containing plasmid as a fusion to the *sfgfp* gene.

Two complementary oligonucleotides were hybridized for *in vitro* transcription of the Nat, Kt-n mut1, Sac-sR121, SRP RNA Kt, and *nop5* mRNA Kt RNAs. The regions were then PCR amplified using a T7 promoter-containing primer, resulting in the runoff template for *in vitro* transcription. The hybridization oligonucleotides of the Nat, Kt-n mut1, and Sac-sR121 RNAs were cloned into the pUC19 vector prior to PCR amplification. The oligonucleotides used for cloning are listed in Text S2 in the supplemental material.

### Transformation of *S. acidocaldarius.*

The methylated shuttle vectors were transformed into competent *S. acidocaldarius* cells via electroporation (1,500 V, 600 Ω, and 25 µF) using a Gene Pulser electroporation system (BioRad). The cells were regenerated for 30 min at 75°C in recovery solution (Brock medium with 0.1% [wt/vol] N-Z-Amine or tryptone with 0.2% [wt/vol] sucrose) before plating on first-selection plates was performed. The plates were sealed to prevent evaporation and incubated for 7 days at 75°C.

### Generation of genomic tags and mutations in *S. acidocaldarius.*

Genomic C-terminal Flag-HA tagging of *l7ae*, mutation of the endogenous *l7ae* 5′ UTR, and deletion of the *sac-sR10* C/D box sRNA gene were performed as described previously (40). Briefly, the respective plasmids were transformed into *S. acidocaldarius* MW001 cells and correct integration was verified via colony PCR. The transformants were incubated for 5 days on second-selection plates bearing 5-fluoroorotic acid (5-FOA), inducing the loss of the plasmid and yielding a 50% chance of the presence of the aimed-for mutation, which was screened for by colony PCR.

### Immunoprecipitation of L7Ae from *S. acidocaldarius* by Flag-HA tandem affinity purification.

A 3-liter culture of an *S. acidocaldarius* strain with Flag-HA-tagged L7Ae was harvested during the logarithmic phase (OD_600_ = 0.4), and the pellet was resuspended in solubilization buffer (100 mM MOPS [morpholinepropanesulfonic acid; pH 6.5], 300 mM NaCl, 10% glycerol; 5 ml/g cells). The cells were lysed three times at 25,000 lb/in^2^ using a French pressure cell (SLM Aminco) and then centrifuged for 20 min at 30,000 × *g*. Flag-HA-tagged L7Ae was purified from the supernatant using a Flag HA tandem affinity purification kit (Sigma-Aldrich, St. Louis, MO, USA) following the instructions of the manufacturer. The L7Ae protein was eluted using 8 M urea. All steps were performed at 4°C.

### Northern blot analysis.

RNA was separated by denaturing 8% Tris-borate-EDTA–PAGE (TBE-PAGE), transferred onto a positively charged nylon membrane using a semidry electrophoretic transfer system (BioRad), and immobilized by UV cross-linking. The membrane was incubated at 42°C for 30 min in prehybridization buffer (Ultrahyb-Oligo; Ambion). A radiolabeled DNA oligonucleotide against C/D box sRNA Sac-sR10 was hybridized overnight at 42°C (Text S2). Detection of radioactivity was carried out by the use of a phosphorimager after membrane washing (2× SSC [1× SSC is 0.15 M NaCl plus 0.015 M sodium citrate] with 0.1% SDS and 1× SSC with 0.1% SDS).

### ZnCl_2_ fragmentation and preparation of cDNA libraries for Illumina sequencing.

Prior to cDNA library preparation, one aliquot of the L7Ae coimmunoprecipitated RNAs was fragmented by ZnCl_2_, which allowed the sequencing of longer L7Ae-bound RNAs, e.g., mRNAs. To this end, 16 µl of the L7Ae-CFHA urea eluate was mixed with 1.8 µl of 10× ZnCl_2_ fragmentation buffer (100 mM Tris-HCl [pH 6.8], 100 mM ZnCl_2_), the reaction mixture was incubated for 150 s at 94°C, and the reaction was immediately stopped on ice by the addition of 2 µl 500 mM Na_2_−EDTA (pH 8.0). Both the fragmented and nonfragmented urea eluates were separated by denaturing 10% TBE-PAGE. The RNA was extracted from the gel by incubation in RNA elution buffer overnight at 4°C and ethanol (EtOH) precipitated in the presence of glycogen and eluted in 15 µl 20 mM Tris-HCl (pH 7.5). The fragmented RNA (frag RNA) sample was furthermore treated with polynucleotide kinase (PNK) to cure the 2′,3′-cyclic phosphate and 5′ OH termini that resulted from ZnCl_2_ fragmentation. For this purpose, the 15 µl of eluate was mixed with 6 µl of 5× dephosphorylation buffer (500 mM Tris-HCl [pH 6.5], 500 mM MgAc, 25 mM 2-mercaptoethanol) and 1 µl of T4 PNK (Ambion) in a total volume of 30 µl and incubated for 6 h at 37°C. Subsequently, 1 mM ATP and 1 µl T4 PNK (Ambion) were added and the reaction mixture was incubated for 1 h at 37°C, followed by EtOH precipitation.

The following cDNA libraries were prepared: (i) four sRNome libraries from two biological replicates of *S. acidocaldarius* small-RNA isolates, (ii) two L7Ae sRNAs (nonfragmented), (iii) two L7Ae frag RNA libraries (fragmented) from two biological replicates of L7Ae coimmunoprecipitated RNAs, and (iv) one WT control library (nonfragmented) from a control purification without tagged L7Ae (see Table S2 in the supplemental material). The NEBNext Multiplex Small RNA Library Prep Set (New England Biolabs) was used for cDNA library preparation, and sequencing was performed on an Illumina HiSeq 2500 platform at the Max-Planck Genome Centre (Cologne, Germany).

10.1128/mBio.00730-17.10 TABLE S2  RNA-Seq mapping report. The numbers of reads obtained for each sRNome and L7Ae RIP-Seq library are listed. Furthermore, the counts of mapped reads and specific matches to the *S. acidocaldarius* genome are indicated. Plots illustrate the read length distributions per library. Download TABLE S2, XLSX file, 2.8 MB. Copyright © 2017 Daume et al. 2017 Daume et al. This content is distributed under the terms of the Creative Commons Attribution 4.0 International license.

### Mapping of Illumina sequencing data and identification of L7Ae-interacting RNAs.

Mapping of the sequencing data was performed using CLC Genomics Workbench 9.5.3 (Qiagen, Germany). The sequencing data were processed by (i) removal of sequences of low quality (quality score limit, 0.05; maximum number of ambiguities, 2), (ii) trimming of adapter sequences, and (iii) filtering by length (15-nt cutoff). The trimmed sequences were mapped to the *S. acidocaldarius* reference genome (GenBank accession number CP000077) using default settings. The mapping reports are detailed in Table S2. Identification of the *S. acidocaldarius* sRNome was performed by screening the sRNome libraries (1,000,000 mapped reads per library) for RNAs covered by at least 100 reads. In order to identify bona fide genome-wide L7Ae interactions, a custom peak calling pipeline was applied to the mapped reads as described previously (42). Briefly, the pipeline first defined potential binding regions from the read data using Blockbuster (43). In the second step, the statistical significance of these regions in terms of their differential levels of abundance was assessed using DESeq2 by comparing two replicates of L7Ae RIP-Seq data (L7Ae sRNA) with a control data set of untagged L7Ae (44). The fragmented data set (L7Ae frag RNA) was omitted from the peak calling pipeline as fragmentation naturally resulted in broader mapping patterns. The input to DESeq2 consisted of count tables which contained the number of reads counted for each identified binding region in the two experiments as well as the control library. On the basis of these counts, DESeq2 calculated size factors for each library, which were then used to calculate the normalized read counts by dividing each read count by the corresponding library size factor. The normalized counts were subsequently used for comparisons between libraries, specifically for differential expression testing, and to calculate fold change and false-discovery-rate (*q*) values as described before (42). Resulting binding sites with a *q* value of ≤0.1 were further filtered using the mean normalized read count of the L7Ae sRNA data set (at least 3,000 reads) in order to remove false-positive hits due to low-abundance RNAs. Enriched RNAs of the sRNome and the L7Ae-RNA interactome are listed in Table S1. The RNA-Seq data sets are available at Gene Expression Omnibus (GSE94748).

### Purification of recombinant L7Ae.

A 1.5-liter culture of Rosetta cells plus pEC−A-Hi-Sumo-*l7ae* was harvested 3 h after induction. The cells were homogenized in lysis buffer (20 mM Tris-HCl [pH 8.0], 10% [vol/vol] glycerol, 50 mM NaCl, 10 mM 2-mercaptoethanol, 10 mM imidazole, 1.5 mg lysozyme/gram cells [5 ml/g cells]), sonicated, and cleared by centrifugation (20,000 × *g*, 4°C, 20 min). The His-Sumo-tagged L7Ae protein was purified via nickel-nitrilotriacetic acid (Ni-NTA) affinity chromatography (HisTrap HP; GE Healthcare) and eluted at an imidazole concentration of 50 to 450 mM (10 to 500 mM gradient) using a fast protein liquid chromatography (FPLC) Äkta system (GE Healthcare). Sumo protease (10 µg/ml) was added to the pooled protein fractions, and the solution was dialyzed overnight in cleavage buffer (20 mM Tris-HCl [pH 8.0], 10% [vol/vol] glycerol, 50 mM NaCl, 10 mM 2-mercaptoethanol, 10 mM imidazole). The L7Ae protein was again purified by Ni-NTA chromatography and was separated from the cleaved His-Sumo tag due to elution in the flowthrough fraction.

### RNA *in vitro* transcription and electrophoretic mobility shift assays.

RNA was synthesized by runoff *in vitro* transcription for 3 h at 37°C in the presence or absence of [α-^32^P]ATP (40 mM HEPES [pH 8.0], 22 mM MgCl_2_, 5 mM dithiothreitol [DTT], 1 mM spermidine, 4 mM nucleoside triphosphates [NTPs], 30 nM T7 polymerase) and was then gel purified. Labeled RNA substrates (15,000 cpm [~1 pmol]) were mixed with purified L7Ae in a volume of 10 µl EMSA binding buffer (20 mM Tris-HCl [pH 8.0], 50 mM NaCl, 10 mM 2-mercaptoethanol, 1 mM MgCl_2_). Nat RNA binding competition assays were performed by the addition of 10-fold total RNA or 100-fold C/D box sRNA Sac-sR121. The reaction mixtures were incubated for 10 min at 70°C and then separated by nondenaturing 10% TBE-PAGE. Detection of the radioactivity was carried out by the use of a phosphorimager.

### β-Galactosidase reporter assays in *S. acidocaldarius.*

MW001 and Sac-sR10 knockout (Sac-sR10KO) transformants were harvested at the logarithmic (OD_600_ = 0.5 to 0.6), early stationary (OD_600_ = 1.4 to 1.6), and late stationary (OD_600_ = 1.6) phases, respectively. The β-galactosidase enzyme activity was determined as described previously (40). Briefly, 2 ml of the cultures was pelleted and the cells were resuspended in Z-buffer (10 mM KCl, 1 mM MgSO_4_, 60 mM Na_2_HPO_4_, 40 mM NaH_2_PO_4_, pH 7.0), supplemented with 0.5% (vol/vol) Triton X-100 and 1 mM phenylmethylsulfonyl fluoride (PMSF), to an OD_600_ of 3.2. The cell suspension (20 μl) was mixed with 170 µl Z-buffer and 10 µl *o*-nitrophenyl-β-d-galactopyranoside (ONPG) solution (12 mg ONPG–1 ml Z-buffer) and incubated at 42°C. ONPG hydrolysis mediated by the β-galactosidase was measured at 410 nm for 3 h in 5-min intervals in a plate reader (Infinite M200 Pro; Tecan). Furthermore, the protein concentration of the cell suspension was measured by the Bradford protein quantitation method (45). The β-galactosidase activity was quantified by the following equation (46, 47):

$$\text{Miller unit value}=\frac{60,000\;\times \;\left(A_{410\;\left(t_{2}-t_{1}\right)}-\text{autolysis at}\;410\;{\text{nm}}_{\left(t_{2}-t_{1}\right)}\right)\times \;7}{\text{time}\left(\text{s}\right)\times \text{volume of sample}\;\left(\text{ml}\right)\times \text{concentration of protein}\;(\text{mg}/\text{ml})}$$

### Flow cytometry analysis and calculation of the GFP fluorescence intensity.

Rosetta transformants bearing variations of the pMD-auto*l7ae*-*gfp* plasmid were grown, adjusted to an OD_600_ of 0.6, and split into two, and one culture was induced with 1 mM ITPG. Both induced and noninduced cultures (2 ml each) were incubated in 24-well plates for 4 h. Subsequently, 1:300 dilutions were prepared in a 96-well plate with phosphate-buffered saline (PBS) in a total volume of 300 µl. Flow cytometry was performed using a BD LSRFortessa cell analyzer, and GFP fluorescence was excited by a laser at 488 nm. A total of 10,000 events were recorded using a 30-µl sample volume and a flow rate of 0.5 µl/s. Analysis of the flow cytometry data was performed using FlowJo 10.1 (FlowJo, LLC). The data were processed by gating of cell populations (i) to remove doublets, (ii) to remove cell debris, and (iii) to exclude GFP-negative cells (note that the data corresponding to exclusion of GFP-negative cells pertain only to the toxic strains described for Fig. 3 that did not comprise autoregulated L7Ae). The median value was used for the calculation of the GFP signal. All presented GFP ratios were calculated by dividing the GFP signal of induced cells by the signal of noninduced cells, which reduced the variations between independent experiments. Furthermore, the GFP ratios included the quotient of IPTG-induced L7Ae/no L7Ae (frameshifted *l7ae*) strains to analyze the levels of downregulation under similar conditions of growth and also allowed the comparison of levels of L7Ae-induced signal reduction between strains with different *sfgfp* expression strengths, e.g., *H. volcanii l7ae* 5′ UTR (median GFP value [GFP_Med_], 1,133 arbitrary units [a.u.]) versus *M. maripaludis l7ae* 5′ UTR (GFP_Med_, 36,354 a.u.).

### Statistics.

Two-tailed, unpaired Student’s *t* tests were applied to calculate the significance of the data using a *P* value of 0.05. A Welch corrected two-tailed, unpaired *t* test was applied in cases of unequal variances.
