# Supplementary figures and images for: RIP-Seq Suggests Translational Regulation by L7Ae in Archaea
Source: mBio. 2017 Aug 1;8(4):e00730-17. doi: 10.1128/mBio.00730-17 (PMC5539422; doi:10.1128/mBio.00730-17)

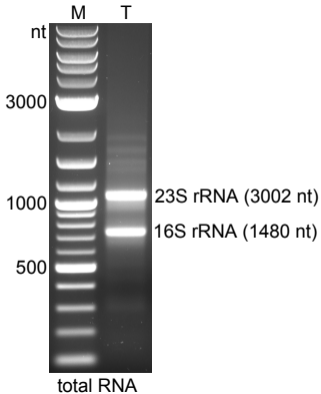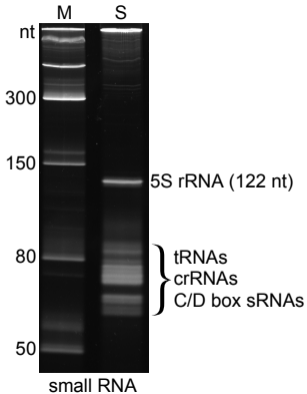

Supplement: FIG S1 [file mbo004173413sf1.pdf]

**a**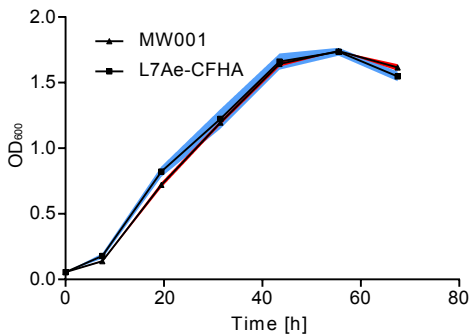**b**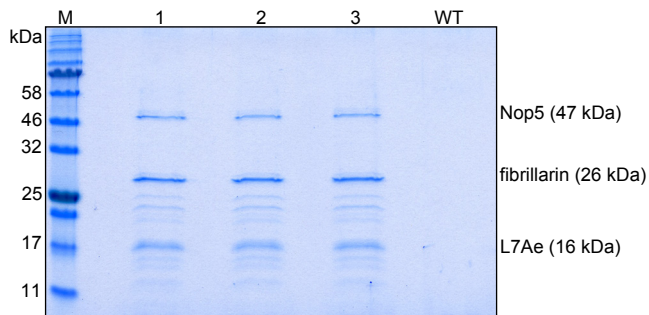**c**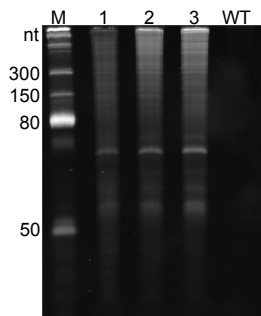**d**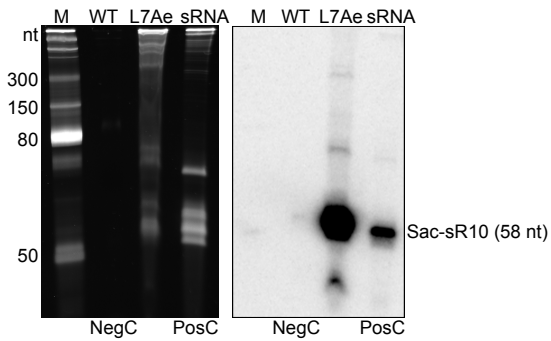

Supplement: FIG S2 [file mbo004173413sf2.pdf]

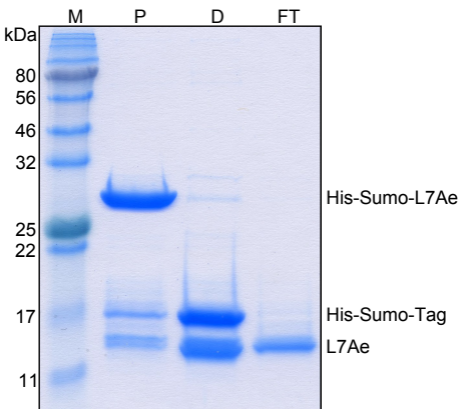

Supplement: FIG S3 [file mbo004173413sf3.pdf]

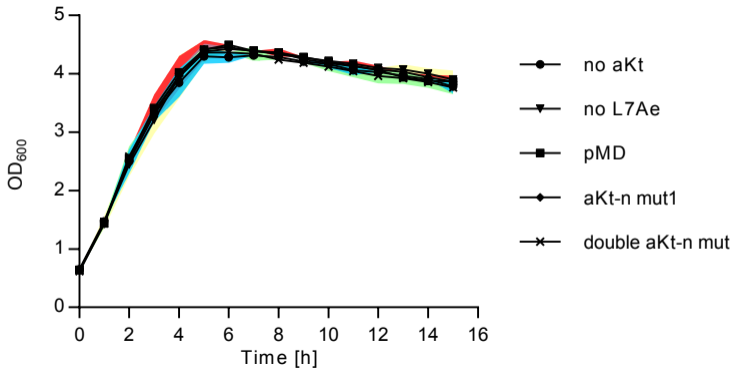

Supplement: FIG S4 [file mbo004173413sf4.pdf]

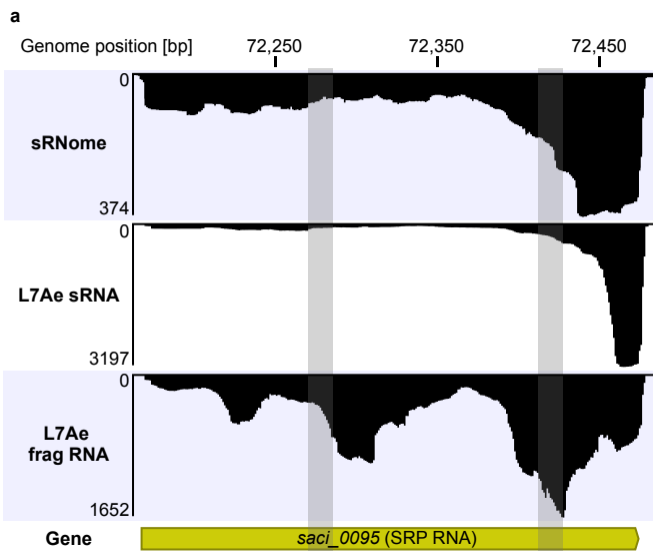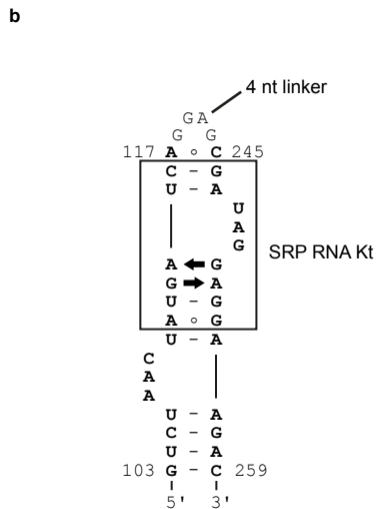

Supplement: FIG S5 [file mbo004173413sf5.pdf]

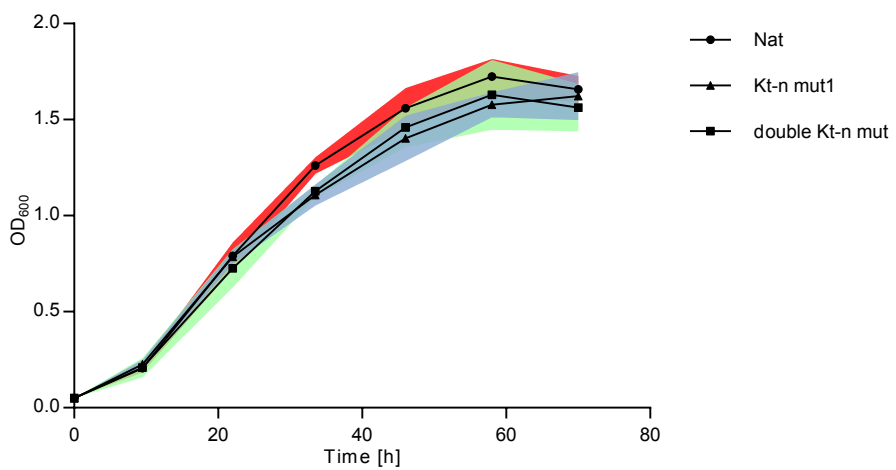

Supplement: FIG S6 [file mbo004173413sf6.pdf]
