# Supplementary material for: RIP-Seq Suggests Translational Regulation by L7Ae in Archaea
Source: mBio. 2017 Aug 1;8(4):e00730-17. doi: 10.1128/mBio.00730-17 (PMC5539422; doi:10.1128/mBio.00730-17)
Supplement: TEXT S1 [file mbo004173413s1.docx]

**Supplementary File 1.** Multiple sequence alignment of 121 archaeal *l7ae* promoter sequences

Crenarchaeota

TATA box TSS Kt-n SD Kt-b

Mcup_0156 ----------------------------------------------------------------------actgaggagcct--tctc--ttttgtcctttatttgtttttagtcttattattg--gttga-----------aaattttaaactggtttta--cga-aa-gaat--ttttgctaa-----tttgatatt-tcttttgaacttcccaatagagcacccgat-aagtaactttataatttataatacattcaatcaaattgcgtgacgacataagtaggaggatgaacgcca 200

Msed_2132 -------------------------------------------------------------------------gaggagatc--aggg--gctacggcgaggggtactttgagacc--ctaaag--ccagg-----------ggatattgtccagctggtt--cgc-ta-cgga--ttcgccagagtggacagcatatcacgtggtgagattaccatgatattcgcacat-gagtaacttt-ttaattctcgaggcttaaaatccacggcgtgacgacataggaaggaggatgaacgcaa 200

Ssol_1068 -------------------------------------------------tc------gttaatga-----atccctacc------aat--ga-ttcgatattatatctctcgtagaaaaaccaaaaggtgg-----------gaaattctcacatataaaa--ata-aa-ggtcttaaagatacttat-cttcaccttaaggtgctaagctttc---tgtcttatcctat-agtaaactttattttttgctaatacttctaaagacttgcgtgacgaaact--agaggaggATGAACGCG 209

SSO0091 -------------------------------------------------tc------gttaatga-----atccctacc------aat--ga-ttcgatattatatctctcgtagaaaaaccaaaaggtgg-----------gaaattctcacatataaaa--ata-aa-ggtcttaaagatacttat-cttcaccttaaggtgctaagctttc---tgtcttatcctat-agtaaactttattttttgctaatacttctaaagacttgcgtgacgaaact--agaggaggATGAACGCG 209

M1627_2112 ---------------------------------------------------------gctaggtg-----gttatcaac------att--tt-tcctaat--atata--acatcctttacacccc--aacg-----------tacttgttagataaggggg--tga-ta-ataaatgtaaatatctca--aatgaatgaaattgttgaccacac---tcccttattctat-agtaaactttattttttgctaatacttttaaagagttgcgtgacgaaact--agaggaggatgaacgcg 200

SiRe_1905 ------------------------------------------------------------ataca-----aatggcgac------act--a--tcggaac--atagaacaaaaactctcaatataaatata-----------gaaatttatatttatagac--tcc-aa-aacctggatgacacctat--ctccacctaaaagggctaagcttt---tgccttattctat-agtaaactttattttttgctactacttttaaagagttgcgtgacgaaact--agaggaggatgaacgcg 200

LD85_2297 ------------------------------------------------------------ataca-----aatgacgac------act--a--tcggaac--atagaacaaaaactctcaatataaatata-----------gaaatttatatttatagac--tcc-aa-aacctggatgacacctat--ctccacctaaaagggctaagcttt---tgctttattctat-agtaaactttattttttgctaatacttttaaagagttgcgtgacgaaact--agaggaggatgaacgcg 200

LS215_2197 ------------------------------------------------------------ataca-----aatgacgac------act--a--tcggaac--atagaacaaaaactctcaatataaatata-----------gaaatttatatttatagac--tcc-aa-aacctggatgacacctat--ctccacctaaaagggctaagcttt---tgctttattctat-agtaaactttattttttgctaatacttttaaagagttgcgtgacgaaact--agaggaggatgaacgcg 200

YG5714_2158 ------------------------------------------------------------ataca-----aatgacgac------act--a--tcggaac--atagaacaaaaactctcaatataaatata-----------gaaatttatatttatagac--tcc-aa-aacctggatgacacctat--ctccacctaaaagggctaagcttt---tgctttattctat-agtaaactttattttttgctaatacttttaaagagttgcgtgacgaaact--agaggaggatgaacgcg 200

YN1551_0761 ------------------------------------------------------------ataca-----aatgacgac------act--a--tcggaac--atagaacaaaaactctcaatataaatata-----------gaaatttatatttatagac--tcc-aa-aacctggatgacacctat--ctccacctaaaagggctaagcttt---tgctttattctat-agtaaactttattttttgctaatacttttaaagagttgcgtgacgaaact--agaggaggatgaacgcg 200

M1425_2032 ------------------------------------------------------------ataca-----aatgacgac------act--a--tcggaac--atagaacaaaaactctcaatataaatata-----------gaaatttatatttaaagac--tcc-aa-aacctggatgacacctat--ctccacctaaaagggctaagcttt---tgccttattctat-agtaaactttattttttgctaatacttttaaagagttgcgtgacgaaact--agaggaggatgaacgcg 200

M164_2039 ------------------------------------------------------------ataca-----aatgacgac------act--a--tcggaac--atagaacaaaaactctcaatataaatata-----------gaaatttatatttaaagac--tcc-aa-aacctggatgacacctat--ctccacctaaaagggctaagcttt---tgccttattctat-agtaaactttattttttgctaatacttttaaagagttgcgtgacgaaact--agaggaggatgaacgcg 200

Ahos_0594 ---------------------------------------------------------------tg-----ctttacaggaat--atta--a-------atgttaaatatcgtaaataattattt---ttaa-----------ttagatatgcattttaata--tta-aa-catgtgcataaaacaagttcagacattaatgattcaaaaccatctaacctaaacgttatc-aaatagctttttaagctaatgaatctcattcatttttgcgtgacgaaata--aggaggaggatgaacgt 200

Saci_1520 -----------------------------------------------aaac------cataaatg-----gagtaggagaaa--gcga--aattcgaaatttgaataaaggggagatagttcag---ttca-----------ttagatatggctt---------t-----------gtaaaggtcgatgagaaaagtgctgatgggcaagtaacagtcatattctcacat-gaataactttttaatggatagagac-aatctattttcgcgtgacgaaaaa--gaaggaggatgaacgct 200

ST1424 --------------------------------------------agccacc------tatatccg-----gttatggagaaa--aaga--aattgggaatctaaaaatcaatgaaatagtacag---ttca-----------taagatttgggtt---------c-----------gttagggtcgataataagatcgat---aataaggttaccgtaatattctcacat-gagtaactttattaacctaataagttaattcttttttgcgtgacgaaata--agaggag-gatgaacgc 200

ASAC_1012 ---------------------------------------------------------------------agatctaag------c-----tatcttgaagttaattaatataatcaaaacgaacatgttctccttccttttaatgacta----gaggcctt--cctttgaagacctcccgcaagttaagggccatggg-----ttactttcgactt-------attaaaggctagttattctttt--aaacccgtctcagcggtgaattcgtagcgacgctaggaggaggatgagggtct 200

Igag_0205 ------------------------------------------------------------------acgatgtttgggagaaa-gtgc--tttacaatcattagctgtaggagaaattatacagatggttaggcttggatttggaaaaa----------------------tagaatacat------cggaaaaaagcttgtg-gaaattatatatacacatgagtaatagtttcaaaatttcttaaatacca--gaacccagtaagaactattgaaaatataaaacgaggcgtaaaact 200

Tagg_1384 ---------------------------------------------------------attctgag----------------------------------cgagaaggagggctccataatacagttatataggattggtttcgcaaggatcgagaaggttt--tagaggacttggtggttgcggtttacgctcatgactagctcttccttctcgctaattaatccacaatatttataagacgc------gatag-cgttaaacccctagtatggacgaccaatagggaggaggatgaagt 200

Shell_1642 -----------------------------------------------------------cctcgc-----agaaa---------aggc--attaattaacgaagaacatggagacatagtacagttctatagaatagggtttgcaagaa----------------------ttgat-tcca--aagaaccagaggagatcaaatgtatttttgcacataaataacaaaaaccttataatatcatttactattattagcttttaagtcagacgaccgaagaattgggaggaggatgaagtt 200

Smar_0825 -----------------------------------------------------------cctcgc-----agaaa---------aagc--attaactaacgaagaacttggaaacatagtacaattctatagaatagggttcgcaagaa----------------------tagat-tcca--aagaaccagaggagatcaaatgtatttttgcacatgaataacaaaaaccttataataccatttactattatttggttttaaatcagacgaccgaagagttgggaggaggatgaagtt 200

DKAM_1353 ------------------------------------------ttagaatag------atactatcaaagaggaaaaagtaatagccgt--cttctcacacgaataagaccaccgga-------ttcattat----ta----------ta----------------------tggaaatgga--aagtcgcatcaaaagtagtttaaaaattattt-----aatgcccgtaatttataaagccctgacattattctatatagcttgtagacgaccacgagttaagggaggaggatgaacac 200

Desmu_1344 --------------------------------------------------------------------agagacagcgtcacagccgt--cttctcacatgactaaccatttgaccccgaacagtcattcc----aacctctatacccg----------------------ttcacaccca--gggtgcctcagccagtggtttgaaacacgttgccacgggagcaaatgatttataagag-cttacaccactcgatata-agcctgtagacgacctgagtaagggaggaggatgaatac 200

Tpen_0493 -------------------------------------------------------------------------cgaagtcaaccctgt--aaccatacctagacg-ctactgcaccaacgctagacaccca----c---------aca----ccccgactt--cat-cgaaaaccgttaagaagctttgcttcgcgcgttgcccgtttcgagacgcgtg-tcgagctctcgaaaaacaaaaaagtttaaatatgcagtagcagtgcgaatgcatgatgagcgagaggtgagcgaaaaATG 203

Igni_0230 ---------------------------------------------------------ctcaggct-----ccacagag------------gctgatgaggtagctccgggtgccgagaagaaggtgaggaa----ca---ccctacggggtccgaggcctc--gga-gcccgacctccaatcacctttcagcgct--------cag---cttgctcctc-atcggcgcc--gcgggcctcccttcactctttt--ttaacctccctccctcgtgcccctcacgacgaggggtagatgagg 200

APE_1818 ---------------------------------------------------------gtcatagc-----ccccctagtgta-taag---ggtttcaaagacgagcttgaagcccacgctgccagcatgac----c------gcaccgc---ccca-------gcg-tc-caggcgggatcaagaaagtttgaac--------catgtaggaggcctgg-ct-cgaacc--acattcttaattaggtctgccacacccagattctagaggctgtgacgacctgaaaggggaggaggagcc 200

Hbut_0543 -------------------------------------------------------------tacg-----cataggatttgc--gcgg--gttgatagcgtcgagacagacgaaaaggggaaagtaaggaa----g------gtagtgctcatctatgctc--atg-ac-tgagaag----aagctttcattcaa--------agtggggccgctagtt-ttgtaaacc---cttccaggcctagagccctaagcgtactatggaggggcgacgaccgagccccgggaggaggatgagcc 200

Pyrfu_0921 -------------------------------------------------------------tgat-----cttcgtatcagtacgtga--ggtcatacctgagagctttag-a------gaagggcatgag----g------atgaggctacgctgggctt--ctt-cc-taggattcataatgatgttgttact--------cgacttgacgctctcc-tagcgcagg--cgtaggtttcataaactcttgtctaacactccgaccgggcgacgacccggtcggggaggaggatgagcc 200

Cmaq_1846 ----------ag----------------tatctatgatgcaa----------------ttgatgagtta-gtgaacatggatgtgcttaataaagggaaggtgaat----gggagggagt--acattaccc--tta-aggctaatcttaatgatatagttaacatgttaacatcaatgaagaagca----ttaagtgagcttaagggtttcattaa-ct-gtgaaagtttttaaagggtgcagcaccgcatatggtgtATGAGTACAATAGACCCGAGGGTCTTTTACGCCAATCCACCT 242

Vdis_2417 --------cacgtacctaccag------tcacgatta--------------------gcccatggcaca-tcg-gccactacctgggc------gagttcgcaataacctcaaagatagtgcagcatggcg--agc-ctggtctcaaggctacgaggagca-cgctgcacatagcctca--aagtaat--ctcttcattacttcgtattgaaaaat-ac-ttataaatgccaacttcacggtttagacgagtATGGCAACAGTAAGTATAGACCCAAAGACATTTTACGCAAACCCACCG 248

VMUT_0759 --------tacctatctaccaa------ttacgatta--------------------gtccgtggcata-ttg-gtcattaccttggg------gagttcgcaataacttcaaagattgtgcagcatggcg--aac-caggtctcaaggcaacaaggagta-cattgcacatagcctca--aagtgat--acctccattatctcgtattgaaaaat-ac-ttataaatgccaatttcacggtatacacgagtATGGCAGCAGTAAGTATAGACCCAAAGACATTTTACGCAAATCCACCG 248

Pisl_0589 gctttatcccactatataccaccacatcatattctcactcccttgtcatcaaaagaagacaagcgattc-atatggcggatatagagacaaggctccgccgagagtctactcggtcttgtcccgttctacg--acagagtttatgaaattagattgg------agagtaaaaactcttattcaccaca--gagcaagatttgtagatgtaaATGGG-TG-TAAATACTTATATAGCTTTTGCTTTTTCTAAGGCCATGTCAGTGACAATAGACCCAAGAACGTTCTACGCAAATCCACCG 287

Pcal_0443 -----------------------------ggggattaggcag--gtggtca--ac--gtggaccccgtc-ttcttccagtacatggtc---ttcggcatagtcgtcatcttggtgctaat---gtttatgc--cgc-agggcgtcgtggcggagcgg------cctgtgaagacgctgaagcggagga--ggctcgaggagctccgcagagaggcg-gc-ctaaagtttatatattgggttatttctagggggccATGGCGGTGACGATTGACCCAAAGACGTTCTACGCCAACCCTCTG 245

Tneu_1580 -------------------------aggatagcctaagagcc--gtaataa--ac--gccgatccgaca-tttcttcagtacatcata---tttggtatcgtgattatcgtaatggtcat---gtttaggc--ctc-agggtctcgtgggagagagg------ccgtctaagacgttgagccgtcggg--ttttggagagggttaggggggagg-c-c----taaaatttatatagttgggtctttttcgaggccATGGCAGTGACGATTGACCCAAAGACGTTCTACGCAAATCCGCCG 245

PAE3347 --------------------------agaggggatacggcaa--attatta--at--gttgacccaata-tttttccagtatatcata---tttggtataataattattttagtacttat---gtttaggc--cac-aaggtataattagtgaaagg------cctgttaagactttaaaaagacaca--ttttagctaagttacgcgcagaggca-t----aaagatttataaagccagttctttgtggcaaccATGGCAGTGACGATAGACCCAAAGACGTTCTACGCAAATCCACCG 245

P186_1272 --------------------------agagggtattaggcag--attgtca--ac--gtcgatccagta-ttcttccagtacataata---ctaggcgttataataatacttgtgcttat---gtttaggc--ctc-agggcctcgttggtgagaag------ccggcaaagacattaaagaggcgtg--ttttagagaagttgcgcggggaggcg-t----aaatatttatatagccggttctttgtggcacccATGGCAGTGACGATAGACCCAAAGACGTTCTATGCAAATCCGCCG 245

Pars_1759 --------------------------agagggtattagacaa--gtagtaa--at--gtcgatccagtg-ttcttccagtatataata---tttggcataataataatactggtgcttat---gtatagac--ctc-agggcattataggcgagaag------ccagccaagaccttgaagaggtgga--ttctggaaaagttgcagagaggggcg-t----aaagatttataaaaccggttctttgtggcacccATGGCAGTGACGATAGATCCGAAGACCTTCTACGCGAACCCGCTG 245

Pogu_0372 --------------------------agagggtattagacaa--gtagtaa--at--gtcgatccagtg-ttcttccagtatataata---tttggcataataataatactggtgcttat---gtatagac--ctc-agggcattataggcgagaag------ccagccaagaccctgaagagacgga--ttctgacaaagttgcagagaggggcg-t----aaagatttataaaaccggttctttgtggcacccATGGCAGTGACGATAGATCCGAAGACCTTCTACGCGAACCCGCTG 245

TTX_1999 ----------------------------acggcatcaagcgg--atccact--atgaggtgttccacta-ctcttaaaaaa--tga-----ctattcctttcaattatattcagtctttc---tgagaggc--ccgcgagccggccgccgtaagcgc------tcgccctcgcgcgccagtagacgga--tgcaagacgccggctagatttgcctc-gt-aaaaatatttataacctactagattcccaagggccATGGCAACCACCGTAGACCCCAAGACATTTTACGCGAACTCAGTG 245

TUZN_1501 -----------------------------------------------------------------------------------tgc-----ttcgtcaacgcgacc-----aaatacggc---agagatcc--aagggatttggcttgggttagaaa------ggc-caggaa--------ggcccag--aaggccaagtaggaaagccgagccgc-tc-gaaaatatttatatccg-acctaatttgcaaggccatggcaaccaccgtagaccccaagacattttacgcgaactcggtg 182

start

Mcup_0156 ATGGCAAAACCATCTTATGTAAAATTTGATGTTCCGCCTGAATTGGCGGAGAAAGCTCTTGAAGCCCTTAAGAAAGCAAAAGAG---ACGGGAAAGATAAGAAAGGGTACAAACGAAGCTACAAAGGCTGTAGAGAGAGGAAATGCAAAGTTAGTAATCATAGCAGAAGATGTCCAACCTGAGGAAATAGTAGCCCATTTACCTCCATTATGCGAAGAGAAGAAAATATCCTACATTTACGTACCAACTAAGAAAGGAATTGGAGAAGCTTGCGGGCTACAGGTAGGTGCTGC---TGCG 494

Msed_2132 ATGGCTAAACCATCTTATGTAAAATTCGATGTTCCGCCAGAATTGGCGGAGAAAGCCCTAGAGGCTCTAAAGAAAGCTAAGGAA---ACAGGTAAGATTAGGAAGGGAACAAACGAAGCTACTAAGGCCATAGAGAGAGGGCAGGCTAAGCTTGTCCTCATTGCAGAGGACGTTCAACCTGAGGAAATAGTAGCACATCTACCACCTTTGTGTGAGGAGAAGAAGATACCATACATTTACGTTCCCACCAAGAAGGGCATAGGCGAAGCTTGCGGACTACAGGTAGGCGCGGC---AGCT 494

Ssol_1068 ATGTCAAAAGCTAGTTATGTTAAGTTTGAAGTACCACAAGACCTAGCAGATAAAGTATTAGAAGCAGTAAGAAAAGCTAAGGAA---AGCGGAAAGATAAAGAAGGGTACCAATGAGACAACAAAGGCTGTAGAAAGGGGTCAAGCTAAATTAGTAATTATTGCTGAAGATGTACAACCAGAGGAAATAGTTGCACACCTACCACTGTTATGCGATGAGAAAAAAATACCATATGTTTATGTTTCCTCTAAAAAAGCCTTAGGAGAAGCTTGTGGTTTACAAGTAGCCACAGC---ATCT 503

SSO0091 ATGTCAAAAGCTAGTTATGTTAAGTTTGAAGTACCACAAGACCTAGCAGATAAAGTATTAGAAGCAGTAAGAAAAGCTAAGGAA---AGCGGAAAGATAAAGAAGGGTACCAATGAGACAACAAAGGCTGTAGAAAGGGGTCAAGCTAAATTAGTAATTATTGCTGAAGATGTACAACCAGAGGAAATAGTTGCACACCTACCACTGTTATGCGATGAGAAAAAAATACCATATGTTTATGTTTCCTCTAAAAAAGCCTTAGGAGAAGCTTGTGGTTTACAAGTAGCCACAGC---ATCT 503

M1627_2112 ATGTCAAAAGCTAGTTATGTTAAGTTTGAAGTACCGCAAGATCTAGCAGATAAGGTATTGGAGGCAGTAAGAAAAGCTAAGGAA---AGTGGAAAAATAAAGAAAGGTACAAATGAGACAACAAAAGCAGTAGAAAGGGGACAAGCTAAATTAGTAGTTATTGCTGAAGATGTACAACCAGAGGAAATAGTTGCACACTTACCATTGCTATGCGATGAGAAAAAGATACCTTATGTATACGTTTCCTCTAAAAAAGCTTTAGGTGAAGCTTGTGGGTTGCAAGTGGCTACAGC---ATCT 494

SiRe_1905 ATGTCAAAAGCTAGTTATGTTAAGTTTGAAGTACCGCAAGATCTAGCAGATAAGGTATTGGAGGCAGTAAGAAAAGCTAAGGAA---AGTGGAAAAATAAAGAAAGGTACAAATGAGACAACAAAAGCAGTAGAAAGGGGACAAGCTAAATTAGTAGTTATTGCTGAAGATGTACAACCAGAGGAAATAGTTGCACACTTACCATTGCTATGCGATGAGAAAAAGATACCTTATGTGTACGTTTCCTCTAAAAAAGCTTTAGGTGAAGCTTGTGGGTTGCAAGTGGCTACAGC---ATCT 494

LD85_2297 ATGTCAAAAGCTAGTTATGTTAAGTTTGAAGTACCGCAAGATCTAGCAGATAAGGTATTGGAGGCAGTAAGAAAAGCTAAGGAA---AGTGGAAAAATAAAGAAAGGTACAAATGAGACAACAAAAGCAGTAGAAAGGGGACAAGCTAAATTAGTAGTTATTGCTGAAGATGTACAACCAGAGGAAATAGTTGCACACTTACCATTGCTATGCGATGAGAAAAAGATACCTTATGTGTACGTTTCCTCTAAAAAAGCTTTAGGTGAAGCTTGTGGGTTGCAAGTGGCTACAGC---ATCT 494

LS215_2197 ATGTCAAAAGCTAGTTATGTTAAGTTTGAAGTACCGCAAGATCTAGCAGATAAGGTATTGGAGGCAGTAAGAAAAGCTAAGGAA---AGTGGAAAAATAAAGAAAGGTACAAATGAGACAACAAAAGCAGTAGAAAGGGGACAAGCTAAATTAGTAGTTATTGCTGAAGATGTACAACCAGAGGAAATAGTTGCACACTTACCATTGCTATGCGATGAGAAAAAGATACCTTATGTGTACGTTTCCTCTAAAAAAGCTTTAGGTGAAGCTTGTGGGTTGCAAGTGGCTACAGC---ATCT 494

YG5714_2158 ATGTCAAAAGCTAGTTATGTTAAGTTTGAAGTACCGCAAGATCTAGCAGATAAGGTATTGGAGGCAGTAAGAAAAGCTAAGGAA---AGTGGAAAAATAAAGAAAGGTACAAATGAGACAACAAAAGCAGTAGAAAGGGGACAAGCTAAATTAGTAGTTATTGCTGAAGATGTACAACCAGAGGAAATAGTTGCACACTTACCATTGCTATGCGATGAGAAAAAGATACCTTATGTGTACGTTTCCTCTAAAAAAGCTTTAGGTGAAGCTTGTGGGTTGCAAGTGGCTACAGC---ATCT 494

YN1551_0761 ATGTCAAAAGCTAGTTATGTTAAGTTTGAAGTACCGCAAGATCTAGCAGATAAGGTATTGGAGGCAGTAAGAAAAGCTAAGGAA---AGTGGAAAAATAAAGAAAGGTACAAATGAGACAACAAAAGCAGTAGAAAGGGGACAAGCTAAATTAGTAGTTATTGCTGAAGATGTACAACCAGAGGAAATAGTTGCACACTTACCATTGCTATGCGATGAGAAAAAGATACCTTATGTGTACGTTTCCTCTAAAAAAGCTTTAGGTGAAGCTTGTGGGTTGCAAGTGGCTACAGC---ATCT 494

M1425_2032 ATGTCAAAAGCTAGTTATGTTAAGTTTGAAGTACCGCAAGATCTAGCAGATAAGGTATTGGAGGCAGTAAGAAAAGCTAAGGAA---AGTGGAAAAATAAAGAAAGGTACAAATGAGACAACAAAAGCAGTAGAAAGGGGACAAGCTAAATTAGTAGTTATTGCTGAAGATGTACAACCAGAGGAAATAGTTGCACACTTACCATTGCTATGCGATGAGAAAAAGATACCTTATGTATACGTTTCCTCTAAAAAAGCTTTAGGTGAAGCTTGTGGGTTGCAAGTGGCTACAGC---ATCT 494

M164_2039 ATGTCAAAAGCTAGTTATGTTAAGTTTGAAGTACCGCAAGATCTAGCAGATAAGGTATTGGAGGCAGTAAGAAAAGCTAAGGAA---AGTGGAAAAATAAAGAAAGGTACAAATGAGACAACAAAAGCAGTAGAAAGGGGACAAGCTAAATTAGTAGTTATTGCTGAAGATGTACAACCAGAGGAAATAGTTGCACACTTACCATTGCTATGCGATGAGAAAAAGATACCTTATGTATACGTTTCCTCTAAAAAAGCTTTAGGTGAAGCTTGTGGGTTGCAAGTGGCTACAGC---ATCT 494

Ahos_0594 ATGTCCAAACCATCTTATGTAAAATTTGAAGTTCCACAAGATTTAGTAGATAAAGCTCTAGAAGTTCTTAAGAAAGCTAAAGAA---TCTGGAAAGATAAAGAAAGGAACTAACGAGACTACAAAAGCAGTAGAAAGAGGCCAAGCAAAATTAGTATACATTGCAGAGGATGTACAACCAGAAGAGATAGTAGCTCATTTACCTGGATTGTGCGAAGAAAAGAAAGTTCCTTACATATACGTACCATCAAAGAAAGCTTTAGGCGAAGCATGCGGATTACAAGTTGCTGCAGC---TTCT 494

Saci_1520 GTGTCTAAACCCTCGTATGTAAAATTTGAAGTTCCACAAGAATTAGCAGATAAAGTATTAGAAGCAGTAAAGAAAGCAAAAGAT---TCAGGAAAGATAAAGAAAGGTACAAATGAAACTACAAAAGCTGTTGAAAGGAGCCAAGCCAAATTAGTGGTAATAGCTGAAGATGTTCAACCTGAAGAAATTGTAGCTCATTTACCATTATTGTGTGAAGAAAAGAAAATACCATATGTTTATGTACCTTCAAAGAAATCTCTAGGAGAAGCCTGTGGATTACAAGTTGCAGCAGC---TTCT 494

ST1424 ATGTCTAAACCCTCATATGTTAAATTCGATGTACCAGAGGATTTGGCTAATAAGGTATTAGATGCTGTAAGAAAAGCAAAGGAA---TCTGGAAAGATAAAGAAAGGAACTAATGAGACAACAAAAGCTGTAGAAAGAGGCCAAGCCAAGCTGGTTGTAATTGCAACAGACGTGCAGCCCGAAGAAATAGTAGCTCACTTACCACTATTATGCGAGGAAAAGAAAATACCATATGTTTATGTCCCGTCAAAGAAGGCATTGGGAGAAGCATGTGGCTTACAAGTGGCTGCTGC---CTCA 494

ASAC_1012 ATGTCTAAGCCCAGCTACGTGCTGTTTGAGGTGCCGGACGATCTAGCGGAGGACGTGTATAAGCTTGTAACTAAGGCAAGGGAA---ACTGGCAAGGTAAAGAAGGGCACTAACGAGACCACCAAGGCGGTAGAGCGTGGTACGGCAAAGCTAGTGGTGATAGCTACTGATGTTGATCCACCTGAGATAGTCGAGCACCTGCCGCTTCTATGTGACAGCAAGAAGATACCCTTCGTCTACGTCCCCAGCAAGAAGAGGCTTGGTGAGGCAGTCAAGATAGACGTGGCGGCCGC---CAGC 494

Igag_0205 ATGTCTAAACCATTTTATGTAAAGTTCGAGGTGCCCCCAGAAATAGCAGAGAAAGCATATCAGGCTCTTAAGAAAGCTAGAGAATCGGGAGGAAAAATAAGGAAGGGCACCAATGAGGTTACAAAGGGAGTGGAAAGAGGACTATGTAAAATAGTGTTTATAGCTGAGGATGTAGATCCACCAGAGGTAGTAGCACATCTACCTCTCTTATGCGAAGAAAAGAAGGTTCCATATCTATACGTGCCAAGTAAAAAGAGATTAGGAGAAGCAGCGGGTATTGAAGTAGCAGCAGC---ATCT 497

Tagg_1384 ATGCCTAAACCATTCTATGTTAAGTTCGAGGTACCGCCTGAGCTGGCGGAGAAGGTCTACGAGGCCGTGAAAAAAGTCAGGGAAACCGGTGGCAAGATCAAGAAGGGCACTAACGAGACCACGAAGGCTGTTGAGAGAGGGCAGGCAAGGCTTGTAGTAATAGCCGAGGATGTTGACCCACCGGAGATCGTTGCCCATCTGCCACTGCTGTGCGATGAGAAGAAAATACCCTACGTCTACGTTCCAAGCAAGCAAAAGCTTGGCGAGGCAGCAGGCATTGAGGTAGCGGCTTC---AAGC 497

Shell_1642 ATGTCTAAGCCGTTCTATGTAAAATTCGAGGTACCCTCAGAACTAGCTGAGAAAGTATATGAAGCAGTCAAGAAGGTTAGAGAGACAGGTGGTAAGATAAAGAAGGGAACAAATGAAACAACAAAAGCTGTTGAAAGAGGAATAGCTAGATTAGTAATAATTGCTGAAGACGTTGATCCACCAGAAATAGTTGCTCACCTACCTTTACTCTGCGATGAAAAGAAAATACCATATGTCTATGTCCCAAGCAAGAAGAAGCTGGGTGAAGCAGCAGGTATAGAGGTGGCTGCAGC---TAGC 497

Smar_0825 ATGTCTAAACCATTCTATGTAAAATTCGAAGTACCTCCAGAACTAGCTGAGAAAGTATATGAAGCAGTTAAAAAGGCTAGAGAGACAGGTGGTAAGATAAAGAAGGGAACAAATGAGACAACAAAAGCTGTTGAAAGAGGAATAGCAAAACTAGTGATAATTGCTGAAGACGTTGATCCACCAGAAATAGTTGCTCATCTACCTCTACTCTGCGACGAAAAGAAAATACCATATGTTTATGTCCCAAGCAAAAAGAGGCTAGGTGAAGCAGCGGGTATAGAGGTAGCTGCAGC---TAGC 497

DKAM_1353 ATGCCTAAGCCGTTTTATGTAAAGTTCGAGGTACCGCCTGAGCTAGCTGATAAAGTATACCAGGCAATATCAAAGGTAAGAGAGACTGGTGGGAAAATCAAGAAGGGCACAAATGAAACAACAAAGGCTGTTGAAAGAGGACAAGCTAAGCTGGTTGTCATAGCTGAAGATGTTGACCCACCTGAAATAGTTGCCCACCTACCACTATTATGCGATGAGAAGAAGATACCATATGTCTACGTGCCAAGCAAACAGAAACTGGGCCAAGCAGCCGGTATTGAGGTATCTGCTGC---CTCT 497

Desmu_1344 ATGCCTAAGCCGTTTTACGTAAAGTTCGAGGTACCTGCGGAACTAGCTGAGAAAGTATACCAGGCGGTAGCGAAAGTTAGGGAAACCGGTGGAAAAGTCAAGAAGGGAACAAACGAGACCACGAAAGCTGTTGAGAGGGGGCAGGCTAAGCTAGTCGTAATAGCTGAGGATGTGGAGCCCCCGGAGATAGTGGCACACCTACCTCTACTCTGCGAGGAGAAGAAGATACCATACGTTTACGTGCCGAGCAAGCAGAAGCTGGGTCAGGCAGCCGGCATAGAGGTGTCAGCTGC---CTCC 497

Tpen_0493 TCCAAGAAACCGTTTTACGTTAGGTTTGATGTCCCACCGGAACTGGCGGAAAAAGCCTACCAAGCTCTCAGCCTGGCAAGGGAGAAAGGCGGTAAGATAAAGAAGGGAACAAACGAGACCACTAAAGCTGTCGAGCGTGGACTTGCAAAGCTAGTTCTCATAGCCACTGATGTAGATCCTCCAGAGGTGGTGGCACACTTGCCGTTGCTCTGCGAGGAAAAGAAGGTTCCGTACGTCTACGTTCCGAGCAAAGAGCGCCTTGGAAAAGCCGCCGGTATAAACGTCGCAGCGGC---TGCT 500

Igni_0230 ATGAGCAAGCCGTTCTACGTTAGGTTTGAAGTGCCCCCCGAGTTGGCCGAGAAGGCTTACGAGGCTTTGAGGAAGGCCAGAGAGAGCGGCGGGAAGATAAAGAAGGGTACTAACGAAACTACCAAGGCCGTCGACAAGGGCCTCGCGAAGCTGGTGCTGATCGCGGAGGACGTGGACCCCCCAGAGATAGTGGCGCACTTGCCCTTGCTCTGTGAAGAGAAGAAGATACCTTACGTCTACGTGCCCAGCAAGAAGAAGCTCGGGGAGGCTGCCGGCATAGAGGTGCAAGCCGC---TGCC 497

APE_1818 ATGAGTAAGCCTATATATGTGAGGTTCGAGGTTCCGGAGGACCTAGCCGAGAAGGCTTATGAAGCCGTTAAGCGTGCAAGGGAG---ACGGGCAGGATAAAGAAGGGGACGAACGAGACTACAAAAGCCGTGGAGAGGGGTCTCGCCAAGCTAGTTGTGATAGCAGAGGACGTGGACCCGCCGGAGATAGTCATGCATCTGCCGCTTCTCTGCGACGAGAAGAAGATACCCTACGTCTATGTACCCAGCAAGAAGAGGTTAGGCGAAGCCGCCGGTATAGAAGTAGCAGCGGC---AAGC 494

Hbut_0543 ATGAGCAAGCCGTTCTATGTTAGGTTCGAGGTTCCACCAGAGCTAGCAGAGAAGGCCTACCAGGCACTAGAGATAGCAAGGAAG---ACTGGCAAGATAAAGAAGGGCACCAACGAGACCACTAAGTGTGTCGAGCGCGGCCTAGCAAAGCTAGTACTCATAGCTGAGGATGTAGACCCACCAGAGATCGTTGCTCACCTACCACTACTCTGCGAGGAGAAGAAGATACCCTACGTCTATGTACCGAGCAAGAAGAGGCTAGGCGAGGCTGCAGGCATAGAGGTTGCAGCAGC---TAGC 494

Pyrfu_0921 ATGAGCAAGCCGTTCTATGTTAGGTTCGAGGTCCCAGCCGAGCTAGCCGAAAAGGCATACAAGGCGCTCGAGATTGCAAGGAAG---ACGGGCAAGATCAAGAAGGGTACCAATGAGACCACAAAGTGCGTCGAGCGCGGTCTTGCCAAGCTCGTGCTAATAGCCGAGGATGTAGACCCGCCGGAGATCGTTGCTCACCTACCCCTACTATGCGAGGAGAAGAAGGTACCGTACGTCTACGTGCCCAGCAAGAAGAGGCTAGGCGAGGCTGCGGGCATCGAGGTGGCTGCGGC---TAGC 494

Cmaq_1846 CAGGGGAAGCCAATATACGTTAGGTTTGCTGTCCCCCAGGAGTTAGCTGAAATGACGTATAATGTGGTGAGCATGGCTAAGCAG---ACTGGGGTAGTTAAGAGGGGTTCCAATGAGACAACCAAGATGATTGAGAGGGGTTTAGCTAAACTAGTGGTTATCGCTGAGGATGTTGATCCACCGGAAATAGTAATGCACCTACCATTACTATGCGAGGAAAAGGGCATACCGTACATTTACGTACCCAGTAAGGAGAGGTTAGGTAAGGCAGTTGGCTTATCATCATACGCCTC---AGCA 536

Vdis_2417 CAGGGAAAACCATTCTACGTCAGATTCGCAGTGCCGCAGGAAATAGCCGAGAAGGCCTACGAAGTATTGTCGGCTGCCAGGGAG---ACGGGGAAGATAAAGAAGGGGACTAATGAGGTAACGAAGGCTGTGGAAAGAGGCTTGGCCAAGTTAGTCCTTATCGCGGAGGATGTCGACCCACCTGAGATCGTTGCCCACCTACCAATACTCTGCGAGGAGAAGGGCGTGCCTTACGTCTATGTGCCAAGCAAGGAGAGACTTGGCAAGGCCGCTGGGCTGCAGAACACGTCGGCAGCCTCA 545

VMUT_0759 CAGGGGAAGCCATTTTACGTAAGATTCACAGTACCGCAGGAAATAGCTGAGAAGGCTTATGAGGTTTTATCAGCTGCAAGGGAA---ACGGGCAGGATTAAGAAAGGCACCAATGAGGTTACTAAGGCTGTTGAAAGAGGACTAGCAAAGCTTGTTCTTATAGCAGAGGATGTTGATCCACCTGAGATTGTGGCTCACTTACCAATACTTTGTGAGGAGAAGGGTGTACCCTATATTTATGTGCCGAGTAAGGAGAGACTTGGCAAGGCTGCTGGTCTTCAGAGTACGTCAGCAGCATCA 545

Pisl_0589 CCGGGGAAGCCATTTTATGTACGATTTGAAGTTCCAGCCGAGGTTGCTGAGAAAGCGTTGGAAATACTGTCAATAGCTAGACAA---ACAGGGAAGATTAAGAAAGGGACTAATGAAACTACAAAGGCCGTGGAAAGAGGACTTGCGAAACTTGTACTTATTGCAGAAGACGTCGACCCGCCGGAAGTTGTGGCGCATTTGCCACTTCTTTGTGAAGAGAAGAAAGTTCCCTATGTCTATGTGCCATCTAAAGAGAAGTTGGGCAAAGCGGCGGGTATTAATGTATCTGCGGC---ATCA 581

Pcal_0443 CCGGGGAAGCCGTTCTACGTCAGATTCGAGGTGCCGAGCGACGTGGCTGAGAAGGCGCTGGAGATACTTTCAATTGCGAGGCAG---ACGGGGAAGATCAAAAAGGGGACTAACGAGACTACTAAAGCCGTGGAGCGCGGCCTTGCGAAACTTGTGTTAATTGCCGAGGACGTGGACCCCCCCGAGGTTGTTGCCCACCTCCCGCTTCTCTGCGAGGAGAAGAAGGTGCCCTACGTCTATGTGCCGTCTAAGGAGAAGTTGGGAAAGGCGGCTGGGATAAACGTGGCCGCCGC---GGCC 539

Tneu_1580 CCGGGTAAGCCATTCTATGTGAGGTTCGAGGTGCCGGCCGACGTGGCCGAGAAAGCTCTGGAGGTGCTTTCTGTGGCGAAGCAG---ACTGGCAAGATCAAGAAGGGGACAAACGAGGCTACTAAGGCGGTTGAGCGCGGTTTGGCGAAGCTCGTGCTTATAGCTGAGGACGTGGATCCGCCTGAGGTTGTGGCGCACCTCCCGCTTCTGTGCGAGGAGAAAAAGGTTCCCTACGTCTATGTGCCTTCAAAGGAGAAGTTGGGTAAGGCCGCCGGTATAAACGTCTCCGCAGC---CGCA 539

PAE3347 CCAGGAAAGCCCTTCTATGTGAGATTTGAAGTGCCCAATGATGTGGCGGAAAAGGCGCTGGAGATCTTGTCCATCGCTAGACAA---ACTGGCAAGATAAAGAAGGGCACTAATGAGACCACAAAAGCCGTCGAGAGGGGGCTGGCGAAATTAGTGCTAATCGCTGAAGATGTAGATCCTCCAGAGGTGGTGGCCCACCTCCCATTGCTGTGTGAAGAAAAGAAAGTGCCCTATGTCTACGTTCCGTCTAAGGAGAAACTGGGAAAAGCCGCCGGTATAAACGTGTCCGCAGC---TGCC 539

P186_1272 CCTGGGAAGCCTTTCTATGTGAGATTCGAGGTGCCTGGCGACGTGGCTGAAAAAGCCCTTGAGATACTGTCTATAGCGAAGCAG---ACTGGGAAGATTAAGAAGGGCACTAACGAGGCTACGAAGGCCGTCGAGAGGGGGTTGGCGAAGCTGGTGCTGATAGCTGAGGATGTGGATCCCCCGGAGGTGGTGGCTCACTTACCGCTGTTGTGTGAGGAGAAGAAGGTGCCTTACGTATATGTGCCTTCTAAGGAGAAGTTGGGGAAGGCCGCTGGCATCAACGTGTCTGCAGC---TGCG 539

Pars_1759 CCCGGTAAGCCATTTTATGTAAGGTTCGAAGTGCCAAGCGATGTAGCTGAGAAGGCCTTAGAGATACTATCTATTGCAAAGCAG---ACTGGGAAAATTAAGAAAGGCACCAACGAGGCTACTAAAGCAGTTGAGAGAGGGCTGGCTAAGCTTGTCCTAATAGCAGAGGATGTTGATCCTCCCGAGGTGGTTGCCCACCTGCCGTTGCTCTGCGAGGAGAAAAAGGTTCCATATGTCTATGTGCCTTCTAAGGAGAAGTTGGGCAAAGCCGCGGGCATAAACGTTTCTGCTGC---AGCG 539

Pogu_0372 CCCGGTAAGCCATTTTACGTAAGGTTCGAAGTGCCAAGCGATGTAGCTGAGAAGGCCTTAGAGATACTATCTATTGCAAAGCAG---ACTGGGAAAATTAAGAAAGGCACCAACGAGGCTACTAAAGCAGTTGAGAGGGGGCTGGCTAAGCTTGTCCTAATAGCAGAGGATGTTGATCCTCCCGAGGTGGTTGCCCACCTGCCGTTGCTCTGCGAGGAGAAAAAGGTTCCATACGTCTATGTGCCTTCTAAGGAGAAGTTGGGCAAGGCCGCAGGCATAAACGTCTCTGCTGC---AGCA 539

TTX_1999 CAGGGTAAGCCATTCTATGTAAGATTCGAGGTTCCAGCAGAGCTAGCCGAGAAGGCCTACGATATACTTAACGTCGCGAGGCAG---ACTGGCAAAATCAAGAAGGGCACTAACGAGGTCACAAAGGCTGTTGAGCGCGGCCTAGCTAGGCTGGTGCTCATCGCGGAGGACGTGGATCCGCCTGAAGTGGTGGCCCACTTGCCAATACTGTGCGAGGAGAAGAAGGTGCCCTACGTCTATGTGCCCTCCAAGGAGAAGTTGGGCAAAGCCGCCGGCATTAACGTTGCCGCAGC---CGCC 539

TUZN_1501 cagggtaagccattctacGTGAGGTTCGAGGTGCCCGCCGAGCTCGCAGAGAAGGCGTACGAGATCCTAAACACCGCGAGGCAG---ACAGGCAAGATAAAGAAAGGCACAAACGAGGTCACTAAAGCCGTCGAGAGAGGGCTGGCTAAGCTCGTGCTGATAGCCGAGGACGTCGACCCGCCGGAGGTCGTGGCGCACCTCCCAATTCTGTGCGAAGAGAAGAAGGTGCCCTATATCTACGTGCCGTCCAAGGAGAAGCTCGGCAAGGCTGCCGGCATAAATGTCTCGGCGGC---CGCC 476

Mcup_0156 GCTGCTATAACTGATCCAGGACAAGGAAAAGATATACTTGATGAGGTAATTAAGAGAGTGTCTGAACTAATCGGCAAATCCTAA--------------------------------------------------------------------------------------------- 578

Msed_2132 GCGGCCATATTGGATCCAGGACAGGGCAAGGACGTTTTAGACGAAGTAATAAAGAGAGTCTCGGAGCTTACAGGTAAGTCTTAA--------------------------------------------------------------------------------------------- 578

Ssol_1068 GCAGCTATCTTAGAGCCTGGCGAAGCTAAGGATCTAGTTGATGAAATAATTAAGAGAGTAAACGAGATTAAAGGTAAAACTTCAAGTTAA--------------------------------------------------------------------------------------- 593

SSO0091 GCAGCTATCTTAGAGCCTGGCGAAGCTAAGGATCTAGTTGATGAAATAATTAAGAGAGTAAACGAGATTAAAGGTAAAACTTCAAGTTAA--------------------------------------------------------------------------------------- 593

M1627_2112 GCTGCTATCTTAGAGCCTGGCGAGGCTAAGGATTTAGTTGACGAAATAGTTAAGAGAGTAAACGAAATTAAAGGTAAAACTTCAAGTTAA--------------------------------------------------------------------------------------- 584

SiRe_1905 GCTGCTATCTTAGAGCCTGGCGAGGCTAAGGATTTAGTTGACGAAATAGTTAAGAGAGTAAACGAAATTAAAGGTAAAACTTCAAGTTAA--------------------------------------------------------------------------------------- 584

LD85_2297 GCTGCTATCTTAGAGCCTGGCGAGGCTAAGGATTTAGTTGACGAAATAGTTAAGAGAGTAAACGAAATTAAAGGTAAAACTTCAAGTTAA--------------------------------------------------------------------------------------- 584

LS215_2197 GCTGCTATCTTAGAGCCTGGCGAGGCTAAGGATTTAGTTGACGAAATAGTTAAGAGAGTAAACGAAATTAAAGGTAAAACTTCAAGTTAA--------------------------------------------------------------------------------------- 584

YG5714_2158 GCTGCTATCTTAGAGCCTGGCGAGGCTAAGGATTTAGTTGACGAAATAGTTAAGAGAGTAAACGAAATTAAAGGTAAAACTTCAAGTTAA--------------------------------------------------------------------------------------- 584

YN1551_0761 GCTGCTATCTTAGAGCCTGGCGAGGCTAAGGATTTAGTTGACGAAATAGTTAAGAGAGTAAACGAAATTAAAGGTAAAACTTCAAGTTAA--------------------------------------------------------------------------------------- 584

M1425_2032 GCTGCTATCTTAGAGCCTGGCGAGGCTAAGGATTTAGTTGACGAAATAGTTAAGAGAGTAAACGAAATTAAAGGTAAAACTTCAAGTTAA--------------------------------------------------------------------------------------- 584

M164_2039 GCTGCTATCTTAGAGCCTGGCGAGGCTAAGGATTTAGTTGACGAAATAGTTAAGAGAGTAAACGAAATTAAAGGTAAAACTTCAAGTTAA--------------------------------------------------------------------------------------- 584

Ahos_0594 GCGGCTATTATAGATCCGGGAGAAGCAAAAGACGCATTAGACGAAGTCTTAAAGAAGTTACAAGAGATATCTGGTAAATCCTAA--------------------------------------------------------------------------------------------- 578

Saci_1520 GTAGCGTTAATGGATCCTGGTGAAGCTAAAGACCTGGTGGATGAGATAGTTAAAAGAGTTAATGAAATAAAAGGTAAAAGTAGTTGA------------------------------------------------------------------------------------------ 581

ST1424 GCGGCTATTATAGATCCTGGAGAAGCAAAGGATTTATTAGATGAGATTGTAAAAAGAGTAGAAGAATTAAAAGGTAAAGCAAGCTAA------------------------------------------------------------------------------------------ 581

ASAC_1012 GTGGCGATAGTAGATCCCGGTGAAGCGGGCGAGGATCTAAAGAAAGTAATAGACAGGGTAAAGGAGCTCAGGTCTAAGTCAGGTAAGTGA--------------------------------------------------------------------------------------- 584

Igag_0205 GCATGTATTGTAGAGCCAGGTGAGGCAAAGGCAGAGGTAGAGGAATTAGCATCAAAATTCCAAGAACTTAGAGCAAAAGCAGCGAAATAG--------------------------------------------------------------------------------------- 587

Tagg_1384 GCTGCGATCGTGGACGCTGGCGAAGTCAAGTCGATGGTTGAGGAGATAGTTAAGGCTGTCCAAGAGCTTAGGGCTAAGAGCGGTTAA------------------------------------------------------------------------------------------ 584

Shell_1642 GCTGCAATAATCGATCCAGGCGGTGCTAAGGACTTAGTAGAAGAAATAATAAAGCAGGTGCAAGAATTAAGAGCGAAAGCAGGAACTTAA--------------------------------------------------------------------------------------- 587

Smar_0825 GCTGCAATCATAGACCCAGGCGGTGCTAAAGACTTAGTAGAAGAAATTATCAAGCAGGTACAAGAACTAAGAGCAAAGGCAGGAGCCTAA--------------------------------------------------------------------------------------- 587

DKAM_1353 GTCGCAGTAATAGATGTAGGCGGAGCAAAGGACTTAATCGATGAAATAATTAAGAGTGTCCAGCAGATAAGGGCTCAGAGCGGCTGA------------------------------------------------------------------------------------------ 584

Desmu_1344 GTAGCAATAGTTGATGTCGGCGGAGCAAAGGACATAGTCGACGAGGTCATTAAGAGCGTTCAACAGATAAGGACGCAGAGCGGTTGA------------------------------------------------------------------------------------------ 584

Tpen_0493 GCCGCGATACTTGAACCAGGAGAGGCGGCTAGCCTCGTCGATGAAATAATCAAGGAAGTAAACAATCTGAAGATAAAGTCGGGATCTTAA--------------------------------------------------------------------------------------- 590

Igni_0230 GCCGCCATAATAGACCCCGGCGCGGCCAAGGACTTGGTAGAAGAGATAATCAAGGAGGTAGAGCAGATAAAGGCCAAGGCCGGGCTCTGA--------------------------------------------------------------------------------------- 587

APE_1818 GTCGCTATAATCGAGCCGGGAGACGCCGAGACCCTAGTAAGGGAGATTGTTGAGAAAGTGAAGGAGCTTCGAGCAAAGGCCGGTGTATAA--------------------------------------------------------------------------------------- 584

Hbut_0543 GCTTGCATCATAGACCCGGGCGAGGCTAAGAACCTAGTTGAGGAGATCGTAAAGGCTGTGAACGAGCTAAAGACAAAGGCAGCCCAGTAG--------------------------------------------------------------------------------------- 584

Pyrfu_0921 GCCTGCATAATCGACCCTGGCGAGGCCAAGGACCTCGTAGAGGAGATCATCAAGGCCGTAAACGAGCTTAAGGCCAAGGCTGGTGTTAGCTAA------------------------------------------------------------------------------------ 587

Cmaq_1846 GTCACCATAGTTGACCCAGGTCAAGCAACCAAGGACCTTGAGGACCTGGTGACTAAGCTAAATGACGTTAGGGTTAGCGCTGGCTTAAACCCAATACAATTACCGAGGACTCAACCACAGGCTAAGCCGCAGCAAAGGGAGAGGAGGG------AGAGGGAG--TGA---------- 695

Vdis_2417 GCCGTAATAATTGACCCAGGGCAGGCAGCCGCGGAGTTGGAGAATCTCGTTAAGCAGATAAACGATGTTAGGGTTAAGGCTGGTCTTAACCCAATACAATTACCGCAGCCGCAGGCCGCGCCGCCGGCTGAGAAGGCACCTGCCAAGAGGGCTGCCAGGAAGGGTGAATCCAAGTGA 722

VMUT_0759 GCAGTCATTATTGATCCTGGACAGGCAGGTGCCGAGTTAGAGAACCTTATCAAGCAGGTAAATGACGTTAGGGTTAAGGCTGGCTTAAACCCGATACAAATGCCACAGCCACAGGCTGCACCACCTGCTGAGAAGGCTCATGTTAGGAGGGCTGCCAGGAAAGGTGAGTCTAAGTGA 722

Pisl_0589 GCTGTAGTTATAGACCCAGGCCAAGCTGCCGGCGATTTAGAAGCACTAGTGGCTAAGATAAATGAGATAAGAGCTAAACACGGTCTAAACGCTATTCCTCTACCTAGTAGCGGCGCCGCTAAAAAGTAG------------------------------------------------ 710

Pcal_0443 GCGGTGGTAATTGAGGCAGGCCAAGCTGCAGGAGAGCTGGAGGCGCTGGTAAACAAGATCAACGAGATTAGGGCAAAACACGGGCTAAACGCCATCCCGGTCAGACGCTAA------------------------------------------------------------------ 650

Tneu_1580 GCTGTGGTTATCGAGCCTGGGCAGGCCGCTGGGGAGCTCGAGGCTTTGGTGTCTAAGGTAAACGAGATTAGGGCGAAGAACGGGCTTAACGCGATTCCGCTACCTGGTAGAAAGTAG------------------------------------------------------------ 656

PAE3347 GCCGTGGTTATAGAGCCAGGTCAAGCCGCAGGAGAACTAGAGGCCCTCGTCTCGAAGATTAATGAGGTTAGAGCTAAACACGGGCTAAACGCAATACCTGTGCCAGCAAAGAGGTAA------------------------------------------------------------ 656

P186_1272 GCGGTGGTGATAGAGCCCGGTCAAGCCGCGGGAGATTTAGAGGCCTTAGTATCGAAAATTAACGAGATTAGAGCCAAACACGGTCTAAACGCGATACCTGTACCGGCTGGCGGTAGGAGGTAA------------------------------------------------------ 662

Pars_1759 GCTGTGGTGATAGAGCCTGGCCAGGCTGCTGGTGAGCTAGAGGCATTAGTGGCAAAGATTAATGAGATAAGGGCAAAGAACGGGCTTAACGCGATACCTCTCCCGGCAGGAGCTAGGAGGTAG------------------------------------------------------ 662

Pogu_0372 GCTGTGGTGATAGAGCCTGGCCAGGCTGCTGGTGAGCTAGAGGCTTTAGTATCAAAGATTAATGAGATAAGGGCAAAGAACGGGCTTAACGCGATACCTCTCCCAGCGGGGGCTAGGAGGTAG------------------------------------------------------ 662

TTX_1999 GCGGTTGTGATAGATCCAGGCCAAGCCGCCGCCGAGCTTGAAGGATTTGTGACAAGGCTGAACGAAGTGAGGAGCAAATACGGCCTAGGGCCTATAGCTCCCGCAAGGAAATAA--------------------------------------------------------------- 653

TUZN_1501 GCGGTGGTGATAGACCCTGGACAGGCCGCAGGCGAGCTCGAGGGCCTCGTGGCGAGGATAAACGAGGCGAGGAGCAAGTACGGGCTAGGCCCTATAGCTCCGGCCAGGAAGTAG--------------------------------------------------------------- 590

Acidilobales, Desulfurococcales, Sulfolobales, Thermoproteales

Thaumarchaeota

TATA box TSS Kt-n SD Kt-b start

CSUB_C0428 ------aaacagccttag-ccacggccttgcctccgtgttcaacggcgttacg----caacgcctcctccaaaaccctgtcccggagcgtcttcatcccagtcaaggtcttaggccgtaaa-------------tttatgcatgcaacatgagctacg-------ctaaaaaccttgccacaat-agc-tttaagttctacac--c-------tc----ttgcaaagttcaaggagatgaaggcctATGCCCAATCCATACTACCAAACCTTCACACCGCCTGAGGAACTCGTGACAGCG 254

CENSYa_0293 -----------------------ggcccagcgggaccgagccgc----------tgatacgggtctatgcagaatcccattccgaggcggccctctctgagctgctctcggggcaggtccggaggatacgcgagatactagacggcgggtgaaggccggaattttgcgccaaaatccggataaaacacttttaagc-------------cctatggccgggccaccctcatggagagctgcaactgATGGCAAAGGGATACTATGTCAAGTTTGAGACGCCCAAGGACCTTGTAAACCCG 254

Nmar_0225 tccaagaaattgggttatgatccgacctagtggaacagaaccaa----------ttgttagagtatatgcagaagctcaaagtcaagaaaaattagacgatttaatgtctga------------------------------------atatctccaaaaagttaagacaatcatctccagataacacttttaaaaccaaaccaaaggatgtagggaatggagaatgaaccttagggtgacgaagtATGGGAAAAGCATATTATGTTAAATTTGAGACGCCAGAGGACCTCGTAAATCCA 254

CSUB_C0428 GCCCTTGAAGCGGTCAGACTGGCG---AAAACATCCGGTAAGGTGAAGAAGGGCGTCAACGAGGTCATCAAGGCAATAGAGAGAGGACAGGCCAAGTTTGTGCTCATAGCCACCGACGTCGACCCCCCTGAGATAGTCGCCTTCCTACCAACGCTCTGTGAAGAGAGGAAAATAAGCTACATCTTCGTCAACTCCAAGGCGCAGCTGGGAGAAGTCGCAGGCCTCTCTGTTCCAGCCAGCTCCGTCGCGGTCGTCGACCCCGGCGAAGCGAAGGGCTACATAGAGGAAATCGTGAAAAAA 551

CENSYa_0293 ATACTAGAGGCGCTCCG---GGCAGCCACGCAGAGCGGCAAGGTCAAAAAGGGCACAAACGAGGCGACAAAGGCCATAGAGCGCGGCACAAGCAAGCTTGTTGTAATAGCGGAAGATGTGGAGCCGCCAGAGGTGGTGGCGCACCTGCCGATACTCTGCGACGAGCAGGGGGCGGCATACGCGTTTGTGCCGAGCAAGCAGGATCTCGGCAAGGCGCTGGGCATTGACATTACATCTGCCGCAGCAATA---CTGGACTCGGGAGACGCGCAGCATATCGTAGACCAGGTGATAAGCTCC 548

Nmar_0225 ATCTTGGAGGCCGTAAG---AGTAGCTTCTACTAGCGGTAAAGTCAAGAAGGGAACTAACGAAGCAACCAAGGCAATCGAACGTGGTACTAGCAAATTAATTGTCATTGCTGAAGATGTAGAACCTCCTGAGGTAGTTGCACATCTTCCAATTCTGTGTGAGGAACAAGGAGCAGCATTTGCATTTGTTCCAAGCAAACAAGAATTAGGCAAATCATTAGGTATTGACATTACTTCTGCCGCTGCAGCAATTTTGGATGCTGGTGACGCACAACATATTGTAGACCAAGTTGTCTCATCA 551

CSUB_C0428 ATACAGGAGCTAAAGAAGTAG--------------- 572

CENSYa_0293 ATAGCCAAGATCAAGGGCGAGACCGGCAAATGA--- 581

Nmar_0225 ATTGCTAAAATTAAAGGTGGAAAGACTGATCAATGA 587

Cenarchaeales, Nitrosopumilales, Candidatus Thaumarchaeota

Other

TATA box TSS Kt-b start

Kcr_1017 atcgggagcagatggggtatctaggaagattagaagctttaaatttgagatgaagctggctacattcttgacaggatcctctaccccggatcagctgaggctgaggaga-----gcttacattctcctagagcccctgctctcaatgctcaggatcaggg--gaagatttttatacatctgagccacactggctgggggaatgaaggATGAGTAAGGACAAACCCTCCTACGTGAAGTTCGAGGTCCCCAAGGATCTCGTACCTAAGATACTTGAGCTCGTTCAGATGTCCAAGAGCACC 293

NEQ319 --accatggtctagggctatttataatgctttacc-attagataatgaaatagaaaaacaaacattggctttagcaatagagaaagactttgaaatactaactaaaaaacctatacttagagaatacaaaaagaagtatgaac-atttaaagaatttggtcgaccaactattataactttaaat--caa----tccctatttttcttcagATGGCTGAGAAACCACCATATGTAAAGTTTGAAGTTCCAGAAGAGTTAGCAAATAAGGTCTATGAACTGGTTAGAAAAGCC---AGAGAG 287 TSS verified by RNA-Seq data

Kcr_1017 GGCGGTAAGCTGAGGAAGGGAGTCAATGAGACGACCAAGGCCGTTGAGAGGGGAGAAGCTCAATTCGTCGTTATAGCGGAGGATGTCAACCCTCCTGAGATAGTCGCGCATCTCCCCCTCCTCTGTGAGGACAAGGGGATACCCTACGCCTACGTGCCATCCAAGGAGGAGTTGGGCAAAGCCTCCGGTCTCGAGGTATCGGCATCTTCCGTAGCTATAGTGGATGCGGGTCAAGCCAAGAGCTTATTAGAGGCCCTGAAGGAGAAGTTCAAGGAGATGAGGGGGGCTTAG 584

NEQ319 ACCGGAAAAATAAGGAAAGGAACCAACGAGACAACTAAAGCTGTAGAGAGGGGACAAGCTAAACTAGTTATAATAGCAGAGAATGTTAACCCTCCAGAAATAGTAATGCATTTGCCAGCATTGTGCGAAGAGAAAGGTGTACCATATGTTTATGTCCCATCCAAGGAAGAATTAGGTAAAGCAGCGGGAATAAATGTTAGTGCTGCTTCCGCAGCTATAATAGATGCAGGAGAAGCAAAAGACTTGTTAGAAGAAATTATTAGAAGTGTACAAGAACTAAAAGCTAAATGA 578

Nanoarchaeota, Candidatus Korarchaeota

Euryarchaeota

TATA box

Metin_1393 --------------------------------------------------------------gatatgtatgag-----------------atgtaatgc-aagaaatccatggaaagctgagaagtgtagaaagtgtggctataaagggttaag-accaaa--ggctaa-----gga--gccaagag---gataa---- 103

Mefer_1103 --------------------------------------------actccatgctttaggagcgagagata---------------------tattgatag-tgcctattgaaaggatattataaatata---tccaaaagttttatatactaaaa-gtcagtatgttgt-------ta--tacct---------att--- 109

Metvu_1339 ------------------------------------------------------ttgggagcgagagata---------------------tactcgttg-ttccgattgagagaatcttataaact------ccaaaacctttatatattaaaa-gtcgat--aagttg-----ata--tactcattc-----taa--- 100

MFS40622_0835 ---------------------------------------------------caataagttgttataccta---------------------ttcttgta----gccaatcaccaaataaaatagtat------tttgcatggtaaagtat-ataa-gcccttttgggctt-----ata--aatgcctttattaagta--- 106

MJ1203 -------------------------------------------------gtcaatatgttgttataccta---------------------ttctaagc----cacgatgataactacag---ggct------tttgcaggaaaatattt---------cttatataaaa-----ata--tgcaccttatagatgca--- 98

Metig_1662 --------------------------------------------------------------------------tataattt---------ttgtccgag-actataatcatagattattcaactgttc----agcaaacgactataaaccaata-accata--acacat-----ata--tactacttttgagtatagga 102

Maeo_0081 ------------------------------------------------------------------tatatgacttcaaataatacagaagtagccactc-ttttaattcattaattacaatt----gg-----------------gcccattcg-cccaaa--aattag-----tga--ataaccacgatgatatgagt 102

Metok_1174 ------------------------------------------------------------gtag-caatatgaaacttaattatat--taaaaattagat-----------taaatatttaaatacata----gttaaatctcaaaatatgctag-accata--atctat-----ata--tataactttgaaggatatag 112

Mvol_0393 -------------------------------------------------agtattgataaataccaattaataccattatttat-------ttttaatta-tttctaattattactatttttt-----------------------aactttttg-accgta--aatatt-----ata--tactattgtatagtatagta 110

Mevan_1553 ---------tcaaacagt-ttataacgcgagaaaaaatgagtgaag----atctttaagaattatatttactacccttaatgtttt--tatgggccataa-tttttaataataatgctatgttaatttt----caaaacccaaaactgttataaa-accaaa--actgtt-----ata--tatggctttacgatat-aga 168

MmarC5_0963 aatatcgacggaaacgttcttgttttaaatgaagaaagtggtgcaatcggtagtgcaatgattgcagaagatattttgaatggtaa---aaaagatattc-ttggaatacctgttgatttttaaatttt----t------------agactttac-accata--actatt-----ata--taccacttttcgatat-agg 169

GYY_03255 -------------------------------------------------------------------aagatatcctaaacggtaa---aaaggaaattc-ttggaataccggttgatttttaaatttt----ctaa--atttccagtagattac-accata--actatt-----ata--taccacttttcaatat-atg 112

MMP0641 -------------------------------------------------------------------aagatatcctaaatggtaa---aaaggaaattc-ttggaataccggttgatttttaaatttt----ctaa--atttccagtagattac-accata--actatt-----ata--taccacttttcaatat-atg 112

MmarC6_0248 ---------ggaaatgttcttgttttaaatggggaaagtggtgcaatcggcagtgcaatgattgcagaggatattttaaatggtaa---aaaggaaattc-ttggaatatcggttgatttttaaatttt----ctaa--attttta-gactttgt-aacata--actatt-----ata--taccacttttcgatat-atg 169

MmarC7_1665 ---------ggaaatgttcttgttttagatggggacagtggtgcagtcggcagtgcaatgattgcagaggatatattaaatggtaa---aagggaaattc-taggaatatcagttgatttttaaatttt----ctaa--attttta-gacttcat-accata--actatt-----ata--taccacttttcgatat-atg 169

Mlab_1497 ------------------------------------------------------------atc--------agcgatgaacgttatc-caatga-cgttc-atccatatctttatctcaaat----tca----g--acccataaatatagttacg-ccagca--acggtt-----acc--ccgtacgggggg-------a 102

Mpet_2104 -------------------------------a----------------a-aatttatc----------tgcccttttttttcttttc-ccacccgtccgg-tgccggacggaaaatcaaaat----tcg----a--aaacctttatccatccaca-tgacta--aataa------------tatagct-----------a 104

Mhun_1601 -------------------------------g----------------a-actctaccggaatcgcgatgtcgtgatctaccagcat-caggcttgatga-aacccggccagtcgg----------gga----g--atatttcccttggaaacaa-aaccag--atcta------------cgaacctttaa-------a 112

Memar_0721 ------------------------------------------------------taccggatc-------cccgcataaaccgtctg-cggcca-cgtcc-atgcgggcaggcacgaaaacg----acg----g--aaccctacctccaaaaacg-tgggag--aatga------------caaacctttat-------c 104

Mboo_0690 ----------------------------caag----------------t-gtactccggggaag---gtaccgagatttaccggctt-agtggg---tga-tgcgtggcagcccggtttgat----tcc----c--tg-ccgggcagtggtctcc-cctcac--gctca------------caaacctttat-------t 114

Mpal_0523 --------------------------tctgtg----------------t-cagatcccgggtca------gagggatgatccggacg-ggcgag---gat-ctcctggattctggggaaaat----tca----g--gtagatcaccaggtccctg-gcctcc--agtta------------caaacctttat-------g 114

MCP_1041 ---------------------------------------------------gacg--aaaa---------ggtgccgccggcaaagc-t--------tta-gcactatccgaagcaaaaact----tta----a--aaacta--tacggctatta-tacaga--cacgct-----cgc--agg--cttatatggccctgc 104

LRC145 -----------------------------agg----------------c-cggcgaaagaa---------agcggctcaaccgatgc-c---------gt-agccggctcaaagcaaaagat----tta----a--aatgta--tcccgatatta-taggga--cacgct-----tga--tcg--acattatgttgtcga 110

MCON_1706 --------------------------tccagc----------------a-gtg-gatcagaa--------ggagagttatc----------------cgg-atttgtttccgaggaaaacat----tcg----g--tttgtgctgatggggcttc-t---tg--ggccct-----agc--gcaacctttttacatgtgga 108

Mthe_0331 ----------------------------gagc----------------a-tctcgcgcgacg--------gcagcattttgcgactc-ga-------tgg-aaccatcgcaccggaggagat----cag----g--ggcgtgatcgcggatctgc-ttgcgc--cctgat-----agc--gtaaactttttagataggga 118

Mbar_A3388 --------------------------------------gt-----------ttataggtgatcatgta--cttctaaggttcgatct-g--------cag-catttagcta----gcagtat----cta----t--attt------caactc-------------attta-----ctc--tccgcattgaagtctatact 99

MA1521 -----------------------------------------------------ataggtgattatgta--cttgtaaggtgcagtct-g--------cag-catttagcta----gcagtat----cta----t--gttt------caactc-------------attta-----ctc--tccgcattgaaggtttttca 95

MM_2467 ---------------------------------------------------ttataggtgagtatgta--cttttaaggttcattct-g--------cag-catttagcta----gcagtat----cta----t--gttt------caactc-------------attta-----ctc--tccgcattgaagcacttcag 97

Mzhil_1258 -----------------------------ctg--------------------------------------------------cactg-t--------atg-gatttagcat----aaaatat----tta----t--gtaaatgaaatgattcata-tcacaa--aaccta-----atg--tcagaatatactacaaaatt 87

Metev_1406 --------------------------------cacaacca-----------cattccagtatgaataa--caccaatcaaacccagc-c--------atg-attatagcgt----aaaatat----tta----a--ctaaaagtcacaattcat--------------------------------------agccagca 93

Mbur_0219 ---------------------------------atttttt-----------ttattttgccttgtgca--tgttttggggaactggc-a--------gac-actttggcgc----aagctat----ata----t--gtgactgtcgcgattcat--------------------------------------accaaata 92

Mmah_0863 ----------------------------gtaccagaagtt-----------ttata----atcgatag--gcatattcaagaagtgc-t--------atc-gaattgtcgc----aagttat----ata----t--gtaccagtggcgattcat--------------------------------------accaaata 93

Mfer_0452 ----------------------------atactattgtttttatctgca-attatgtgtggt----------------------------------------cttggccttcaaccataaaat---ttt----t--actgcgaaaaatatttata-taac--------ta-----ttg--taaaaatttttgtacatcta 106

MTBMA_c07050 -------------------------------------------------------cctgggtgtctaactggattatccgggattaa-t--------cta-agtacgatttctgatgaaaatc-----t----g--tatccagcaagttttatat-acagag--a---ac-----ata--taaagggtactacccac--- 108

MTH255 -----------------------atttctattaatcctt---------g--ttttcctgggcgtcttactggatgatccgggataat-t--------cag-aatacgatttctcatgaaaatc-----t----g--tatccagcaagttttatat-accgag--a---ac-----ata--taaaggtactcacccac--- 129

mru_1490 -------------------ctgtaattgctttaattattaaaataataa-attttatttgttctatct----tgaatatatgaattt-a--------tat-ttttaagctaaggaatcttattt--cta----c--gcttaaggtaattt---ta-ttttaa--aattaa-----caa--ttactgttttaattattata 145

Msm_0206 ---------------------------------------------tggg-attatatttagtgatatatgaatgtattcaagaatta-g--------tat-tattaaaaactataaaattat------t----c--gctcatt----------------gaa--taattt-----gag--taatttctatagttaaaata 107

MSWAN_1751 ----------------------------------------------------------------atgatgatgaaacatttaataag-c--------taa-ttttgaacttctgaaaattattt--att----a--gtttaaatgagttttgaaa-ttagaa--tttgat-----tta--ttaaaaattcattcaagtca 108

Msp_0633 ------------------------------------------------------------atcactcattaaggagttttaaattag-a--------ata-ttttttatta----tataaatat--att----t--tattaaataggtcatttaa-tcagac--aacaag-----tcg--ttatagatataataaaaata 108

Faci_050500008291 -----------------------------------------------------------------acc--ctgtgttgaagaattgt-g-cagc---t--------g-gagttttaacatgaataaatg--gcaattccatagacccgcgttagtgtagttcataaaagcaa----------t-tatt-tgttttaatat 105

PTO1280 ----------------------------------------------------taatacc-atta-----------atgaaaaagctt-g-ga--------------------aaataccagtttccaaa--tgcatactatgagtacttctacaa--aaagaataatataaa--tggt--gga-ta-c-aggcctccaat 103

Ta1116 --------------------------------------------------gttttcccttatggaagc--taaatttataaaagttt-c-cagc---g--------t-cagcgaaaaattccattctaa--atagttatatatcggcaaatcatc--atctcgttgcatc------------a-tacg-aggtcttg--- 113

TVN0449 -------------------------------a----------------a-agtttccccaatggggca--taacgttgca----------------------------------caagctaaattctaa--agcgttatatatgagctcacaatc--ccatcatactctaacatttaa--cct-tagc-ctgccctcgat 108

Arcpr_0494 --------------------------------------------------------------------------cattagctcatat-a-aaac---tgc-tttgtgtag----taaataccgtaagtt--tg-gttgttaatcgattgtaggtt-gctacttttccaac-aataaaagaaag-caagcacgagatcgaa 110

Ferp_0621 -----------------------------------------------------------------------------aaattctgct-c-aaag---agc-tcgaaaaaggtgacgcaaattatagaat--tg-cgatttattctctcatcgatg-tctccagaaacctc-aaatcgatagga-gaagttg--cttttaa 109

AF0764 ------------------------------------------------------------------------gtggtgagaaatatt-g-aaag---tcc-tagcaacaggtctaagctcagtttagct--tg-gttgtccatatcataaaaaat-aggat-----gtt--aatcc---------atttaaatcttgtga 102

Arcve_0453 -------------------------------------------------------------------------ttccctcacaggct-g-gcag---ttc-gagaaagacttttctgca--------ct--tt-tttctccggggcacaaacaga-agtaa-----gatt-tctcgaagataa-ccttttcctccgctac 102

Aboo_1236 -----------------------ac--aaaaa----------------a-att--aaa----------------------aataact-a-ccat---a---ctaacgggggtgcataccccatacctac--ag-aagcgagaagcga--gataca-taacggaagagatagg---aggcataaatgacctgatttcagtg 117

HQ2885A ------------ggcgca-c-gact--cagca----------------c-ttcttagaccaccgcgat--ggagcatgtgtcggttt-c-gcac---gcg-acaccggcattgaaagcgtttttatgca--atccaagattaaatctg-tttacg-acctgcacggggt------tga--tgatactcctcgccgtcag- 146

VNG1157G ---------gccggcggt-tgggcg--acca-----------------c-gccaaagggcgtttcgtc--cgcggctgtgcagccgt-g-gcac---ggc-cgcccggcattgaaagcgcttttacggg--cgggcgacccactgttgggataca-gcctgcggggg-t------cga--tgatgctt------------ 138

OE2662F ---------gccggcggt-tgggcg--acca-----------------c-gccaaagggcgtttcgtc--cgcggctgtgcagccgt-g-gcac---ggc-cgcccggcattgaaagcgcttttacggg--cgggcgacccactgttgggataca-gcctgcggggg-t------cga--tgatgctt------------ 138

Hlac_1842 ----------gaaccgct-gcgaac--gccgt----------------g-gc---taaagcgggtcct--tccccgtgagccccgct-c-gcac---acg-cgcaggtcattgaaagcgcttttatgga--tgtccggactaggtcgc-ttcaca-gcctctgtggggt------tga--tgatgtgccgttcc------ 141

Halar_2173 -----------------t-tggccc--gtccg----------------a-ggtttcccggtttcggcc--ggcgcgtgccggcatct-c-ccat---acg-gccgcgagtttgaacacgcttttacgta--ccccagccaaatcgacgggcacga-gcctgttcggggt------tga--tgaccctcctcgc------- 137

HVO_2737 ----------------------------------------------------------------tcgc--gggtcgtgcggacgcgt-c-gcac---ggc-gctacgattttgaaagcgcttttatggg--ggccggagcaaaggatg-tttaca-gcctgtccggggt------tga--tgaacctcctcgccgtaggg 116

Hbor_12060 ----------------------tcc--acgac----------------g-acgtgaacgtcccgagaa--cgtgcgtgcgggcacgt-g-gcat---ggg-aggaaagcattgaaagcgtttttatggg--gtcctgagctatcagac-tgtaca-gcctgcccggggt------tga--tgaatgctcctcgccgagag 139

HacjB3_08675 -----------------------------------------------------------------------gaccgtcgtcctttat-t-ccac---a---tacaaacacgttatgactattttcgtgt--gtccggctcacacggcc-tctacc-gtttgaacacgctt-----tta--tggggtggtgcgctactgaa 110

Nmag_0451 ----------------------------------------------------------------------------------gacgt-a-gtac---accctacgcggggttgatgatgctcctagcct--t-acaggaccgccgccg-ggcaca-gcccggcctggggttcggtttc--cggagccggagtcgatgccg 106

Halxa_0717 -----------------------------------------------------------------------tgccgtgcggactcat-c-ccac---ggc-cccagccgtttgaacacgcttttaaggg--gggcaacgctactgacg-tagtac-accctacgcggggt-----tga--tgatgctcctagccccacag 112

Htur_2527 -------------------------------------------------------------------------ctgtgcggacacgt-c-gcac---ggc-tccgtgcgtttgaacacgcttttaaggg--gggcaacgctacagacg-tagtac-accctacgcggggt-----tga--tgatgctcctagcctcacag 110

NP3660A -----------------------------------------------------------------------------gcggtggagt-c-gcac---gcc-gagcggcgttcataagcgcttttatgca--ggggtgcagtacggtgg-tgtaca-gcctacgcgggg-------ttg--tgatgctcctagccggaagg 104

Huta_0750 -------------------------------------------------------------------------------------------cac---gcg-caagcggcttcgaaagcgcttttacgtc--cg-ccggaccacgttcg-ggtaca-gcctgcacggggt------tga--tgatactcctagccatctag 92

Hmuk_2621 -----------tctcgga-cgggccagacgac----------------c-gaagcgatccccggccag--cgaccgtgtggcggcct-g-ccac---ggg-atttcggcttcgaaaccccttttagggc--tc-gtgtcgcagtcacg-ggtaca-gcctgcaccgggt------tga--tgatgctcccagccttctca 150

HAH_0858 -----------------------------------------------------------------cag--cacccgtgtgccggcct-g-gcac---gcg-atttcggcttcgaaagcgcttttagggc--cg-gtggcacagaatgg-ggtaca-gcctgcgccgagt------tga--tgatgctcccagccttttca 114

rrnAC0103 -----------------------------------------------------------------cag--cacccgtgtggcgggct-g-gcac---ggg-atttcggcttcgaaagcgcttttagggc--cg-gtagcacagaatgg-ggtaca-gcctgcgccgagt------tga--tgatgctcccagccttttca 114

MK1598 ----------------------------------------------------tctaaccacgatcacc--tgtttgtacaaggtgaa-c-cccc---t----cggct---------gagaggagtctcg--tc-acagactc--ggc--ccgtca-gctcctcttcattggggcccgt--------a--agacaccggcg 110

TERMP_01118 ----------------cc-cgccag--cgatc----------------c-cggttgtact-------g--ccctccgagagggggcg-g-gaag---c----cggggacgccgccggccactcaaactg--tt-tttctgttgagat--tttaag-aaatcagtacactttagttggtgaaga-ttt--ttagtttcgag 137

TSIB_0419 -----------------t-atatag--agaaa----------------c-tcaatggc---------g--aatacggagataaagtg-g--------------aggtaaggggaataagggttgagccg--g----tgtttatagca--gataag-gaatatattctttatgaggggtgatgg-ggt--tctcttttct- 124

GQS_00200 -------------------------------c----------------c-ttcatgaggtctacctca--aaccttt--------------ccc---g----gagttccgggggaagggccttaaggaa--ct-cgt--aca---tc--ttcatg-gcct-------cagacatggta--ttc-gtttcggtttagggct 108

TON_0954 ---------------------------gtggc----------------t-atgatgagaacgagtg-------agtt--------------ctc---t----caggtcattttcgtttccctggctgac--ct-ctgggatg---ac--gagctt-ggcc-------gctgctgatgg--cgg-ctgtcccgttctgctt 109

TGAM_1876 --------------------------------------------------ctgataaggccgat--cg--gtctaacatacggctac-----------------cttccagagactggga-tgtgcttc--cg-cttctttc---tt--ccga-a-gggg-------gtttacgcgac--gag-cgtcctgcgcgagata 108

TK1311 ---------------------------------------------------tcatgtccactacgcgg--gttcaatagcccagttc-c-taac---g----gagttcacgggaaagaga-tgggaggg--ca-aggaaacc---aa--gtgcga-ggcc-------atgggcgcaga--tca-ctgcgagtttgtgttc 118

PFC_06055 --------------------------------------------------ggttttaaggctgaaacc--acccagtaaataatggt-t-tca-----------------------gacttcatgttcc--cc-cttctttt---tc--taaagt-actgccagtaacttaaacctgacaccccacagcttatcctaaca 114

PF1367 ------------------------------aa----------------g-ggttttaaggctgaaacc--acccagtaaataatggt-t-tca-----------------------gacttcatgttcc--cc-cttctttt---tc--taaagt-actgccagtaacttaaacctgacaccccacagcttatcctaaca 117

PYCH_03310 -----------------------------------------------------------------atc--agctcgtagataaaaac-t-aggg---c----t-attatagtgagcaaccctgccgttg--ca-ggaaagta---tt--taacta-gattcctcaagttagcccccatcgcctgagtgccttcaacttta 114

PNA2_0082 ----------------gc-tgatac--agagc----------------a-gtgtcgaaaccgactatc--agctcatatatgaatgt-c-aacg---t----c-atgagcactataagcgcgaaaaccc--cg-gggaaata---cc--tatgaa-tcttca---------------------------tcatcttgaac 116

PAB0460 -------------------------------c----------------c-ctgtcgaagcccacgatc--acttcatagataaaaac-c-gccg---t----g-atgataatcatcaaggagaacgtcc--cg-ggaaagta---ct--tatgga-gcctcaa--acttctccccacttcaagtttcccaggaactttaa 129

PH1496 ------------------------------------------------------------caaccaca--agttcgtaaataaagac-g-aggg---t----t-gttagaattataagtgaaaatgtcc--ct-gggaagta---ct--tatgta-gcctcaa--atctttcacctttcctaaattttcaggcgatt-ta 116

TSS Kt-n Kt-b SD start

Metin_1393 ---cttatttatatcactttactctttaattatctaaaactttttaggggcaatac---------ctccgg-attgccctattttttaaatagatgaaggagggatagaaATGGCAATATATGTTAAGTTTAAAGTTCCAGAGG---------ATTTACAAAAAGAGCTTTTAGATGCTGTAGCTAAAGCAGAGAAGATA 281

Mefer_1103 ---ataagccacgatgatactatagggcacagaagccctgtaggagtgggcaatt---------------c-ctccggattgcccataaaagatgaaggaggttgaaaatATGGCAGTTTATGTAAAATTTAAAGTTCCAGAAG---------ACATTCAAAAAGAGTTATTAGACGCAGTAGCAAAAGCACAAAAAATT 281

Metvu_1339 ---accacgatgacaactatgggttaagataataaacccataggagtgggcaattccctcc----ggattg-ccctcaatttttaa--aaagatgaaggaggtagaaaatATGGCAGTTTATGTAAAATTTAAAGTTCCAGAAG---------AAATTCAAAAAGAGTTATTAGATGCAATTGCAAAAGCACAAAAAATT 281

MFS40622_0835 ---at---aaagtttggtgattggctacaatccacgatgatacgagtgggcaattccctcc----ggattg-cccattttt-----aaaagatgaaggaggttgaaagatATGGCAGTTTATGTAAAATTTAAAGTTCCAGAAG---------AAATTCAAAAAGAGTTATTAGATGCAGTTGCAAAAGCACAAAAAATC 281

MJ1203 ---aaattccttataaatatcaacaagtgcaaaagccctgtaggagtgggcaattccctcc----ggattg-cccattttttagcaaagagatgaaggaggttgaaagacATGGCAGTTTATGTAAAATTTAAAGTTCCAGAAG---------AAATTCAAAAAGAGCTATTAGATGCAGTTGCAAAAGCACAAAAAATC 281

Metig_1662 t--aattgccactc---------ttttacaaccatgatgatatgagtgggcatatatccggatgacaacca-cattttttaaattataaaaaatgaaggaggttatatgcATGGCTATCTATGTAAAATTTAAAGTTCCTGAAG---------AAATCCAAGAACTCACATACAAGGTAGTTTCAAACGCAGAAAAAATT 281

Maeo_0081 gggcatggctacactattaaaaccaataataaaatggtaataa-tggtgtaggtatccgga-----------taaaatatcttttttaaaaaatgaaggaggtcattattATGGCAGTATATGTAAAATTTGATGTTCCACAAG---------AAATGGAAGAAAAAACAGCAGAAGTGCTCTCAAAAAGCGAAAAAGTT 281

Metok_1174 gcactagccactct---------ttttaataccatgatgatatgagtgggcataatccgga-----------taaaa--aacgatttttaaaaatgaaggaggtcatactATGGCTATATATGTAAAATTTGAAGTTCCACAAG---------AAATAGAAGAAAAAACAGTAAATGTAATTTCAAAATCAGAAAAAGTT 281

Mvol_0393 gtgtaccactcttt---------taataataccatgatgatacgagtgggtgccatccgga-----------taaaaaaaatttttttaaatagtgaaggaggtcataatATGGCTGTTTATGTAAAATTTGAAGTACCACAAG---------AATTAGAAGAGAAAACTGCTGAAGTAGTTTCCAAAGCTACTATGATT 281

Mevan_1553 ctatctgccaactc---------ttttaataccatgatgatATGAGTGGGCGCCATCCGGA-----------TAAA-AAAATTCTTTTAAGTAATGAAGGAGGTCATAATATGGCTATATATGTTAAATTTGATATACCACAAG---------AGCTTGAAGAGAAAACTGCTGAAGTAGTGGCTAACGCTGAAAAAATT 338

MmarC5_0963 gt-agttgccgctc---------ttttaacaccatgatgatATGAGTGGGCGCCATCCGGA-----------TAAA-AAAATTTTTTTAAGTGATGAAGGAGGTCATAATATGGCTGTATATGTTAAATTTGAAATATCACAAG---------AACTCGAAGAAAAAACTGCAGAAGTAGTTGCTAACGCTGAAAAAATC 338 TSS verified by RNA-Seq data

GYY_03255 gt-agttgccgctc---------ttttaaaaccatgatgatatgagtgggcgccatccgga-----------taaa-aaaatttttttaagtgatgaaggaggtcataatATGGCTGTATATGTTAAATTTGAAATATCACAAG---------AACTCGAAGAAAAAACTGCGGAAGTAGTTGCTAACGCTGAAAAAATC 281

MMP0641 gt-agttgccgctc---------ttttaaaaccatgatgatatgagtgggcgccatccgga-----------taaa-aaaatttttttaagtgatgaaggaggtcataatATGGCTGTATATGTTAAATTTGAAATATCACAAG---------AACTCGAAGAAAAAACTGCGGAAGTAGTTGCTAACGCTGAAAAAATC 281

MmarC6_0248 gt-agttgccgctc---------ttttaaaaccatgatgatATGAGTGGGCGCCATCCGGA-----------TAAA-AAAATTTTTTTAAGTGATGAAGGAGGTCATAATATGGCTGTATATGTTAAATTTGAAATATCACAAG---------AACTCGAAGAAAAAACTGCGGAAGTAGTTGCTAACGCTGAAAAAATC 338

MmarC7_1665 gt-agttgccgctc---------ttttaaaaccatgatgatATGAGTGGGCGCCATCCGGA-----------TAAA-AAAATTTTTTTAAGTGATGAAGGAGGTCATAATATGGCTGTATATGTTAAATTTGAAATATCACAAG---------AACTCGAAGAAAAAACTGCAGAAGTAGTTGCTAACGCTGAAAAAATC 338

Mlab_1497 ---actactc---aggatttattctgagac---tgttttgtacctaccagtatgaccgctgatgagcgatgacagagggtaaaacctctcgagtgaagtgagtaattATGGCAAAGATTTACCAGAAGTTTGAAGTCCCCGAAGAACTTCAGAACAAAGCACTTGAAGCGCTTGAGCTTGCACGTGACACCGGAAAGATT 293

Mpet_2104 ---catcgta---aactgatgagtgatgaa---ggagtaactcaacacatattccggaaaggtcattccgcctgt-tccg-gctcttgctcctgaatgaggtgagttATGTCAAAAGTATACGTAAAATTTGAAGTTCCAGATGAGATCCAGAACAAAGCTCTCGAAGTTCTCGAGATTTCAAGGGACACCGGAAAGATC 293

Mhun_1601 ---cgcccac---aatcgtaact-------------aatatggcacattcgtcatctgctgatgagtgatgacggagggtcatgcccctcctgaatgaggt---gaattcATGGCAAACTACGTTACCTTTGAAGTCTCTGAAGAGATCCAGAACAAGGCTCTTGAAGCCGTCGAAGCCGCACGTGAAAGCGGAAAAATC 290

Memar_0721 ---actcctc---ttattgacataatagag---cgcattcgatcagctacgctactgatgaatgatgacggagcagcag--tgggctgcccctgaaagaggtgagttATGGCAAAGGCATACGTAAAATTCGAGATTCCGGAAGAGATCCAGAATAAGGCTCTTGAAGCCCTTGAGATAGCAAGGGACACCGGTAAGGTG 293

Mboo_0690 ---tctcctc---ctgatgaaat------------gatat-ggctgcatcactgctgactgatgagtaatgaaggggca--aatcggctcctgaatgaggtgagtttATGGCAAAAGGCTACGTAAAAACCGAGGCTCCTGAGGAGCTCCAGAACAAGGCGCTTGAAGCCCTTGAAGTTGCAAGAGACACCGGGAAGATC 293

Mpal_0523 ---tgtggct---aaaagagaaa------------gatatggacacatcggcgactgactgatgagtgatgaaggagac--tacgcaatcctgaatgaggtga-gttATGTCAAAAGCATACGTAACATTTGATTCTCCAGAGGAGATCCAGAACAAGGCCCTTGAAGCCCTTGAAATTGCCCGGGATACCGGGAAGGTC 293

MCP_1041 ---agccgttgcaggcatgaagttgcccgc---atacgtaattattaggttattatacctgatgattgatg--aagggcgaaagccctgaaggaggaactcataATGGCAAAACCGGCTTATATCAAGTTTGAAGTACCGCAGGAATTACAGGACAAGGCCCTCGAGGCCGTAGAGCTCGCGAGGGACACTGGCAAGATC 296

LRC145 ---caggcctgttcgtttaatctt---------tactttaggttaaacctgatgaatgatgaagaggcttc--ctgatagaagccttcgaaggaggaaattaacATGGCAAAACCAACTTACGTTAAGATGGACTTCCCGCAGGAATTACAGGACAAGGCTCTCGAGGCACTCGAGCTCGCCAGGGACACCGGCAAGATC 296

MCON_1706 ---actaatggcagatctagtcttgaggt-------ataatccaaaaggc--gcttttgcgatgaatgatgatgggcgttgtcgcccggaaggagaattaagttATGGCAAGACCAATATATGTAAGGTTTGATGTTCCAGCAGATTTGGCCACAAAATCCCTTGAGGCCTTAGAGCTGGCTCGCGATACCGGCCGAATA 296

Mthe_0331 ---gatggagctggatctgttc---------------attcgagtgaggc--aattttccgatgaatgatgatg--ggctggtgccctgaaggagatgtgtgatATGGCTAGACCGATATACGTGAGGTTTGATGTTCCACCCGAGCTGGCATCAAAGTCGCTTGAAGCGCTTGAGCTGGCTCGCGACACCGGAAGAATA 296

Mbar_A3388 ---atttcgatttaa-----tatttgatttc-aatgttgacagatgtgtgttaaaaaaccaatgattgatgaaaaattgcgagactcgcaatttcgaaggagaatcttatATGGCACAATTAGCTAAATTCGACGTTCCAGAAGAACTTACAAACAAAGCACTTGAAGCTCTTGAGCTTGCCAGAGACACTGGAAAGATT 290

MA1521 ---ggttctttttttaggaatttgaattttc-aatgttgacagatatgtg-ttaaaaacctatgattgatgaaaaattgcgagacccgcaatttcgaaggagaaacttaaATGGCACAATTAGCTAAATTCGATGTTCCTGAAGAACTCACAAACAAAGCACTTGAAGCTCTGGAACTTGCCAGAGATACCGGAAAGATC 290 TSS verified by RNA-Seq data

MM_2467 ---gtt--cttttcttagaatttgaattttc-gatgttgacagatatgtg-ttaaaaacctatgattgatgaaaaattgcgagatccgcaatttcgaaggagaaacttaaATGGCACAATTAGCTAAATTCGATGTTCCTGAAGAACTTACAAACAAAGCACTTGAAGCTCTGGAACTTGCCAGAGACACAGGAAAGATT 290

Mzhil_1258 ---atgtagctgacagaaagattacagtttatcatcaaactgaatatttattgaaaatgcaatgaatgatgaaaaatcacgataccagtattgtgattttgaaggagacaaaaaaaATGATGAAAAAATTAGATGTTCCAGAAGAACTGGTCAATAAAGCATTAGAAGCGCTTGAGATGGCAAGAGATACAGGCAAAGTC 284

Metev_1406 ---cttaagctgtaaatattacagtcaatatacagtcaa----tacagtcaaaagcaaacgatgaataatgaaaaattatgat----ttaatatcataattttga-aggagataaaaacATGAAAAAGTTTGACGTACCAGAAGAATTACAAAATAAAGCACTTGAAGCGGTAGAGTTAGCAAGAGACACCGGAAAAATC 281

Mbur_0219 ---ctttggttgatgtaatatcactcatttctgagtgaatgtattgctgatgtgaattacaatgaataatgacaaaccgcaga----c---aaaggcggtttcgaagga-gatatgaatATGGTTAAATTTGAAGTTCCTGACGAATTAGCAGATAAAGCACTTGAAGCAATCGAACTCGCAAGAGACACCGGAAAGATA 281

Mmah_0863 ---ctccaacgaatgtaaacagtatggtagaattgacctt-----tgtttgcatctatacaatgaataatgacaaaccgcggt----gttaaaccgtgatttcgaaggagatagaaattATGGCAAAATTTGATGTTCCAGATGAACTAATAGATAAAGCTCTTGAAGCAGTAGAAGTTGCCAGAGATACTGGCAAGGTA 281

Mfer_0452 ---tttctccaaaattttaaagggaaga-----gaaagtatatattgttggttgtcgtagcttttattaca--aactgagccaatgagttggaggtgatgaaatATGGCAAAACCATTCTATGTGAAATTTGATGTACCAAAAGAGTTAGCTGACAAAACATTGGAAGCCGTGGAAATAGCAAGAGACACGGGAAAAATT 296

MTBMA_c07050 -----ctcatgttaatccataacttggatt---agttgtc--cagattggtgtaattaccgatggatggta--gaaaaaccagatgaaaaaggaggtaaataacATGGCAAAGGCAATCTATGTGAAATTTGATGTACCAAAGGAACTGGCTGACAAGGCCGCAGAGGCACTTGAAATAGCCAGGGAAACAGGTAAGATA 296

MTH255 -----ttcatgttaatccataacttggatt---attgatc--cagattggtgtaattaccgatggatggta--gaaaaaccagATGAAAAAGGAGGTAAACAGCATGGCAAAAGCAATATACGTGAAATTTGATGTACCAAAGGAACTGGCTGACAAGGCCGCAGAGGCACTTGAAATAGCCAGGGAGACAGGTAAGGTA 317

mru_1490 ---attgtattttattaacaaattaaattc---ataacttatacagttataatatttgccgATGG--ATGG--CAAAAGCCAGATGAAAAAGGAGGTAAATTATA---TGGCAAACATTTATGTAAAATTTGACACACCTGAAGAAATCGCTAACAAAGCAGAAGAAGCTTTAGAAGTAGCACAAAATACTGGTAAAGTA 332

Msm_0206 ---atataactatatataattacgaatatt---ataattcaaggaattataatatctgccgatgg--atgg--cata-gccagatgaaaaaggaggtatatattATGGCAAAAGCAATTTATGTAAAATTTGATACACCTGAAGAATTAGCTAATCAAGCTGAAGAAGCATTAAAAACCGCACAAGACAGTGGTAAAGTA 296

MSWAN_1751 ---t-ttgctgat-cgacaacttttatata---tttagaatataaaaagtaagatctgccgatgg--atgg--ttaaaacccagatgaaaaaggaggtaattaaATGGCAAAAGCAATTTATGTAAAATTTGATGTACCAAAAGAAATAGCTGACAAAGCCTACGAGGCTTTAGAAATAGCAAGAGACACAGGTAAAATA 296

Msp_0633 ---g-ttttttaa-aagtatattttaaaaa---attggtagttatctaaatagtacttgctatgg--atgg--ttaaaagccagatgataatggaggacattaaATGGCAAATTCAGTATATGTAAAATTTGAAGTACCAAAAGAAATAGCTGATAAAGTTTACGAAGCTTTAGAAATCGCAAGAGATACAGGTAAAATA 296

Faci_050500008291 gc-atattttttgaa---------att-ccttaatgttcatgttttctaa-tgctaaatatttatattgcagtttataataccttgcagaagtgaaggaggattatcATGGAAAACAGTTATGTAAAGTTTGAAGCTCCCGAATCGTTAGTCAAAGATGCATTGGATTTTGTAGAAAATTCCTACAGATCTGGCAAGATT 293

PTO1280 gt-acagggtaggca---------ttgaaatataaattaat--attttaa-ttactaatctttatatcattgaatacaataccttttaagaacatgaaggaggaataaatATGGAATCTTATGTAAAATTTCAGACTCCCGAAACGCTAGAGAAGGCAGTTCTGGATATGGTTGAAAATTCATACAAGACGGGCAAGGTA 290

Ta1116 -a-ta-ccagatgca---------tgacgctt-gaaataaa--atatata-tgcgtttggcatatgggtaaagcccgt----atattgaaatgaaggaggtaatgatATGGAAAAGAGCTATGTGAAGTTTGAAACTCCCGAAGACGTTTCGCAGAAAGCACTGGATCTTGTGGAGAGTTCATACAGAACTGGAAAGGTC 293

TVN0449 ac-at-aggtatcta---------acgggcct-caaataaa--ataaata-tatgattggcattaaggttaagtttagaagatttaaaagtgaaggaggtaattgaaATGGAGAAAAGCTATGTAAAGTTTGAAACTCCCGAAGATGTTTCGCAGAAGGCTCTTGACCTTGTGGAAAGTGCGTTCAGGAGCGGAAAGATC 293

Arcpr_0494 aa-ct-attgtg------------aacccaaaggtttaaaaagcgattgc-aaaattcgag----acttaccgatga--atgatgacaaatagg--ggaaggaggtgatgggaATGGCGTATGTAAGATTTGAAGTTCCTGAAGAGATACAGCAGGAAGCTTTGGCTCTGCTTGAGAAGGCGAGAGAGACAGGAAAGATA 287

Ferp_0621 ca-aa-gccgtg------------a-gggaaaggtattt-aagctgaatc-atctttcgtctcttctcagccgatga--acgatgacagaagtgtgaaggagggatgagtATGAGCGTGTACGTGAGATTTGAGGTTCCTGAGGAATTGCAGCAGGAAGCCCTTGCTTTGCTTGAAAAGGCGAGAGAGACGGGAAAAATA 290

AF0764 tt-accatcatc------------acatatcagtgttaccaaaactttta-aaactgcacggggaattag--ggaag--agccgatgaatgatgatttgatgaaggaggtgatgacATGTACGTGAGATTTGAGGTTCCTGAGGACATGCAGAACGAAGCTCTGAGTCTGCTGGAGAAGGTTAGGGAGAGCGGTAAGGTA 284

Arcve_0453 aa-atatatact------------ctgccctcggctcaccaaaagattta-aaacgtaaccgccttgatg--tgtga--agccgatgagtgatgacgcaaggaaggaggtgtaaagATGTACGTGAGGTTTGAGGTTCCTGAGGATTTGCAGAATGAAGCTCTCTCCTTGCTTGAAAAGGTGAGGGAGACGGGGAAAGTG 284

Aboo_1236 gt-ctactgattt-----------tccaaaattatttat------------acaataagccttttcgtgctttgcctatgattgattaaatgaaggaggtaaagaaaATGGCTGCAATATATGTAAGGTTTGAGACACCAAAAGAACTGCAGGAAAAAATGCTTAGTGCGGTGGAGATTTCCAAAGAAACTGGAAAAGTG 293

HQ2885A ---------------------------------------------------ggaccgat-tcgccggcgtggcag----tcgagcccgcgagcagggatggagaataccaATGCCAGTTTATGTAGAATTTGATGTACCGGCTGACCTTGCAGATGATGCTCTTGAAGCACTCGAGGTCGCTCGGGACACGGGAACCGTA 290

VNG1157G -----------------------------------------------gcggccggacgg-tctttactcggctaagagcgcaagcccgtccgcgggataggtgaacacaaATGCCAGTATACGTAGACTACGACGTTCCAGCAGACCTCCAGGAGCGAGCCCTCGAATCCCTTGAGGTCGCTCGCGACACCGGCAGCGTG 290

OE2662F -----------------------------------------------gcggccggacgg-tctttactcggctaagagcgcaagcccgtccgcgggataggtgaacacaaATGCCAGTATACGTAGACTACGACGTTCCAGCAGACCTCCAGGAGCGAGCCCTCGAATCCCTTGAGGTCGCTCGCGACACCGGCAGCGTG 290

Hlac_1842 -----------------------------------------------gatagggaccga-ccacgggacggacacgcgagcccgca---cggaggataggtgaacaacatATGCCCGTTTACGTAGACTACGAAACCCCAGCCGACCTCGCCGAGCGATCGCTTGAGGCGCTCGAGGTCGCCCGAGACACCGGTACCGTG 290

Halar_2173 ----------------------------------------------cgccagggacgcg-gttccggcgaggcggtcgagcttgcgaacagggttgaggtgactacaaaaATGCCAGTTTACGTCGATTACGAAACCCCTGCCGACCTCGCCGACCGCGCGCTTGACGCGCTTGAGGTCGCCCGGGACACCGGTACCGTA 290

HVO_2737 at-atcgttgaccgg-------------------tc--gcgaccg-accgaccggtgtg-ataccggcgagggagtcgagcccgcgagca--ggatataggtgaacaacaATGGCTGTCTACGTAGACTTCGACGTACCGGCCGACCTCGCTGACAGCGCCGTCGAGGCGCTCGAGGTCGCCCGAGATACTGGTATCGTG 290

Hbor_12060 gg-aacgaccgcga----------------------------------ccagcagaggt-ccgccggcgaggatgtcgagcccgcgagca--g--gaatagGTGAACAACATGGCAGTATACGTAGAATTCGACGTCCCAGCCGACCTCGCAGAAGACGCCCTTGAGGCGCTCGAGGTCGCACGAGACACAGGCACGGTA 299

HacjB3_08675 gt-ctacagcctgcgcggg--------------gtcatgaggctcctagccgtctagga-tctatcaggcaggggagcgagagcccgcg----tgcaggaggagatcacaATGCCAGTATACGTCAACTTCGACGTCCCGGCCGACCTTCAAGAGGACGCCCTCGAGGCCCTCGAGGTCGCCCGAGACACAGGTTCGGTA 290

Nmag_0451 aa-ttcacacctaattcga--------------ttctgggaacggaacacaggccgaac-cctcgagggcaggggagcgcgagcccacgtgtaggagaggtgaacaaccaATGTCAGTCTACGTCACAACCGACATCCCAGCAGACCTCGCAGATGACGCCCTTGAGGCGCTCGAGGTCGCACGAGACACCGGACGAGTA 290

Halxa_0717 ga-ctcccgccgatt------------------ttctatcggcccgggtaccc--agtt-cccggacggcaggggagcgcgagcccacgcgtaggagaggtgaacaacctATGTCAGTCTACGTCAACACCGACATCCCAGCCGACCTCGCCGACGACGCCCTCGAGGCGCTCGAGGTCGCACGAGACACCGGACGCGTA 290

Htur_2527 ga-cttccgccgaag------------------ctacttcggccaggggtgcccagtgc-cccgaatggcaggggagcgcgagcccacgtgtaggagaggtgaacaaccaATGTCAGTCTACGTCAACACCGACATCCCAGCCGACCTCGCAGAGGACGCCCTCGAGGCCCTCGAGGTCGCACGAGACACCGGACGCGTA 290

NP3660A ga-atcgacacgtccgg----------ttcg--ggcgtgagaaccaggtcacaggcggcccggttcgacggcgggggagcgc-gagcccgcgcgtaggaggagttcaacaATGTCAGTATATGTAGACTTCGACGTGCCGGCCGACCTCGAAGACGACGCCCTTGAGGCGCTCGAGGTCGCCCGGGACACCGGCTCAGTA 290

Huta_0750 ga-ctttcgcgccgcctcgatctccggggcgacgccgggatctgcccgtccggggacggtcccgcacggcgggggagtgtga-gcccacgtgcaggaggtgatccaacccATGCCAGTATACGTCGATTTCGATGTTCCCGCGGACCTCGAAGATGACGCCCTCGAGGCGCTCGAGGTCGCGCGAGACACAGGCCGAGTA 290

Hmuk_2621 gg-actctcaggcgggg---------------------------------------------------------gagcgcga-gcccgcgtgcag-gaggtgatccatcaATGCCAGTATACGTAGATTTCGACGTCCCTGCAGACCTCGCAGACGACGCCCTCGAGGCGCTCGAGGTCGCGCGGGACACAGGAAGTGTC 290

HAH_0858 gg-atacacaccatcacgg----------------------tgtgacgggccccgcggcccggacacggcgggggagcctga-gctcgcgcgcaggaggtgaacatacaaATGCCAGTATACGTAGATTTCGACGTTCCCGCGGACCTCGAAGACGACGCCCTCGAGGCGCTCGAGGTCGCCCGGGACACAGGCGCTGTA 290

rrnAC0103 gg-atacacaccatcacgg----------------------tgtgacgggcctcgcggcccggacacggcgggggagcctga-gctcgcgcgcaggaggtgaacatacaaATGCCAGTATACGTAGATTTCGACGTTCCCGCGGACCTCGAAGACGACGCCCTTGAGGCGCTCGAGGTTGCCCGGGACACAGGCGCAGTA 290

MK1598 aa-actttatttatt---------tcaaccgagcgtcggacgccgga----cgaggagatcgaccacgaggatgaggatgaaccgatgagtggggtgatggttaATGTCCAAGCCCATGTACGTGAAGTTCGAGGTCCCGGAGGAGCTGGCCGAGAAGGCCTACGAGGCCCTCGAGATCGCTCGGGACACCGGTCGTATC 296 TSS verified by RNA-Seq data

TERMP_01118 ca-aacagcgtctgt---------tagggaaaggtttaaaaactaaattctcctttgctatcttgacagctcgATGAACGATGAAG-TTTTGGAGGGATGTGAAATGGCAAAGCCAAGTTACGTAAAATTTGAGGTTCCAAAAGAGCTGGCTGAGAAAGCCCTTGAGGCTGTTGAGATTGCAAGAGACACTGGAAGAATA 326

TSIB_0419 -t-ttctctcatttc---------aaaaggaaagatttaaaaagcaagcatccttccctaacttgatgactcgatgaacgatgaa--gtTTGGAGGGATGTGAAATGGCAAAGCCAAGTTATGTAAAATTTGAAGTTCCAAAGGAACTTGCTGAGAAAGCCCTTGAGGCTGTTGAGATTGCAAGAGACACCGGAAAGGTA 311

GQS_00200 ta-aatcgtttcct----------atcgaaatccttttaaagcctccccc-agaatcgagcatgcacaactcgatgaacgatgaggattccggagggatgaaaaATGGCTAAGCCAAGCTACGTCAAGTTTGAGGTTCCGCAGGAGCTCGCTGAGAAGGCTCTTGAGGCCGTTGAGCTCGCTCGCGACACCGGAAGGATA 296

TON_0954 tt-tcccgccggct----------atcgaaatccttttaaaccatcgccc-agaattgagcacgcaaaactcgatgaacgatgaagttt-cggagggatgaaggATGGCGAAGCCAAGCTACGTCAAGTTTGAAGTTCCGGCAGAGCTTGCTGAGAAGGCCCTTGAGGCTGTCGAGCTTGCTCGCGACACCGGAAGGATA 296

TGAM_1876 at-gaaagatcact----------gaaggaaaggtttaaaaacgcaatccgtctttctaccttgacaaaccggatgaacgatgagg-tttcggagggatgaaagATGGCGAAGCCGAGCTACGTAAAGTTTGAGGTTCCCCAGGAGCTTGCCGAGAAGGCCCTTGAGGCCGTTGAGATCGCTCGCGACACCGGAAGGATA 296

TK1311 tg-gccagttgagt----------agcgaaaccctttt---aagcggtcagcccgagtcggtggtgcaaacagatgaacgatgagg-tttcggagg-gATGAAGATGGCGAAGCCGAGCTACGTGAAGTTTGAGGTTCCACAGGAGCTTGCCGAGAAGGCCCTTGAGGCCGTTGAGATCGCAAGGGACACCGGAAGGATA 302

PFC_06055 gt-atcctgcatcta---------accaaaatccttttaa------agcagagttttcaaccaacctcgacagatgaacgatgact-aa-aggagggatggatgATGGCCAAACCAAGTTACGTGAAGTTTGAAGTCCCAAAGGAGCTTGCTGAGAAGGCTCTACAAGCAGTAGAAATAGCCAGAGACACAGGAAAGATT 296

PF1367 gt-atcctgcatcta---------accaaaatccttttaa------agcagagttttcaaccaacctcgacagatgaacgatgact-aa-aggagggatggATGATGGCCAAACCAAGTTACGTGAAGTTTGAAGTCCCAAAGGAGCTTGCTGAGAAGGCTCTACAAGCAGTAGAAATAGCCAGAGACACAGGAAAGATT 299

PYCH_03310 aa-aacgtttccagg---------accacaagccttttaa------attcacatcctcaaccaagctcgacagatgaacgatgacg-ta-aggagggatagaagATGGCGAAGCCGAGCTATGTGAAGTTTGAGGTTCCCAAGGAGCTCGCCGAGAAGGCCCTCGAGGCTGTCGAGATTGCCAGGGACACCGGAAGGATA 296

PNA2_0082 cc-ttcaattttgtg---------accacaacccttttaa------attctcccattcaaccaagctcgacagatgaacgatgact-aacgggagggatggATGATGGCGAAGCCAAGCTATGTGAAGTTTGAAGTTCCAAAGGAGCTCGCTGAAAAGGCTCTTCAGGCTGTAGAGATAGCAAGAGATACAGGGAAGATA 299

PAB0460 aa-acccttttccac---------accataacccttttaa------ataccttggagcaaccaagctcgacagatgaacgatgact-gA-TGGAGGGATGGATGATGGCGAAGCCAAGCTATGTGAAGTTTGAGGTTCCAAAGGAGCTCGCTGAGAAGGCTCTCCAGGCAGTTGAGATAGCTAGGGACACAGGAAAGATA 311

PH1496 aa-atacttttcttc---------accataagtcttttaa------atcttctcaattaaccaagctcgacagatgaacgatgact-aaagggagggatggATGATGGCGAAGCCAAGCTACGTTAAGTTTGAAGTTCCAAAGGAACTCGCTGAGAAGGCTCTTCAAGCAGTTGAGATAGCTAGGGATACAGGAAAGATA 299

Metin_1393 AGAAAAGGAGCTAATGAGGTTACTAAAGCAGTTGAGAGGAAACAGGCTAAGCTTGTTATCATAGCTGAAGATGTCAAGCCAGAGGAGATAGTTGCTCACCTTCCAGTTTTATGTGAAGAAAAAGGAATTCCTTATGCTTATGTAGCTTCAAAACAAGATTTAGGAAAGGCTGCAGGAATTGAAGTTGCTGCCTCTTCAGT 481

Mefer_1103 AAAAAAGGAGCTAACGAAGTTACAAAGGCAGTTGAAAGAGGTATTGCAAAATTAGTTATTATCGCTGAGGATGTTCAACCAGAAGAAGTTGTTGCTCACCTTCCATACTTATGTGAAGAAAAAGGAATTCCTTACGCTTACGTAGCTTCAAAACAAGATTTAGGTAAAGCTGCTGGATTAGAAGTTGCTGCATCATCAGT 481

Metvu_1339 AAAAAAGGAGCTAACGAAGTTACAAAGGCAGTTGAAAGAGGAATTGCAAAATTAGTAATCATCGCTGAAGATGTTAAACCAGAAGAAGTCGTTGCTCATCTTCCATACTTATGTGAAGAGAAAGGAATTCCATACGCTTATGTGGCTTCAAAACAGGACTTAGGAAAAGCTGCTGGGTTGGAAGTTGCTACATCAGCAGT 481

MFS40622_0835 AAAAAAGGAGCTAACGAAGTTACAAAGGCAGTTGAAAGAGGTATCGCAAAATTAGTTATCATTGCTGAAGATGTTAAACCAGAAGAAGTTGTTGCTCATCTCCCATACTTATGTGAAGAGAAAGGAATTCCTTATGCTTACGTAGCTTCAAAACAGGACTTAGGTAAAGCTGCTGGATTAGAAGTTGCTGCATCATCAGT 481

MJ1203 AAAAAAGGAGCTAACGAAGTTACAAAGGCAGTTGAAAGAGGTATCGCAAAATTAGTTATCATTGCTGAAGATGTTAAACCAGAAGAAGTTGTTGCTCACCTCCCATACTTATGTGAAGAGAAAGGAATTCCTTACGCTTACGTAGCTTCAAAGCAGGATTTAGGTAAGGCTGCTGGATTGGAAGTTGCTGCATCATCAGT 481

Metig_1662 AAAAAAGGAGCAAACGAAGTTACAAAAGCAGTTGAAAAAGGAATTGCTAAACTCGTTATAATTGCTGAAGATGTTCAACCAGAAGAAATCGTTGCTCACCTTCCACCATTATGTGAAGAAAAAGGAATCCCATACACATACGTAGCATCAAAACAAGAATTAGGAAAAGCAGCAGGTTTAGAAGTTGCAGCATCATCAGT 481

Maeo_0081 AAAAAAGGAGCAAACGAAGTAACAAAAGCAGTAGAAAGAGGAACTGCAAAATTAGTAGTTTTAGCAAAAGATGTTCAACCAGAAGAAATTGTTGCGCACATACCTATAATCTGTGAAGAAAAAGGTATCCCATACACATACATAGCAACAAAAGAAGATTTAGGTAAAGCTATTGGATTAGAAGTTTCAACAGCAGCAGT 481

Metok_1174 AAAAAAGGAGCAAATGAAGTAACAAAAGCAGTAGAAAGAGGAACTGCTAAATTAGTAGTTTTGGCAAAAGATGTCCAACCAGAAGAAATCGTTGCTCATATACCTGTAATTTGCGAAGAAAAAGGTATTCCTTACTCATATGTAGCTACAAAAGAAGATTTGGGAAAAGCCATTGGCTTAGAAGTTCCAACATCAGCCGT 481

Mvol_0393 AAAAAAGGAGCTAACGAAGTAACCAAAGCAGTAGAAAGAAGTAGAGCAAAATTAGTAGTAGTTGCTAAAGATGTTCAACCTGAAGAAATCGTAGCACACATCCCTGCTATCTGTGAAGAAAAAGGTATCGCATACACATATTGCGCTACAAAAGAAGATTTAGGAAAAGCTGCAAACTTAGAAGTTCCTACATCAGCAAT 481

Mevan_1553 AAAAAAGGCGCAAACGAAGTTACAAAAGCAGTTGAAAAAGGAATTGCAAAATTAGTTGTAGTTGCAAAAGATGTACAACCTGAAGAAATCGTTGCACACATTCCTGTAATCTGTGAAGAAAAAGGAATTGCATACTCATACTGTTCAACAAAAGAAGCTTTAGGAAAAGCAGCAGGTTTAGAAGTACCTACATCAGCTAT 538

MmarC5_0963 AAAAAAGGCGCTAACGAAGTTACAAAAGCAGTTGAAAAAGGAATTGCAAAATTAGTTGTAATCGCACAAGATGTACAACCTGAAGAAATCGTTGCACACATTCCAGTAATCTGCGACGAAAAAGGAATTGCATACTCATACAGTTCAACAAAAGAAGCTTTAGGAAAAGCTGCTGGTTTAGAAGTACCTACATCAGCTAT 538

GYY_03255 AAAAAAGGCGCTAACGAAGTTACAAAAGCAGTTGAAAAAGGAATTGCAAAATTAGTTGTAATTGCACAAGATGTACAACCTGAAGAAATCGTTGCACACATTCCAGTAATTTGCGACGAAAAAGGAATTGCATACTCATACAGTTCAACAAAAGAAGCTTTAGGAAAAGCTGCTGGTTTAGAAGTACCTACATCAGCTAT 481

MMP0641 AAAAAAGGCGCTAACGAAGTTACAAAAGCAGTAGAAAAAGGAATTGCAAAATTAGTTGTTATCGCACAAGATGTACAACCTGAAGAAATCGTTGCACACATCCCAGTAATCTGCGACGAAAAAGGAATTGCATACTCATACAGTTCAACAAAAGAAGCTTTAGGAAAAGCTGCTGGTTTAGAAGTACCTACATCAGCTAT 481

MmarC6_0248 AAAAAAGGCGCTAACGAAGTTACAAAAGCAGTTGAAAAAGGAATTGCAAAATTAGTTGTAATCGCACAAGATGTACAACCTGAAGAAATCGTTGCACACATTCCAGTAATCTGTGACGAAAAAGGAATTGCATACTCATACAGTTCGACAAAAGAAGCTTTAGGAAAAGCTGCTGGTTTAGAAGTACCTACATCAGCTAT 538

MmarC7_1665 AAAAAAGGCGCTAACGAAGTTACAAAAGCAGTAGAAAAAGGAATTGCAAAATTAGTTGTAATCGCACAAGATGTACAACCTGAAGAAATCGTTGCACACATTCCAGTAATCTGCGACGAAAAAGGAATTGCATACTCATACAGTTCAACAAAAGAAGCTTTAGGAAAAGCTGCTGGTTTAGAAGTACCTACATCAGCTAT 538

Mlab_1497 AAGAAAGGCGCAAACGAGGCAACCAAGGCCGTCGAGCGTGGAATTGCAGCTCTCGTCTTAATCGGCGCAGACGTTGCACCTGAAGAAATCGTCATGCACATCCCCGGACTTGCAGACGAGAAGGAAATTCCGTTCGTTTTCATCAACAAGCAGGCAGACATCGGAGCCGCATGCGGTCTTGACGTCGGATGTACTGCAGT 493

Mpet_2104 AAAAAAGGATCGAACGAAGTAACAAAAGCTGTTGAAAGAGGAGTCGCCCAGCTCGTCCTTATCGGCGGAGACGTCGAACCTGAGGAGATTGTAATGCACATCCCGGCACTCTGCGACGAGAAGCAGTGTGCATATGTTATTATCAACCAGCAGAACGATGTCGGAGCAGCAAGCGGTCTTGACGTAGGATCGGCAGCAGC 493

Mhun_1601 AAAAAGGGCTCCAACGAAGCCACCAAGGCCATCGAGCGCGGGATCGCACAGCTGGTCCTTATTGGCGGAGATGTTGAACCAGCAGAGATCGTCATGCATCTTGGACCACTCTGCGAAGAAAAGAAGATTCCGTATCTCTTCATCAGCAAGCAGAATGATATCGGCGCTGCTTGCGGACTGGAAGTCGGCTCTGCTGCTGC 490

Memar_0721 AAGAAGGGGTCCAACGAGGCAACAAAAGCAGTCGAGCGGAACATTGCCCAGCTGGTTCTCATCGGTTCGGACGTTGAGCCTGAAGAAATCGTGATGCATCTGCCGCCGCTCTGCGAGGAGAAGCAGATCCCGTTCGTCTACATCTCCAAGCAGAACGACATCGGGCTGGCGAGCGGCCTCGAGGTCGGCTCGGCAGCCGC 493

Mboo_0690 AAGAAGGGTTCAAACGAGGCAACAAAAGCCATTGAACGCAGCGCCGCACTGCTTGTTTTAATCGGCGCCGATGTGGAGCCCGCAGAGATTGTTATGCACCTGGCCCCCTTATGCGAAGAAAAGAAGATCCCCTACGTCTTCATCAACAAACAAAATGACATCGGTGCCGCAAGCGGTCTTGATGTCGGTTCTGCCGCGGC 493

Mpal_0523 AAGAAAGGGTCCAATGAAGCGACAAAAGCTATCGAACGATCCATTGCTCAGCTGGTCCTGATCGGCGCAGATGTGGAGCCAGAAGAGATTGTGATGCATCTGGCCCCACTCTGTGATGAGAAGCACATCCCGTACATCTTTATCGGGAAGCAGAACGATATCGGAGCCGCCAGCGGCCTGACTGTTGCCTCGACTGCTGC 493

MCP_1041 AGAAAGGGCACCAACGAGGTCACCAAGGCCGTCGAGAGGGGCGTAGCCCAGCTCGTGATCATCGGCGAGGACGTGCAGCCCGAGGAGATCGTTGCCCACATTCCCGCGCTATCTGACGAAAAGAAGATACCCTACATATTCATTAAGAAGCAGGAAGACATCGGCGCCGCATCAGGACTCGAGGTCGGCTGCGCCGCATC 496

LRC145 AGGAAAGGCACCAACGAGGTCACCAAGGCCATCGAGAGGGGCGTTGCTCAGCTCGTCATCGTCGGCGAAGATGTCACCCCCGAGGAGATCGTCGCTCACATCCCGGCACTGTCCGACGAGAAGAAGATCCCGTACGTCTTTGTAAAGAAGCAGCAGGAACTCGGCGCCGCATCCGGCCTCGAAGTAGGCTGTGCGACCTC 496

MCON_1706 AAGAAGGGCACAAACGAGGCCACCAAGGCCATAGAGAGGGGTGTGGCCCGTCTGGTCATAGTAGGCGAGGATGTTCAGCCACCTGAGATCGTCGCTCATATTCCCGCTCTATGCGAAGAGAAGAAGACCCCCTATATCTACGTCAAGAAACAGAGCGAGCTTGGTGCCGCTGCAGGCCTGGGCGTTAAGAGCGCAGCCGC 496

Mthe_0331 AAGAAGGGAACGAACGAAGCTACCAAGGCCGTGGAGCGAGGGGTTGCGAAGCTCGTGATAATTGGAGAGGATGTGGAGCCACCTGAGATAGTGGCACATCTGCCCCCGCTCTGCGAGGAGAAGAACACCCCCTACGTGTACGTGAAGAAACAGAGCGACGTGGGGGCGGCTGCCGGTCTGAGTGTGAAGAGCGCGGCAGC 496

Mbar_A3388 AAAAAGGGCACCAATGAAGCCACAAAAGCAATCGAAAGAGGCAATGCGAAGCTCGTCCTTATTGCAGAAGATATCGAGCCTGCCGAAATTATTGCTCACATAGCTCCACTTTCCGAAGAAAAGAAAGCACCTTACATCTTCATCAAAAACCAGAAGGAACTCGGTGCAGCCAGTGGGCTTGGTGTGTCCTGCGCAACTGT 490

MA1521 AAAAAGGGCACAAACGAAGCCACAAAAGCAATCGAAAGAGGCAATGCAAAGCTTGTCCTGATTGCCGAAGACATTGAGCCTGCAGAGATCGTCGCTCACATAGGCCCTCTTTCCGAAGAAAAGAAAGCTCCCTACATCTTCATCAAGAACCAGAAAGAACTTGGTGCAGCCAGCGGACTTGGAGTCTCCTGTGCAACCGT 490

MM_2467 AAAAAGGGCACAAACGAAGCCACAAAAGCAATCGAAAGAGGCAATGCAAAGCTTGTCCTGATTGCCGAAGATATCGAGCCTGCAGAGATTGTCGCTCACATCGGCCCTCTTTCCGAAGAAAAGAAAGCTCCCTATATCTACATCAAGAACCAGAAAGACCTCGGCGCAGCCAGCGGACTTGGAGTCTCCTGTGCAACCGT 490

Mzhil_1258 AAGAAAGGAACAAATGAAGCAACAAAAGCAATTGAAAGAGGGGTTACAAAACTTACTATCATTGCAGAGGACGTAAGTCCTGAAGAAATTATTGCTCATATACCGGTCCTTTGTGAAGAAAAAAATACACCATACATATTTGTAAAAGAACAAAAGGAACTGGGGGCAGCCTGCGGTATTGGTGTGGCATGTGCTGCTGT 484

Metev_1406 AAAAAAGGAACAAATGAGTCCACGAAAGCAGTGGAGAGAGGTACATCTAAACTTGCAGTGATTGCTGAAGATATAGACCCTGAAGAAATAATTGCTCACCTACCACCACTGTGCGAAGAGAAAAGCATTCCATACATATTCGTATCCCAGCAAAAAGAACTTGGTTCAGCATGTGGACTTGAAGTTGGCTGTTCTTCCGT 481

Mbur_0219 AAGAAAGGTACGAACGAAGCAACAAAAGCTATCGAAAGAGGCATCACAAAGCTTGCAGTAATCGCAGCTGATATTGAGCCATCCGAGATCGTTGCTCACATTCCAGCTCTTTGCGAAGAAAAGAACACACCATACATTTTTGTAAAACAGCAGAAAGAACTTGGTGCAGCATGTGGAATTGGTGTAGGCTGTGCAGCTGT 481

Mmah_0863 AAAAAAGGAGCTAACGAAGCTACAAAGGCTGTAGAAAGAGGTATTGCAAAACTGGCAGTCATTGCAGACGATGTTGAACCTGCAGAAGTAGTAGCTCACATTGGTCCTCTTTGTGAAGAAAAGAATGCACCTTACATCTATGTAAAACAGCAGAAAGAACTCGGAGCTGCCTGTGGAATAGGCGTTGGCTGTGCTGCAGT 481

Mfer_0452 GTCAAAGGAACAAATGAAACAACAAAAGCCGTTGAGAGAGGTATAGCAAAATTAGTAATTATAGCTGAAAATGTAGACCCACCAGAAATTGTAGCACACTTACCTGTTCTTTCAGAAGAAAAAGAAATACCATATATATACGTACCAAAGAAAGAAGAATTAGGAGCTGCTGCCGGTATAAATGTATCAGCTGCATCTGC 496

MTBMA_c07050 TCAAAGGGTACAAACGAGGTTACCAAGGCAGTTGAAAGGGGAGTCGCACAGCTCGTACTTGTAGCTGAGGATGTTGAACCAGCCGAAATCGTGGCTCACCTCCCACTCCTTGCAGAGGAAAAGGAAATACCATACATATACCTTCCAACCAAGGATGAACTGGGTGCAGCCGCAGGTCTCAACGTCGGCACAGCATCAGC 496

MTH255 TCCAAGGGTACAAATGAGGTTACCAAGGCTGTCGAGAGGGGAGTTGCTCAGCTCGTATTAATAGCTGAGGACGTTGAACCTGCAGAGATCGTTGCTCACCTTCCACTCCTCGCAGAGGAAAAGGAAATACCATACATATACATCCCAACAAAGGATGAACTGGGTGCAGCCGCAGGTCTGAACGTTGGAACAGCATCATC 517

mru_1490 GCAAAAGGAACTAACGAAGTAACCAAATTTATCGAAAGAGGAAACGCAGCTTTAGTCGTTATCGCAGAAGATGTAGATCCTGCTGAAATCGTTGCTCACATTCCTGTTCTTGCAGAAGAAAAAGAAATTCCTTACGTATACTTAGCTACCAAAGATAAAGTTGGTGCAGCTGCTGGTTTAAGTGTTGGAACTGCTTCCGC 532

Msm_0206 GCAAAAGGAACTAACGAAGTAACTAAATTTATCGAAAGAGGAGACGCAGCACTTGTTGTTATCGCAGAAGATGTTGATCCTGCTGAAATTGTTGCACACATCCCTGTTCTCGCTGATGAAAAAGAAATTCCTTACATTTACTTACCTACTAAAGAACAAGTTGGTGGAGCTGCAGGTTTAACTGTCGGTACCGCATCCGC 496

MSWAN_1751 GGTAAAGGAACTAACGAGGTAACTAAAGCCATAGAAAGAGGCAACGCACTACTCGTATTCATAGCTGAAGATATTCAGCCACCAGAAATAGTAGCTCATTTACCAGTACTTGCTGAAGAAAAAGAGATTCCTTACGTTTACATTGCAACAAAAGACGAGTTAGGAGAAGCAGCAGGACTTAACGTTGGTACAGCATCTGC 496

Msp_0633 GGAAAAGGAACAAACGAAGTAACAAAAAACATCGAAAGAAACAATGTTGCTTTAGCAGTAATTGCAGAAGATATTGAACCTGCAGAAATTGTAGCACACTTACCTATTTTAGCAGAAGAAAAAGAAATTCCTTACGTATATCTCCCAACCAAAGAAGAATTAGGAGAAGCTGCAGGACTTAATGTTGGTACAGCATCTGC 496

Faci_050500008291 AAGAAAGGGACAAATGAAGTAGTTAAGTCTATTGAGAGAGGAGAAGCAAAGGTAGTTGTAATAGCAGAGGATGTTTCACCCCCTGAAGTTGTTTTTTACATACCGGTCCTTTGTGAGGAAAGGAAAGTCCCGTATACATATGTAAAAAATAAGAGTGACCTTGGACTGAAAGTTGGTATTGCATCTGCAGCATCAATAGC 493

PTO1280 AGAAAGGGTACCAATGAGGTAGTAAAATCAATAGAGAGGGGAGAATCAAAGCTTGTTGTTATAGCCGAGGATGTATCACCTGCAGAGGTTGTTTACTATCTTCCAACATTATGTGAGGAAAGAAAGGTACCATATGTATATGTAAAAAAGAAATCCGATCTTGGACTCAAGGTTGGCATTGCATCTGCGGCATCGGTATC 490

Ta1116 AAGAAGGGTACTAACGAGGTTATAAAGTCTATAGAGAGGGGCGAAAGCAAGCTTGTGGTTATTGCCGAAGACGTTAACCCGCCTGAGGTTGTGTACTACCTGCCATCCCTCTGCGAGGACAAGAAAGTTCCGTACGTCTACGTAAAGAAGAAGGCGGATCTTGGCTCAAAGGTCGGCATAGCCTCTGCAGCTTCTGTATC 493

TVN0449 AAGAAAGGTACAAACGAAGTAATAAAGTCGATAGAACGCGGGGAAAGTAAGCTGGTAGTCATAGCAGAGGATGTAAACCCACCTGAGGTTGTGTACTATTTGCCTTCCCTATGTGAGGATAAGAAAGTACCTTATGTATACGTCAAAAAGAAGGCAGACCTTGGATCAAAAGTTGGAATTGCATCTGCAGCATCGGTGTC 493

Arcpr_0494 AAGAAAGGTACAAACGAAACTACAAAAGCCGTCGAAAGAGGTTTGGCTAAGCTGGTTTACATAGCCATGGATGTCGATCCACCAGAAATCGTAGCTCACCTACCTCTGCTCTGCGAGGAGAAGAACATACCATACGTCTACGTAAAGAGCAAAGCTGATTTGGGTAAAGCTGCCGGAATAGAAGTTCCTGCTGCATCCGC 487

Ferp_0621 AAGAAGGGAACGAACGAGACGACGAAAGCCGTAGAAAGGGGTTTGGCGAAGCTCGTTTACATAGCTATGGACGTAGATCCTCCGGAGATCGTTGCTCATTTGCCTTTGCTCTGCGAAGAAAAGAACATACCCTATGTTTACGTGAAGTCCAAAGAGGATCTTGGAAAAGCGGCTGGAATCGACGTTGCGGCAGCTTCAGC 490

AF0764 AAGAAAGGTACCAACGAGACGACAAAGGCTGTGGAGAGGGGACTGGCAAAGCTCGTTTACATCGCAGAGGATGTTGACCCGCCTGAGATCGTTGCTCATCTGCCCCTCCTCTGCGAGGAGAAGAATGTGCCGTACATTTACGTTAAAAGCAAGAACGACCTTGGAAGGGCTGTGGGCATTGAGGTGCCATGCGCTTCGGC 484

Arcve_0453 AAAAAAGGGACAAACGAGACGACAAAAGCAGTGGAACGAGGTATGGCGAAGCTTGTATACATAGCCACAGACGTTGACCCGCCGGAAATCGTAGCACATCTGCCACTGCTCTGCGAGGAGAAGAACGTACCATACATTTACATCAACAGCAAGAGCACCCTTGGCCAGGCTGTCGGAATTGAGGTTGACTGTTCCGCAGC 484

Aboo_1236 AGGAAGGGAACGAACGAAGTCACAAAAACGATTGAGAGAGGAGATGCAAAATTTGTCATAATTGCTGAGGATGTAAATCCTCCAGAGATTGTGGCTCATCTGCCTCTTCTATGCGAAGAGAAGGGAATCCCATATGGCTATGTTGCTACTAAGGAAGAGCTCGGCAAAAGAGTTGGAATAAAGAGTGCTGCCTCGGTA-- 491

HQ2885A AAGAAAGGAACGAATGAAACAACCAAAGCTATCGAACGTGGGACTGCTGAACTTGTCCTGGTTGCTGAAGATGTCTCTCCTGAAGAAATTGTGATGCATCTTCCAGAACTCGCCGATGAAAAAGGCATTACGCTCGCCTTCGTTGAAACGCAAGACGAGATTGGACAAGCAGCAGGACTTGAAGTGGGCTCTGCTGCTGC 490

VNG1157G AAGAAAGGTACCAACGAGACGACGAAAGCGATCGAGCGCGGCAACGCCGACATCGTGTTCGTCGCCGAGGACGTCTCCCCCGAGGAGATCGTCATGCACCTCCCGGAGCTCGCCGCAGAGAAAGGCATCGAGGTCGTCTTCGTGGAGACACAGGACGAACTCGGGAACGCCGCCGGCCTCGAAGTTGGCTCCGCGGCCGC 490

OE2662F AAGAAAGGTACCAACGAGACGACGAAAGCGATCGAGCGCGGCAACGCCGACATCGTGTTCGTCGCCGAGGACGTCTCCCCCGAGGAGATCGTCATGCACCTCCCGGAGCTCGCCGCAGAGAAAGGCATCGAGGTCGTCTTCGTGGAGACACAGGACGAACTCGGGAACGCCGCCGGCCTCGAAGTTGGCTCCGCGGCCGC 490

Hlac_1842 AAGAAAGGAACCAACGAGACCACGAAGGCCGTCGAGCGCGGCAACGCCGACCTCGTCATCGTCGCCGAGGACGTCTCTCCCGAAGAGATCGTGATGCACCTCCCCGAGCTTGCCGAGGAGAAGGGCATCCCGGTCGTCTTCGTCGACACGCAGGATGAAGTCGGCCACGCCGCCGGCCTCGAAGTCGGCTCGGCCGCCGC 490

Halar_2173 AAGAAAGGAACCAACGAGACCACCAAGGCCGTCGAGCGTGGCAACGCCTCGCTTGTCTTCGTGGCCGAAGACGTCCAGCCCGAAGAGATCGTGATGCATCTCCCCGAACTCGCCGAGGAGAAGGGTATCCCGGTTGTCTTCGTCGAAACCCAGGACGACCTGGGTCACGCAGCCGGCCTCGAGGTCGGCTCCGCCGCCGC 490

HVO_2737 AAGAAAGGAACCAACGAAACGACCAAGTCCATCGAGCGGGGCAACGCCCAGCTCGTCCTCATCGCGGAAGACGTCTCCCCCGAGGAGATCGTCATGCACCTGCCCGAACTCGCCGACGAGAAGGGCATTCCGTTCATCTTCGTCGAGACGCAGGACGACATCGGCCACGCCGCCGGCCTCGAAGTCGGCAGTGCCGCGGC 490

Hbor_12060 AAGAAAGGTACGAACGAGACGACCAAAGCGGTCGAGCGCGGCAACGCCGAGCTCGTCCTCGTCGCCGAAGACGTCTCCCCCGAGGAGATTGTGATGCACCTCCCCGAAATCGCAGACGAGAAGGGCATCCCGTACATCTTCATCGAGACGCAGGACGAAGTCGGTCACGCTGCGGGTCTCGAAGTCGGCTCCGCTGCTGC 499

HacjB3_08675 AAGAAAGGTACCAACGAAACGACCAAAGCGATCGAGCGTGGCAACGCATCTCTCGTCGTCATCGCCGAGGACGTCCAGCCCGAGGAGATCGTGCTGCACCTCCCCGAGCTCGCCGACGAGAAGGGTATCCCCTTCGTTTTCGTCGAGACTCAGGACGACGTCGGCCACGCCGCGGGCCTCGAAGTCGGCTCGGCGGCCGC 490

Nmag_0451 AAGAAAGGAACGAACGAAACCACGAAGGCGATCGAGCGCGGCAACGCCGAGCTCGTTTTCGTCGCTGAAGACGTCTCCCCCGAGGAGATCGTCATGCACCTCCCAGAACTCGCCGACGAGAAGGGTATTCCTGTCGTCTTTATCGAGACCCAGGACGACGTCGGCCACGCTGCAGGCCTCGAGGTTGGCTCGGCCGCTGC 490

Halxa_0717 AAGAAAGGAACCAACGAAACGACGAAGGCCATCGAGCGCGGCAACGCCGAGCTCGTCTACGTCGCCGAAGACGTCTCCCCCGAGGAGATCGTGATGCACCTGCCGGACCTCGCCGACGAGAAGGGCATCCCCGTCGTCTTCATCGAGACCCAGGACGACGTCGGCCACGCCGCCGGCCTCGAGGTCGGCTCGGCCGCCGC 490

Htur_2527 AAGAAAGGAACGAACGAAACCACGAAGGCGATCGAGCGCGGCAACGCCGAGCTCGTCTACGTCGCCGAGGACGTCTCCCCCGAGGAGATCGTCATGCACCTGCCCGACCTCGCCGAGGAGAAGGGCATCCCCGTCATCTTCGTCGAGACTCAGGACGACGTCGGCCACGCAGCCGGCCTCGAGGTCGGCTCGGCCGCCGC 490

NP3660A AAGAAAGGCACAAACGAGACGACGAAAGCCGTAGAGCGCGGCAACGCGGAACTGGTCTTCGTCGCCGAGGACGTCAGCCCCGAGGAGGTCGTCATGCACCTTCCGGAGATCGCCACCGAAAAGGAGATTCCGTACGTCTTCGTCGGTACACAGGACGACATCGGCCACGCCGCTGGCCTGCAGGTCGGCAGCGCTGCCGC 490

Huta_0750 AAGAAAGGAACCAACGAGACGACCAAGGCGATCGAGCGCGGCAACGCCGAGCTCGTCGTCGTCGCCGAGGACGTTCAGCCGGAGGAGATCGTGATGCACATTCCGGAACTCGCAGCCGAGAAGGACGTCCCCTATCTGTTCGTCGGCGCCCAGGACGATCTCGGTCACGCGGCCGGCCTCGAAGTCGGCTCCGCGGCGGC 490

Hmuk_2621 AAGAAAGGAACGAACGAGACGACCAAGGCAATCGAGCGCGGCAACGCCGCACTCGTCCTCGTCGCCGAGGACGTTCAGCCCGAGGAGATCGTCATGCACATCCCGGAACTCGCCGACGAGAAAGACGTGCCGTTCGTCTTCGTCGCCGAGCAGAGTGACCTCGGCCACGCCGCCGGCCTCCAGGTCGGCAGTGCCGCCGC 490

HAH_0858 AAGAAGGGTACAAACGAAACGACCAAGTCCATCGAGCGCGGCTCCGCCGAGCTCGTCTTCGTCGCCGAAGACGTCCAGCCCGAGGAGATCGTCATGCACATTCCGGAACTCGCCGACGAGAAGGGTGTCCCCTTCATCTTCGTCGAGCAGCAGGACGACCTCGGACACGCCGCCGGGCTCGAAGTCGGCTCGGCCGCCGC 490

rrnAC0103 AAGAAGGGTACAAACGAGACGACCAAGTCCATCGAGCGCGGCTCCGCTGAGCTCGTCTTCGTCGCCGAAGACGTCCAGCCCGAGGAAATCGTCATGCACATTCCGGAGCTTGCCGACGAGAAGGGCGTTCCCTTCATCTTCGTCGAGCAGCAGGACGACCTCGGTCACGCCGCCGGGCTCGAAGTCGGCTCGGCCGCTGC 490

MK1598 CGTAAGGGCACCAACGAGACCACCAAGGCCGTCGAGCGCGAGGAAGCCGTACTCGTGCTGATCGCTGAAGACGTGGACCCCGAGGAAGTCGTCGCTCACCTACCGGAGCTCTGCGATGAGAAGGGCATTCCGTACGTGTACGTCCCGAGCAAGGACGAGCTGGGTGCCGCCGCCGGCATCGACGTAGCCGCAGCCAGCGC 496

TERMP_01118 AGGAAAGGCACAAACGAGACAACAAAAGCCGTTGAAAGGGGACAGGCAAAGCTTGTCATAATCGCAGAAGATGTTGATCCAGAGGAGATTGTCGCACACTTACCACCACTCTGTGAGGAAAAGGAGATTCCATACATCTATGTTCCAAGCAAGAAGGAGCTTGGGGCTGCTGCTGGTATTGAGGTTTCAGCTGCAAGCGT 526

TSIB_0419 AGAAAAGGTACAAACGAGACAACAAAGGCCGTTGAGAGGGGTCAAGCTAAGCTTGTGGTAATTGCCGAGGATGTTGATCCTGAAGAGATTGTTGCTCACTTGCCACCCCTCTGTGAGGAGAAAGAGATACCCTACATTTATGTTTCAAGCAAAAAAGAGCTTGGAGCTGCTGCCGGTATTGAGGTTCCATCTGCAAGCGT 511

GQS_00200 AGGAAGGGTACCAACGAGACGACCAAGGCCGTTGAGAGGGGCCAGGCCAAGCTCGTTGTCATCGCTGAGGACGTTGACCCCGAGGAGATAGTTGCCCACCTCCCGCCGCTGTGCGAGGAGAAGGAGATTCCGTACATTTACGTTCCGAGCAAGAAGGAGCTCGGCGCCGCTGCCGGGCTTGAGGTCCCGTCGGCCAGCAT 496

TON_0954 AGGAAGGGTACCAACGAGACCACCAAGGCCGTTGAGAGGGGCCAGGCTAAGCTCGTTATCATTGCAGAGGATGTTGACCCGGAGGAGATCGTTGCCCACCTCCCACCGCTGTGTGAGGAGAAGGAGATCCCGTACATCTACGTTCCGAGCAAGAAGGAGCTCGGCGCTGCCGCTGGTATTGAGGTTCCAGCTGCCAGCGT 496

TGAM_1876 AGGAAGGGCACCAACGAGACCACCAAGGCCGTCGAGAGGGGCCAGGCCAAGCTCGTTATAATTGCCGAGGACGTTGACCCCGAGGAGATAGTTGCCCACCTTCCGCCGCTCTGTGAGGAGAAGGAGATCCCGTACATCTACGTTCCGAGCAAGAAGGAGCTCGGTGCGGCCGCCGGGCTTGAGGTCGCCGCTGCGAGCGT 496

TK1311 AGGAAGGGCACCAACGAGACCACCAAGGCCGTCGAGAGGGGCCAGGCCAAGCTCGTTATCATAGCCGAGGACGTTGACCCTGAAGAGATCGTTGCCCACCTCCCGCCGCTCTGCGAGGAGAAGGAGATCCCGTACGTCTACGTCCCGAGCAAGAAGGAGCTTGGAGCGGCCGCTGGCCTTGAAGTCCCAGCTGCCAGCGT 502

PFC_06055 AGAAAGGGAACAAATGAAACCACAAAGGCTGTAGAGAGAGGCCAAGCAAAGCTCGTCATAATTGCCGAGGATGTTGATCCAGAAGAGATTGTAGCACACCTTCCACCACTCTGTGAGGAGAAGGAGATTCCCTACATCTATGTTCCAAGTAAAAAGGAGCTTGGAGCTGCAGCTGGCATTGAAGTTGCAGCCGCAAGTGT 496

PF1367 AGAAAGGGAACAAATGAAACCACAAAGGCTGTAGAGAGAGGCCAAGCAAAGCTCGTCATAATTGCCGAGGATGTTGATCCAGAAGAGATTGTAGCACACCTTCCACCACTCTGTGAGGAGAAGGAGATTCCCTACATCTATGTTCCAAGTAAAAAGGAGCTTGGAGCTGCAGCTGGCATTGAAGTTGCAGCCGCAAGTGT 499

PYCH_03310 AGGAAGGGTACCAACGAGACCACCAAGGCCGTCGAGAGGGGCCAGGCCAAGCTCGTTATAATAGCTGAAGACGTTGACCCTGAGGAGATAGTCGCCCACCTCCCGCCGCTCTGTGAGGAGAAGGAGATACCCTACATCTACGTTCCGAGCAAGAAGGAGCTCGGTGCCGCGGCCGGTATCGAGGTCGCGGCCGCGAGCGT 496

PNA2_0082 AGGAAGGGTACGAACGAGACAACGAAGGCCGTCGAGAGAGGTCAAGCGAAGCTCGTCATAATAGCTGAGGATGTCGATCCAGAGGAGATCGTTGCACACCTTCCACCGCTCTGTGAGGAGAAGGAGATTCCCTACATCTACGTTCCAAGCAAGAAGGAGCTCGGTGCAGCTGCTGGAATTGAGGTTGCAGCTGCAAGCGT 499

PAB0460 AGGAAGGGAACCAACGAGACAACGAAAGCCGTTGAGAGGGGCCAGGCCAAGCTCGTTATAATAGCTGAGGACGTTGACCCAGAGGAGATAGTTGCCCACCTTCCACCGCTCTGTGAGGAGAAGGAGATACCCTACATCTACGTCCCAAGCAAGAAGGAGCTTGGAGCTGCAGCCGGAATTGAGGTTGCAGCCGCAAGCGT 511

PH1496 AGGAAGGGAACAAACGAAACTACAAAGGCCGTAGAGAGGGGTCAAGCTAAGCTTGTAATAATTGCAGAGGATGTCGACCCTGAGGAAATTGTAGCCCACTTACCACCACTCTGTGAGGAGAAGGAGATACCTTACATCTACGTTCCAAGCAAGAAGGAGCTTGGAGCTGCGGCCGGTATTGAAGTCGCTGCTGCTAGTGT 499

Metin_1393 AGCTATTATAAAGCCAGCAAATGAAGAAGAGTTAAATGCACTAATTGAAAAAATAAATGCTCTTAAACAATAA------------------------------------------------------------------------------------------------------------------------------- 554

Mefer_1103 AGCTATAGTTAACGAAGGAAACGCTGACGAGTTAAAGGCATTAATTGAAAAAATAAATGCTTTGAAACAGTAA------------------------------------------------------------------------------------------------------------------------------- 554

Metvu_1339 TGCAATTGTTAAAGAAGGAGATGCTGATGAGTTAAAGGCATTAATTGAAAAAATAAATGCTTTAAAAGAATAA------------------------------------------------------------------------------------------------------------------------------- 554

MFS40622_0835 TGCTATCATCAACGAAGGAAATGCTGAAGAGTTAAAGGCATTAATTGAAAAAGTAAATGCTTTGAAGCAGTAA------------------------------------------------------------------------------------------------------------------------------- 554

MJ1203 TGCTATCATCAACGAAGGAGATGCTGAAGAGTTAAAGGTATTAATTGAAAAGGTAAATGTTTTGAAGCAGTAA------------------------------------------------------------------------------------------------------------------------------- 554

Metig_1662 AGCTATCATAAACGAAGGAAATGCAGACGAATTAAAAGATTTAATCGAAAAAGTCAATGCATTGAAACAATAA------------------------------------------------------------------------------------------------------------------------------- 554

Maeo_0081 AGCAATTATCGCAGAAAAAGATGCAAATGCTTTAAAAGACTTAGTAGAAAAAATAAACGGTTTAAAGGCATAA------------------------------------------------------------------------------------------------------------------------------- 554

Metok_1174 AGCTGTTATAGCTGAGGGAGATTCAAACGCATTAAAAGAATTGGTAGAAAAATTAAACGCATTAAAAGCATAA------------------------------------------------------------------------------------------------------------------------------- 554

Mvol_0393 TGCTATTATCGAAACCGAAGATGCTGAAGGATTAAACGAGTTAGTTGAAAAAGTTAACGCTTTAAAAGCATAA------------------------------------------------------------------------------------------------------------------------------- 554

Mevan_1553 TGCAGTTGTTGCTGAAGGCAGCGCTGAACAATTAAAAGACTTAGTTGAAAAATTAAACGGTTTAAAAGCATAA------------------------------------------------------------------------------------------------------------------------------- 611

MmarC5_0963 TGCAGTTGTTGCTGAAGGAAACGCTGACGAATTAAAAGATTTAGTTGAAAAATTAAATGGATTAAAAGCATAA------------------------------------------------------------------------------------------------------------------------------- 611

GYY_03255 TGCAGTTGTTGCTGAAGGAAGCGCTGACGAATTAAAAGATTTAGTTGAAAAATTAAATGGATTAAAAGCATAA------------------------------------------------------------------------------------------------------------------------------- 554

MMP0641 TGCAGTTGTTGCTGAAGGAAGCGCTGACGAATTAAAAGATTTAGTTGAAAAATTAAATGGATTAAAAGCATAA------------------------------------------------------------------------------------------------------------------------------- 554

MmarC6_0248 TGCAGTTGTTGCTGAAGGAAGCGCTGACGAATTAAAAGATTTAGTTGAAAAATTAAATGGATTAAAAGCATAA------------------------------------------------------------------------------------------------------------------------------- 611

MmarC7_1665 TGCAGTTGTTGCTGAAGGAAGCGCTGACGAATTAAAAGATTTAGTTGAAAAATTAAATGGATTAAAAGCATAA------------------------------------------------------------------------------------------------------------------------------- 611

Mlab_1497 AGCAATCGTTAAAGTCGGCAAAGGCAAAGAGCTCGTAGGAGATCTTGCCGGTCAGATCAAGGCACTGAGAGGATAA---------------------------------------------------------------------------------------------------------------------------- 569

Mpet_2104 CGCAATCGTCAAACCCGGAAAGGCAAAAGAACTCCTTGACGAAGTTGTCGGACAGATCTCCGAACTCAGGCAGTGA---------------------------------------------------------------------------------------------------------------------------- 569

Mhun_1601 AGCTATCGTAAAGTCCGGAAAAGCAAAAGAGACCATCGATGAAATTGCTGCCCAGATAGCAGCTCTCAAAGCTGAGTGA------------------------------------------------------------------------------------------------------------------------- 569

Memar_0721 CGCGATCGTCAAGTCCGGCAAGGCAAAGGACCTCGTCGAAGAGATCGCGAAGCAGATTGCTGCACTGAGAGAGTGA---------------------------------------------------------------------------------------------------------------------------- 569

Mboo_0690 AGCGATTGTCAAGCCCGGCAAAGCAAAAGAAGTGGTTGAAGATCTCGCAAAGCAGCTGACTGCATTGAAGGCATAA---------------------------------------------------------------------------------------------------------------------------- 569

Mpal_0523 AGCGATTGTGAAGCCTGGAAAGGCAAAAGAACTCGTCGACGAGATCACGGCCCAGCTCACTGAGTTAAGGGGGTAA---------------------------------------------------------------------------------------------------------------------------- 569

MCP_1041 GGCCATCGTGGACGCCGGCAAGGCAAAGGCCCTGGTCGACGACATCGCCCAGAAGTTCGTGGCCCTGAAGAAATAA---------------------------------------------------------------------------------------------------------------------------- 572

LRC145 CGCCATCGTGGACGCAGGCAAGGCTAAGGCCCTGGTCGACGAGATCACACAGAAGTTCAGCGCTCTGAAGGCATAA---------------------------------------------------------------------------------------------------------------------------- 572

MCON_1706 TGCGATCGTGGAGCCGGGGAAAGGAAAAGAGATTCTGGATGAGATCGTCCAAAAAATTGGATCTCTGAAGTAG------------------------------------------------------------------------------------------------------------------------------- 569

Mthe_0331 TGCGATAATAGAGCCGGGAAAGGGAAAGGAGCTGTTGGAGGAGATAATCCAGAAACTCCAGGCTCTGAAGTAG------------------------------------------------------------------------------------------------------------------------------- 569

Mbar_A3388 CGCAATCGTAGATGCAGGAAAAGCAGGTGAAATGGTCCAGGACATTGCCCAGAAACTTGAAGCCCTTAAATAA------------------------------------------------------------------------------------------------------------------------------- 563

MA1521 GGCAATTGTGGATGCAGGTAAAGCAGCTGAAATGGTCCAGGACATCGCTCAGAAACTTGAAGCTCTTAAATAA------------------------------------------------------------------------------------------------------------------------------- 563

MM_2467 GGCAATTGTAGATGCAGGCAAGGCAGCTGAAATGATTCAGGACATCGCTCAGAAGCTTGACGCTCTTAAATAA------------------------------------------------------------------------------------------------------------------------------- 563

Mzhil_1258 TGCTATCACCGATGCTGGTAAAGGTAAAGAGACGATTGATGATATCGTCGAAAAAATGAGTTCACTTAAATAA------------------------------------------------------------------------------------------------------------------------------- 557

Metev_1406 TGCTATTACTGATTCTGGCAAAGGAAACGAAATCATAAAAGATATTGCTGAAAAAGTTGAATCATTAAAATAA------------------------------------------------------------------------------------------------------------------------------- 554

Mbur_0219 TGTGATCACTGATGCAGGAAAGGGCAGCGAACTTGTAGAAGACGTTGCGCAGAAAGTCGCTGCACTCAAATAA------------------------------------------------------------------------------------------------------------------------------- 554

Mmah_0863 AGTAATTACAGATGCAGGTAAGGGCGCAGAGACCATCGAAGACCTGGCCGAAAAAGTCAGTGCTCTCAAATAA------------------------------------------------------------------------------------------------------------------------------- 554

Mfer_0452 AGCCATAATAGATGCAGGTGAAGCTGAAGATCTCACAAATGAAATTATAGAAAAAATTGAAAGCATTAAAAAATAA---------------------------------------------------------------------------------------------------------------------------- 572

MTBMA_c07050 AGCCATAGTTGAAGCCGGCGACGCCGAGGACCTCATAAACGAAATAATAGAAAAGGTTGAAGAACTTAAAAAATAG---------------------------------------------------------------------------------------------------------------------------- 572

MTH255 AGCCATCGTTGAGGCAGGCGACGCCGAGGACCTCATAAAAGAGATAATAGAAAAGGTCGAAGAACTTAAGAAATAG---------------------------------------------------------------------------------------------------------------------------- 593

mru_1490 TTGTATTGTCGATGCTGGAGACGCTGCTGACTTAGTAGAAGAAGTTGTTGAAAAAGTAGCAGAACTTAAAGAATAA---------------------------------------------------------------------------------------------------------------------------- 608

Msm_0206 TTGTATTGTTGACGCTGGAGATGCTGAAGGCGACGTAGCTGAAATTGTTGAAAAAATCGCAGAATTAAAAGAATAA---------------------------------------------------------------------------------------------------------------------------- 572

MSWAN_1751 ATGTATAACAGATGCCGGTGAAGCTGAAGATTTAATCAACGACATTGTTGAGAAAGTCGAAGAACTCAAAAAATAG---------------------------------------------------------------------------------------------------------------------------- 572

Msp_0633 ATGTATTATTGATGCTGGTGAAGGCCAAGAACTCGTCGATGAAATAGTCGAAAAAGTTGCAGAACTCAAAAACTAA---------------------------------------------------------------------------------------------------------------------------- 572

Faci_050500008291 AGTGGTTGATTTCGGTAAAAATGAGGAT---GCATATAAGGAACTTATATCATCAATAGAAAATGCAAAAGCTGGAAAGGAAGAAAAGAAGAAACCTGCAGCCAAGTCAGAGAAACCAAAAGAGGAAGCAGTTGAAGCTGAGGCTGCAGAAGCACCTGAAGCAGAAAAAGAAGCCAAGAAGGAAGCAAAAAAGGAGCCAA 690

PTO1280 AATAGTTGACTATGGAAAAAATGATGAT---GCATACAAATCGATAGTTTCACAGATAAACGATGCAAAGTCCGGAAAATCAGAGAATAAGGAGTGA------------------------------------------------------------------------------------------------------- 584

Ta1116 CATAGTTGACTATGGAAAGAATGAGGAA---CTCTACAAATCCATAGTTTCTGCTCTGGAGCAGATAAAGAAGTGA---------------------------------------------------------------------------------------------------------------------------- 566

TVN0449 GATTGTTGACTACGGCAAGAACGAGGAA---CTTTATAAGTCCATAGTCTCCGCCGTAGAACAGTTGAAGAAGTAA---------------------------------------------------------------------------------------------------------------------------- 566

Arcpr_0494 ATGCATCATCGATGAAGGAGAAGCCAAGAAAGAGCTCAAGGATCTGATTGAGAAGCTTAGAGGATTGAGGAAGTAA---------------------------------------------------------------------------------------------------------------------------- 563

Ferp_0621 AGCGATAATTAACGAAGGAGAAGCGAAGAAGGAACTCGAAGAATTGATTAAGAAGATTAACGCTTTGAAGAAGTAA---------------------------------------------------------------------------------------------------------------------------- 566

AF0764 AGCGATAATCAACGAGGGAGAGCTGAGAAAGGAGCTTGGAAGCCTTGTGGAGAAGATTAAAGGCCTTCAGAAGTAA---------------------------------------------------------------------------------------------------------------------------- 560

Arcve_0453 GGCAATCATAAACGAGGGAGAAGCGAAGAAGGAACTCTCGCAGATCGTTAGCAAGCTTGAAGGATTGAAGAAATAA---------------------------------------------------------------------------------------------------------------------------- 560

Aboo_1236 -AGCATCCTTGACTTTGGAAAAGCTAATGATTCTTTTAAGGAAATAATCGAGCAGATTAAGGCAATAAAGAAGTAA---------------------------------------------------------------------------------------------------------------------------- 566

HQ2885A TGCCATCATCGACGCTGGCGAGGCAGACAGTGATGTCGATGATATCACAGCAAAGATTGAGGAACTTAGATAA------------------------------------------------------------------------------------------------------------------------------- 563

VNG1157G CGCCGTCGTCGCCGCCGGCGACGCCGAAGACGAAATCGAGGACATCTCGACGAAAGTCGAGGACCTCCAATAA------------------------------------------------------------------------------------------------------------------------------- 563

OE2662F CGCCGTCGTCGCCGCCGGCGACGCCGAAGACGAAATCGAGGACATCTCGACGAAAGTCGAGGACCTCCAATAA------------------------------------------------------------------------------------------------------------------------------- 563

Hlac_1842 CGCCGTCATCGACGCCGGTGACGCCGACGACGACGTCGAGGATATCGGCGAGAAGGTCGCGGAGCTCCGATAA------------------------------------------------------------------------------------------------------------------------------- 563

Halar_2173 CGGCATCGTCGACGCCGGCGACGCCGAGGAGGACGTCGAGGACATCGGCGAGAAGGTCGAGGAGCTTCGATAA------------------------------------------------------------------------------------------------------------------------------- 563

HVO_2737 CGCCATCGTCGACGCCGGCGACGCGTCCGACGATGTCGAGGACATCGCGACTAAGGTCGAGGACCTTCGATAA------------------------------------------------------------------------------------------------------------------------------- 563

Hbor_12060 CGCCATCGTCGACGCTGGCGAGGCACAGGAGCAGGTCGAAGACATCGCCGCGAAGGTCGAGGAACTTCAGTAA------------------------------------------------------------------------------------------------------------------------------- 572

HacjB3_08675 CGCGATCACGGACGCCGGCGAGGCCGAAAGCGAGGTCGAGGACATCGGGAAGAAGGTCGAGGACCTTCGCTGA------------------------------------------------------------------------------------------------------------------------------- 563

Nmag_0451 AGCCATCGTCGACACCGGAGAGGCCGAAGGCGACGTCGAAGACATCGCCGGCAAGGTCGAGGACCTCGACTGA------------------------------------------------------------------------------------------------------------------------------- 563

Halxa_0717 GGCGATCGTCGACGCCGGCGAGGCCTCCGGCGACGTCGAGGACATCGCCGACAAGGTCGAGGACCTCGACTGA------------------------------------------------------------------------------------------------------------------------------- 563

Htur_2527 GGCGATCGTCGACGCCGGCGAGGCTTCCGACGACGTCGAAGATATCGCCGACAAGGTCGAGGACCTCGACTGA------------------------------------------------------------------------------------------------------------------------------- 563

NP3660A GGCCATCGTCGATGCCGGCGAGGCGAACGGCGAGGTCGAGGATATCGCCGAGAAAGTTGAGGAACTTCAGTAG------------------------------------------------------------------------------------------------------------------------------- 563

Huta_0750 CGCGATCGTCGATGCGGGCGACGCCGACGGCGACGTCGAGGACATCGCCGGGAAGGTCGAGGATCTCCGCTGA------------------------------------------------------------------------------------------------------------------------------- 563

Hmuk_2621 GGCAGTCGTCGACGCCGGTGAGGCGGAAGACGACGTCGAGGACATTGCCAGCAAGGTCGAGGAACTTCGGTGA------------------------------------------------------------------------------------------------------------------------------- 563

HAH_0858 AGCCGTCACCGACGCCGGTGAGGCTGACGCCGATGTCGAGGACATCGCCGACAAGGTCGAGGAGCTTCGGTGA------------------------------------------------------------------------------------------------------------------------------- 563

rrnAC0103 AGCCGTCACCGACGCCGGTGAGGCTGACGCCGATGTCGAGGACATCGCCGACAAGGTCGAGGAGCTTCGGTGA------------------------------------------------------------------------------------------------------------------------------- 563

MK1598 GTGCATCATCGATCCCGGAGACGCCAAGGACCTCGTGGACGAGATCATTGAGAAGGTCGAGGAGCTACGTGAGTAA---------------------------------------------------------------------------------------------------------------------------- 572

TERMP_01118 TGCTATAATCGAGCCGGGTAAGGCTCGTGAGCTTGTTGAGGAAATCGCTATGAAGGTTAGGGAGCTTATGAAGTGA---------------------------------------------------------------------------------------------------------------------------- 602

TSIB_0419 TGCTATAATTGAGCCCGGTAAGGGTCGTGAGCTTGTTGAGGAAATTGCAATGAAAGTTAGGGAGCTTGCTAAGTGA---------------------------------------------------------------------------------------------------------------------------- 587

GQS_00200 AGCCATCGTTGAGCCCGGCAAGGGCCGCGAGCTCGTTGAGGACATCGCTATGAAGGTCAGGGAGCTCATGAAGTGA---------------------------------------------------------------------------------------------------------------------------- 572

TON_0954 CGCCATCATCGAGCCCGGCAAGGGCAGGGAGCTCGTTGAGGAAATCGCTATGAAGGTTAGGGAGCTCATGAAGTGA---------------------------------------------------------------------------------------------------------------------------- 572

TGAM_1876 GGCAATAATAGAGCCCGGCAAGGCCCGCGAGCTCGTTGAGGACATTGCTATGAAGGTCAAGGAGCTCATGAAGTGA---------------------------------------------------------------------------------------------------------------------------- 572

TK1311 TGCTATCATCGAGCCGGGCAAGGCCCGCGAGCTCGTTGAGGACATCGCCATGAAGGTCAAGGAGCTCATGAAGTGA---------------------------------------------------------------------------------------------------------------------------- 578

PFC_06055 TGCCATAATTGAACCAGGTAAAGCGAGAGATTTAGTTGAAGAGATAGCCATGAAAGTTAAGGAATTAATGAAGTGA---------------------------------------------------------------------------------------------------------------------------- 572

PF1367 TGCCATAATTGAACCAGGTAAAGCGAGAGATTTAGTTGAAGAGATAGCCATGAAAGTTAAGGAATTAATGAAGTGA---------------------------------------------------------------------------------------------------------------------------- 575

PYCH_03310 GGCTATAATCGAGCCCGGCAAGGCTAAGGAGCTCGTTGAGGAAATCGCCATGAAGGTTAGGGAGCTCATGAAGTGA---------------------------------------------------------------------------------------------------------------------------- 572

PNA2_0082 TGCTATAATTGACCCTGGCAAGGCGAGAGATTTAGTTGAAGAGATTGCAATGAAAGTTAGGGAGCTCATGAAGTGA---------------------------------------------------------------------------------------------------------------------------- 575

PAB0460 AGCTATAATCGAGCCTGGTAAGGCGAGAGATTTGGTTGAAGAGATTGCAATGAAGGTTAGGGAGCTCATGAAGTGA---------------------------------------------------------------------------------------------------------------------------- 587

PH1496 GGCTATAATCGAGCCTGGCAAGGCGAGAGATTTAGTTGAAGAGATCGCCATGAAGGTTAGGGAGCTCATGAAGTGA---------------------------------------------------------------------------------------------------------------------------- 575

Metin_1393 -------------------------------------------------------------------------------------------- 554

Mefer_1103 -------------------------------------------------------------------------------------------- 554

Metvu_1339 -------------------------------------------------------------------------------------------- 554

MFS40622_0835 -------------------------------------------------------------------------------------------- 554

MJ1203 -------------------------------------------------------------------------------------------- 554

Metig_1662 -------------------------------------------------------------------------------------------- 554

Maeo_0081 -------------------------------------------------------------------------------------------- 554

Metok_1174 -------------------------------------------------------------------------------------------- 554

Mvol_0393 -------------------------------------------------------------------------------------------- 554

Mevan_1553 -------------------------------------------------------------------------------------------- 611

MmarC5_0963 -------------------------------------------------------------------------------------------- 611

GYY_03255 -------------------------------------------------------------------------------------------- 554

MMP0641 -------------------------------------------------------------------------------------------- 554

MmarC6_0248 -------------------------------------------------------------------------------------------- 611

MmarC7_1665 -------------------------------------------------------------------------------------------- 611

Mlab_1497 -------------------------------------------------------------------------------------------- 569

Mpet_2104 -------------------------------------------------------------------------------------------- 569

Mhun_1601 -------------------------------------------------------------------------------------------- 569

Memar_0721 -------------------------------------------------------------------------------------------- 569

Mboo_0690 -------------------------------------------------------------------------------------------- 569

Mpal_0523 -------------------------------------------------------------------------------------------- 569

MCP_1041 -------------------------------------------------------------------------------------------- 572

LRC145 -------------------------------------------------------------------------------------------- 572

MCON_1706 -------------------------------------------------------------------------------------------- 569

Mthe_0331 -------------------------------------------------------------------------------------------- 569

Mbar_A3388 -------------------------------------------------------------------------------------------- 563

MA1521 -------------------------------------------------------------------------------------------- 563

MM_2467 -------------------------------------------------------------------------------------------- 563

Mzhil_1258 -------------------------------------------------------------------------------------------- 557

Metev_1406 -------------------------------------------------------------------------------------------- 554

Mbur_0219 -------------------------------------------------------------------------------------------- 554

Mmah_0863 -------------------------------------------------------------------------------------------- 554

Mfer_0452 -------------------------------------------------------------------------------------------- 572

MTBMA_c07050 -------------------------------------------------------------------------------------------- 572

MTH255 -------------------------------------------------------------------------------------------- 593

mru_1490 -------------------------------------------------------------------------------------------- 608

Msm_0206 -------------------------------------------------------------------------------------------- 572

MSWAN_1751 -------------------------------------------------------------------------------------------- 572

Msp_0633 -------------------------------------------------------------------------------------------- 572

Faci_050500008291 AGAAAGCAAAAACTTCTGAATCAAAGGAAACAAAGAAAGCCCCTAAAAAGACTACAAAGAAGAAAGAAGATTCAGAAGAAGATAAAGAGTGA 782

PTO1280 -------------------------------------------------------------------------------------------- 584

Ta1116 -------------------------------------------------------------------------------------------- 566

TVN0449 -------------------------------------------------------------------------------------------- 566

Arcpr_0494 -------------------------------------------------------------------------------------------- 563

Ferp_0621 -------------------------------------------------------------------------------------------- 566

AF0764 -------------------------------------------------------------------------------------------- 560

Arcve_0453 -------------------------------------------------------------------------------------------- 560

Aboo_1236 -------------------------------------------------------------------------------------------- 566

HQ2885A -------------------------------------------------------------------------------------------- 563

VNG1157G -------------------------------------------------------------------------------------------- 563

OE2662F -------------------------------------------------------------------------------------------- 563

Hlac_1842 -------------------------------------------------------------------------------------------- 563

Halar_2173 -------------------------------------------------------------------------------------------- 563

HVO_2737 -------------------------------------------------------------------------------------------- 563

Hbor_12060 -------------------------------------------------------------------------------------------- 572

HacjB3_08675 -------------------------------------------------------------------------------------------- 563

Nmag_0451 -------------------------------------------------------------------------------------------- 563

Halxa_0717 -------------------------------------------------------------------------------------------- 563

Htur_2527 -------------------------------------------------------------------------------------------- 563

NP3660A -------------------------------------------------------------------------------------------- 563

Huta_0750 -------------------------------------------------------------------------------------------- 563

Hmuk_2621 -------------------------------------------------------------------------------------------- 563

HAH_0858 -------------------------------------------------------------------------------------------- 563

rrnAC0103 -------------------------------------------------------------------------------------------- 563

MK1598 -------------------------------------------------------------------------------------------- 572

TERMP_01118 -------------------------------------------------------------------------------------------- 602

TSIB_0419 -------------------------------------------------------------------------------------------- 587

GQS_00200 -------------------------------------------------------------------------------------------- 572

TON_0954 -------------------------------------------------------------------------------------------- 572

TGAM_1876 -------------------------------------------------------------------------------------------- 572

TK1311 -------------------------------------------------------------------------------------------- 578

PFC_06055 -------------------------------------------------------------------------------------------- 572

PF1367 -------------------------------------------------------------------------------------------- 575

PYCH_03310 -------------------------------------------------------------------------------------------- 572

PNA2_0082 -------------------------------------------------------------------------------------------- 575

PAB0460 -------------------------------------------------------------------------------------------- 587

PH1496 -------------------------------------------------------------------------------------------- 575

Archaeglobales, Halobacteriales Haloferacales Natrialbales Methanobacteriales Methanococcales Methanocellales Methanomicrobiales Methanosarcinales Methanopyrales Thermococcales Thermoplasmatales Unclassified Euryarchaeota
