# Supplementary material for: RIP-Seq Suggests Translational Regulation by L7Ae in Archaea
Source: mBio. 2017 Aug 1;8(4):e00730-17. doi: 10.1128/mBio.00730-17 (PMC5539422; doi:10.1128/mBio.00730-17)
Supplement: TEXT S2 [file mbo004173413s2.docx]

Supplementary File 2. Oligonucleotides for cloning

Primers to generate pSVA406-Saci_1520CFHA for the genomic Flag-HA tagging of Saci_1520 (*l7ae*):

Saci_1520 C-FHA LF for: 5'­tagcgtCCGCGGccaaaggagtttatcatagacatcccacag-3' (SacII)

Saci_1520 C-FHA LF rev: 5'­GGCGTAGTCTGGAACATCGTAAGGGTAGCTCTTAACCTTGTCATCGTCGTCCTTGTAGTCactacttttaccttttatttcattaactct-3' (Flag-tag/HA-tag)

Saci_1520 C-FHA RF for:
5'­GACTACAAGGACGACGATGACAAGGTTAAGAGCTACCCTTACGATGTTCCAGACTACGCCTGAaaatttttgttataactaatattttta-3' (Flag-tag/HA-tag)

Saci_1520 C-FHA RF rev: 5'­ttggctGTCGACtccgcctattcctagacctg-3' (SalI)

Primers to generate pSVA406-Saci0259KO for the genomic deletion of Saci_0259 (*sac-sR10*):

CD11KO LF for: 5'­attctgCCATGGGCTTAGTATTCGCTATTCCT-3'(NcoI)

CD11KO LF rev: 5'­tttctcattacagatcaaatacaattaggagtacttag-3' (left flank/right flank)

CD11KO RF for: 5'­ctaagtactcctaattgtatttgatctgtaatgagaaa-3' (left flank/right flank)

CD11KO RF rev: 5'­tgtagtGTCGACCTGTTATTGGATGTTCAACT-3' (SalI)

Primers for the site-directed mutagenesis of pSVA406-Saci_1520 to generate genomic *l7ae* 5' UTR mutations:

**Kt-n mut1 mutation**

5UTR g539c for: 5'­gatagagacaatctattttcgcgtcacgaaaaagaaggagg-3' (mutation)

5UTR g539c rev: 5'­cctccttctttttcgtgacgcgaaaatagattgtctctatc-3' (mutation)

**double Kt-n mut mutation**

c539_a540t for: 5'­gacaatctattttcgcgtctcgaaaaagaaggaggatga-3' (mutation)

c539_a540t rev: 5'­tcatcctccttctttttcgagacgcgaaaatagattgtc-3' (mutation)

Primers to generate pNat, pKt-n mut1 and pBTmut for the β-galactosidase reporter assays in *S. acidocaldarius*:

L7PR Nat for:

5'­tagcgtCCGCGGTCATATTCTCACATGAATAActttttaatggatagagacaatctattttcgcgtgacgaaaaagaaggaggatgaacgctGTGTACTCATTTCCAAATAGCTT-3' (SacII/BRE/TATA/Dbox-like/Cbox-like/*lacS* start)

L7PR Dmut for:

5'­tagcgtCCGCGGTCATATTCTCACATGAATAActttttaatggatagagacaatctattttcgcgtcacgaaaaagaaggaggatgaacgctGTGTACTCATTTCCAAATAGCTT-3' (SacII/BRE/TATA/Dbox-like/Cbox-like/mutation/*lacS* start)

L7PR BTmut for: 5'­tagcgtCCGCGGTCATATTCTCACATGGCCGCcgggcgccgggatagagacaatctattttcgcgtgacgaaaaagaaggaggatgaacgctGTGTACTCATTTCCAAATAGCTT-3' (SacII/BRE/TATA/Dbox-like/Cbox-like/mutation/*lacS* start)

L7PR HA new rev:

5'­agccaaCGGCCGTTAGGCGTAGTCTGGAACATCGTAAGGGTAGTGCCTTAATGGCTTTAC-3' (EagI/*lacS* end/HA-Tag)

Primers for the site-directed mutagenesis of pNat to generate pKt-n mut 2:

L7PR Dmut2 for: 5'­gacaatctattttcgcgtggcgaaaaagaaggaggatga-3' (mutation)

L7PR Dmut2 rev: 5'­tcatcctccttctttttcgccacgcgaaaatagattgtc-3' (mutation)

Primers to generate pEC-A-His-Sumo-L7Ae for the heterologous expression of L7Ae in *E. coli*:

Saci1520fw: 5'­ACCAGGAACAAACCGGCGGCCGCTCGATGTCTAAACCCTCGTATG-3' (LIC 5'/*l7ae* start)

Saci1520rev: 5'­GCAAAGCACCGGCCTCGTTAACTACTTTTACCTTTTATTTCATTAACTC-3' (LIC 3'/*l7ae* end)

Primers to generate pMD-auto*l7ae*-*gfp* from pEC-A-His-Sumo-L7Ae for GFP reporter assays in *E. coli*:

1. Inverse PCR primers for the removal of the His-Sumo tag:

Inv PCR L7Ae for: 5'­ATGTCTAAACCCTCGTATGTAAAAT-3'

Inv PCR L7Ae rev: 5'­ATGTATATCTCCTTCTTAAAGTTAAAC-3'

1. Gibson Assembly primers for the cloning of the *l7ae* 5' UTR + *sfgfp* gene:

**pEC-A-L7Ae template**

pEC-A PCR for: 5'­CGGGACCAGTGACGAAGGCT-3'

ROP PCR rev: 5'­CCCACGGGTGCGCATGATCG-3'

**pASK-IBA3plus-sfgfp template**

Pcat UTR GFP for: 5'­CGATCATGCGCACCCGTGGGggcacgtaagaggttccaactttcaccataatgaaataagcgtgacgaaaaagaaggaggatgaacgctATGAGCAAAGGAGAAGAAC-3' (*l7ae* 5' UTR/*sfgfp* start)

T1 ter GFP rev: 5'­AGCCTTCGTCACTGGTCCCGataaaacgaaaggcccagtctttcgactgagcctttcgttttatttgatgcctggTTATTTTTCGAACTGCGGGT-3' (T1 terminator/Strep-tag)

1. Primers for the cloning of the pN25 promoter:

PN25 5UTR gfp fw: 5'­TATTTGCTTTCAGGAAAATTTTTCTGTATAATAGATTCGCGTGACGAAAAAGAAGGAG-3'(pN25)

ROP PCR rev: 5'­CCCACGGGTGCGCATGATCG-3'

1. Primers to clone the aKt upstream of the l7ae gene:

Inv PCR L7Ae for: 5'­ATGTCTAAACCCTCGTATGTAAAAT-3'

T7 L7AeUTR rev: 5'­agcgttcatcctccttctttttcgtcacgcATTATTTCTAGAGGGGAATTGTTATC-3' (*l7ae* UTR)

1. Site-directed mutagenesis primers for the frameshifting of the *l7ae* gene:

**no aKt**

del112: 5'­aactttaagaaggagatatacatatg-ctaaaccctcgtatgtaaaatttg-3' (mutation)

del112-antisense: 5'­caaattttacatacgagggtttag-catatgtatatctccttcttaaagtt-3' (mutation)

**with akt**

del112+UTR for: 5'­ggaggatgaacgctatg-ctaaaccctcgtatgta-3' (mutation)

del112+UTR rev: 5'­tacatacgagggtttag-catagcgttcatcctcc-3' (mutation)

1. Primers for the site-directed mutagenesis of the *gfp*Kt:

**bulge mut**

a4943t: 5'­tgacgaaaaagaaggaggttgaacgctatgagcaaag-3' (mutation)

a4943t_antisense: 5'­ctttgctcatagcgttcaacctccttctttttcgtca-3' (mutation)

**double Kt-n mut**

N25 doublmutD fw: 5'­aaaatttttctgtataatagattcgcgtctcgaaaaagaaggaggatgaacg-3' (mutation)

N25 doublmutD re: 5'­cgttcatcctccttctttttcgagacgcgaatctattatacagaaaaatttt-3' (mutation)

**double Kt-b mut**

doubleCmut for: 5'­tcgcgtgacgaaaaagaaggaggatctacgctatgagcaa-3' (mutation)

doubleCmut rev: 5'­ttgctcatagcgtagatcctccttctttttcgtcacgcga-3' (mutation)

1. Primers for the site-directed mutagenesis of the aKt:

**Kt-n mut1**

g83c: 5'­aattcccctctagaaataatgcgtcacgaaaaagaaggag-3' (mutation)

g83c_antisense: 5'­ctccttctttttcgtgacgcattatttctagaggggaatt-3' (mutation)

**double Kt-n mut**

g83c_a84t: 5'­caattcccctctagaaataatgcgtctcgaaaaagaaggaggatg-3' (mutation)

g83c_a84t_anti: 5'­catcctccttctttttcgagacgcattatttctagaggggaattg-3' (mutation)

1. Primers for the cloning of the control UTR

control UTR for: 5'­TATTTGCTTTCAGGAAAATTTTTCTGTATAATAGATTCaacatgtccaataataatggagtataacatATGAGCAAAGGAGAAGAACTTTTCACTGG-3' (pN25/control UTR)

ROP PCR rev: 5'­CCCACGGGTGCGCATGATCG-3'

1. Primers for the cloning of different archaeal *l7ae* upstream regions:

APE_1818 UTR fw: 5'­TATTTGCTTTCAGGAAAATTTTTCTGTATAATAGATTCgccacacccagattctagaggctgtgacgacctgaaaggggaggaggagccATGAGCAAAGGAGAAGAACTTTTCACTGG-3' (pN25/A. pernix *l7ae* upstream region)

Smar_0825 UTR fw: 5'­TATTTGCTTTCAGGAAAATTTTTCTGTATAATAGATTCattatttggttttaaatcagacgaccgaagagttgggaggaggatgaagttATGAGCAAAGGAGAAGAACTTTTCACTGG-3' (pN25/S. marinus *l7ae* upstream region)

PAE3347 UTR fw: 5'­TATTTGCTTTCAGGAAAATTTTTCTGTATAATAGATTCgttacgcgcagaggcataaagatttataaagccagttctttgtggcaaccATGAGCAAAGGAGAAGAACTTTTCACTGG-3' (pN25/P. aerophilum *l7ae* upstream region)

AF0764 UTR fw: 5'­TATTTGCTTTCAGGAAAATTTTTCTGTATAATAGATTCggaattagggaagagccgatgaatgatgatttgatgaaggaggtgatgacATGAGCAAAGGAGAAGAACTTTTCACTGG-3' (pN25/A. fulgidus *l7ae* upstream region)

HVO_2737 UTR fw: 5'­TATTTGCTTTCAGGAAAATTTTTCTGTATAATAGATTCgtgataccggcgagggagtcgagcccgcgagcaggatataggtgaacaacaATGAGCAAAGGAGAAGAACTTTTCACTGG-3' (pN25/H. volcanii *l7ae* upstream region)

MA1521 UTR fw: 5'­TATTTGCTTTCAGGAAAATTTTTCTGTATAATAGATTCacctatgattgatgaaaaattgcgagacccgcaatttcgaaggagaaacttaaATGAGCAAAGGAGAAGAACTTTTCACTGG-3' (pN25/M. acetivorans *l7ae* upstream region)

TK1311 UTR fw: 5'­TATTTGCTTTCAGGAAAATTTTTCTGTATAATAGATTCgcccgagtcggtggtgcaaacagatgaacgatgaggtttcggagggATGAAGATGAGCAAAGGAGAAGAACTTTTCACTGG-3' (pN25/T. kodakarensis *l7ae* upstream region)

MmarC5 UTR fw: 5'­TATTTGCTTTCAGGAAAATTTTTCTGTATAATAGATTCGTGGGCGCCATCCGGATAAAAAAATTTTTTTAAGTGATGAAGGAGGTCATAATATGAGCAAAGGAGAAGAACTTTTCACTGG-3' (pN25/M. maripaludis *l7ae* upstream region)

ROP PCR rev: 5'­CCCACGGGTGCGCATGATCG-3'

1. Primers for the cloning of the Saci_1468 and Saci_2027 mRNA k-turns downstream of the start codon of the *sfgfp* gene:

Kt_1468 for: 5'­ccaataataatggagtataacatATGGAATATGATGAAGAGTTAGATGAACTTCTGAAAAAAAGAGCAACAGAGAGCAAAGGAGAAGAACTTTTCACTG-3' (*sfgfp* start/Saci_1468 mRNA Kt)

Kt_2027 for: 5'­ccaataataatggagtataacatATGACTGATGATGACGCTATACCCTCTGACACGTGGATCCAAAGCAAAGGAGAAGAACTTTTCACTG-3' (*sfgfp* start/Saci_2027 mRNA Kt)

mRNA KT 2nd for: 5'­TATTTGCTTTCAGGAAAATTTTTCTGTATAATAGATTCaacatgtccaataataatggagtataacatATG-3' (pN25/control UTR)

ROP PCR rev: 5'­CCCACGGGTGCGCATGATCG-3'

Oligonucleotides for hybridization to generate PCR templates for *in vitro* transcription:

**Nat and Kt-n mut RNA**

L7P Nat hyb for:
5'­gctcgaattcTAATACGACTCACTATAgcgtgacgaaaaagaaggaggatgaacgctGTGTCTAAACCCTCGTATGTAAAATTTGAAtctagagctc-3' (EcoRI/T7 promoter/*l7ae* UTR/*l7ae* CDS/XbaI)

L7P Nat hyb rev: 5'­gagctctagaTTCAAATTTTACATACGAGGGTTTAGACACagcgttcatcctccttctttttcgtcacgcTATAGTGAGTCGTATTAgaattcgagc-3' (EcoRI/T7 promoter/*l7ae* UTR/*l7ae* CDS/XbaI)

L7P Dmut hyb for: 5'­gctcgaattcTAATACGACTCACTATAgcgtcacgaaaaagaaggaggatgaacgctGTGTCTAAACCCTCGTATGTAAAATTTGAAtctagagctc-3' (EcoRI/T7 promoter/*l7ae* UTR/mutation/*l7ae* CDS/XbaI)

L7P Dmut hyb rev: 5'­gagctctagaTTCAAATTTTACATACGAGGGTTTAGACACagcgttcatcctccttctttttcgtgacgcTATAGTGAGTCGTATTAgaattcgagc-3' (EcoRI/T7 promoter/*l7ae* UTR/mutation/*l7ae* CDS/XbaI)

**Sac-sR121 RNA**

CD39IvFor: 5'­GATCCTAATACGACTCACTATAGGGAGAACAGATGATGAACACTGGCTGTATTGACCAAATGATATAAAAACCTTCTAATGTCTGATATCTCA-3' (BamHI/T7 promoter/Sac-sR121/HindIII)

CD39IvRev: 5'­AGCTTGAGATATCAGACATTAGAAGGTTTTTATATCATTTGGTCAATACAGCCAGTGTTCATCATCTGTTCTCCCTATAGTGAGTCGTATTAG-3' (BamHI/T7 promoter/Sac-sR121/HindIII)

**SRP RNA**

SRP RNA hyb for: 5'­gctcgaattcTAATACGACTCACTATA*GTCTAACTATGATCAGGAGCGATAGGAGGAAGAC*tctagagctc-3' (EcoRI/T7 promoter/*SRP RNA Kt*/103-117 nt/245-259 nt/XbaI)

SRP RNA hyb rev: 5'­gagctctagaGTCTTCCTCCTATCGCTCCTGATCATAGTTAGACTATAGTGAGTCGTATTAgaattcgagc-3' (EcoRI/T7 promoter/*SRP RNA Kt*/103-117 nt/245-259 nt/XbaI)

**Nop5 mRNA**

Nop56L hyb for: 5'­gctcgaattcTAATACGACTCACTATAGAGAATTGATGAAGATATATTTAGTGGAACATGCAATAGGATCGTTTGGATATGACGAGAGCGGAAAATTAAT-3' (EcoRI/T7 promoter/SRP RNA Kt 1st half)

Nop56L Kt hyb rev:

5'­gagctctagaAATTAAAGCTTCAGTAACTTTTCCAATATCTTTACTATTTGGTACAAAATCTATTAATTTTCCGCTCTCGTCATATCCAAACGATCCTAT-3' (XbaI/T7 promoter/SRP RNA Kt 2nd half)

Primers for the PCR amplification of the hybridized oligonucleotides or cloned plasmids to generate run-off *in vitro* transcription templates:

**Nat and Kt-n mut1 RNA**

pUC19 PCR for: 5'­GCTGCAAGGCGATTAAG-3'

UTR+CDS PCR rev: 5'­TTCAAATTTTACATACGAGGGTTT-3'

**Sac-sR121 RNA**

pUC19IvFor: 5'­TGTGCTGCAAGGCGATTAAG-3'

CD39PCRrev: 5'­GAGATATCAGACATTAGAAGG-3'

**SRP RNA and Nop5 mRNA**

T7 PCR for: 5'­gctcgaattcTAATACGACTCACT-3'

SRP RNA PCR rev: 5'­GTCTTCCTCCTATCGCTCCT-3'

Nop56L PCR rev: 5'­AATTAAAGCTTCAGTAACTTTTCCAATATC-3'

Oligonucleotide for northern blot analysis:

Saci11probe: 5'­CGCTTTTTGTCATCATTCTCAGATCCCGGATTCCACATC-3'
